# Supplementary material for: Health outcomes after myocardial infarction: A population study of 56 million people in England
Source: PLoS Med. 2024 Feb 15;21(2):e1004343. doi: 10.1371/journal.pmed.1004343 (PMC10868847; doi:10.1371/journal.pmed.1004343)
Supplement: S1 Checklist — (DOCX) [file pmed.1004343.s001.docx]

# S1 Checklist: REporting of studies Conducted using Observational Routinely-collected Data (RECORD) Standard

|  | **Item No.** | **STROBE items** | **RECORD items** | **Location in manuscript where items are reported** |
| --- | --- | --- | --- | --- |
|  | 1 | (a) Indicate the study’s design with a commonly used term in the title or the abstract (b) Provide in the abstract an informative and balanced summary of what was done and what was found | RECORD 1.1: The type of data used should be specified in the title or abstract. When possible, the name of the databases used should be included.  RECORD 1.2: If applicable, the geographic region and timeframe within which the study took place should be reported in the title or abstract.  RECORD 1.3: If linkage between databases was conducted for the study, this should be clearly stated in the title or abstract. | Abstract methods  Title and abstract  N/A – no bespoke linkage |
| Background rationale | 2 | Explain the scientific background and rationale for the investigation being reported |  | Introduction throughout and supported by full literature review in S1 Text and S1 Table |
| Objectives | 3 | State specific objectives, including any pre-specified hypotheses |  | Introduction, paragraph 5 and methods paragraph 2. |
| Study Design | 4 | Present key elements of study design early in the paper |  | Methods, paragraph 1 and 4 |
| Setting | 5 | Describe the setting, locations, and relevant dates, including periods of recruitment, exposure, follow-up, and data collection |  | Methods, paragraph 1 |
| Participants | 6 | *(a) Cohort study* - Give the eligibility criteria, and the sources and methods of selection of participants. Describe methods of follow-up  *Case-control study* - Give the eligibility criteria, and the sources and methods of case ascertainment and control selection. Give the rationale for the choice of cases and controls  *Cross-sectional study* - Give the eligibility criteria, and the sources and methods of selection of participants  *(b) Cohort study* - For matched studies, give matching criteria and number of exposed and unexposed  *Case-control study* - For matched studies, give matching criteria and the number of controls per case | RECORD 6.1: The methods of study population selection (such as codes or algorithms used to identify subjects) should be listed in detail. If this is not possible, an explanation should be provided.  RECORD 6.2: Any validation studies of the codes or algorithms used to select the population should be referenced. If validation was conducted for this study and not published elsewhere, detailed methods and results should be provided.  RECORD 6.3: If the study involved linkage of databases, consider use of a flow diagram or other graphical display to demonstrate the data linkage process, including the number of individuals with linked data at each stage. | Methods , paragraph 4, 5 and 6, Fig 1 and S2 Table  Validated code lists used, details provided in S2 Table and referenced to HDRUK Phenotype library.  Matching described in Methods, paragraph 6 and 7 |
| Variables | 7 | Clearly define all outcomes, exposures, predictors, potential confounders, and effect modifiers. Give diagnostic criteria, if applicable. | RECORD 7.1: A complete list of codes and algorithms used to classify exposures, outcomes, confounders, and effect modifiers should be provided. If these cannot be reported, an explanation should be provided. | Full list provided in S2 Table. |
| Data sources/ measurement | 8 | For each variable of interest, give sources of data and details of methods of assessment (measurement).  Describe comparability of assessment methods if there is more than one group |  | Variables include outcome measures (defined according to ICD-10 codes as per S2 Table) and demographic variables described in Methods, Statistical Analyses section, paragraph 1 |
| Bias | 9 | Describe any efforts to address potential sources of bias |  | Methods detail adjustment for confounders (Statistical analyses paragraph 2) to minimise bias, risk-set matching procedure (methods paragraph 6) details minimising immortal time bias. Competing risk bias reduced using competing risk analyses (statistical analyses paragraph 3) |
| Study size | 10 | Explain how the study size was arrived at |  | Study size/cohort derivation provided in Fig 1. |
| Quantitative variables | 11 | Explain how quantitative variables were handled in the analyses. If applicable, describe which groupings were chosen, and why |  | Adjustments used in continuous format when adjusting for confounders, and stratified into groups for display of absolute risks by detailed demographics (Fig 4). |
| Statistical methods | 12 | (a) Describe all statistical methods, including those used to control for confounding  (b) Describe any methods used to examine subgroups and interactions  (c) Explain how missing data were addressed  (d) *Cohort study* - If applicable, explain how loss to follow-up was addressed  *Case-control study* - If applicable, explain how matching of cases and controls was addressed  *Cross-sectional study* - If applicable, describe analytical methods taking account of sampling strategy  (e) Describe any sensitivity analyses |  | Statistical analyses, paragraphs 1-4  Risk charts (Fig 4) show stratified data by key demographic groups allowing for assessment of interaction between sociodemographic factors.   Missing data summarised and complete case analyses justified, statistical analyses, paragraph 5.   Detailed methods of risk-set matching procedure provided (methods, paragraph 6 and 7).   Sensitivity analyses conducted and details of methods described in S2 Text. |
| Data access and cleaning methods |  | .. | RECORD 12.1: Authors should describe the extent to which the investigators had access to the database population used to create the study population.  RECORD 12.2: Authors should provide information on the data cleaning methods used in the study. | Contributor statement includes all details regarding data access (Contributorship Statement Section )  Data cleaning steps provided in Fig 1 and S3 Text. |
| Linkage |  | .. | RECORD 12.3: State whether the study included person-level, institutional-level, or other data linkage across two or more databases. The methods of linkage and methods of linkage quality evaluation should be provided. | Individual level HES routinely linked via NHS number to ONS all-cause mortality data – linkage performed by NHS Digital (Methods, paragraph 3). |
| **Results** | | | | |
| Participants | 13 | (a) Report the numbers of individuals at each stage of the study (*e.g.*, numbers potentially eligible, examined for eligibility, confirmed eligible, included in the study, completing follow-up, and analysed)  (b) Give reasons for non-participation at each stage.  (c) Consider use of a flow diagram | RECORD 13.1: Describe in detail the selection of the persons included in the study (*i.e.,* study population selection) including filtering based on data quality, data availability and linkage. The selection of included persons can be described in the text and/or by means of the study flow diagram. | Fig 1 details cohort selection and the filtering process in a flow diagram. |
| Descriptive data | 14 | (a) Give characteristics of study participants (*e.g.*, demographic, clinical, social) and information on exposures and potential confounders  (b) Indicate the number of participants with missing data for each variable of interest  (c) *Cohort study* - summarise follow-up time (*e.g.*, average and total amount) |  | Table 1 provides cohort characteristics.  Missing data described in footer of Table 1. |
| Outcome data | 15 | *Cohort study* - Report numbers of outcome events or summary measures over time  *Case-control study* - Report numbers in each exposure category, or summary measures of exposure  *Cross-sectional study* - Report numbers of outcome events or summary measures |  | Results , paragraph 3 and Fig 2. |
| Main results | 16 | (a) Give unadjusted estimates and, if applicable, confounder-adjusted estimates and their precision (e.g., 95% confidence interval). Make clear which confounders were adjusted for and why they were included  (b) Report category boundaries when continuous variables were categorized  (c) If relevant, consider translating estimates of relative risk into absolute risk for a meaningful time period |  | All results are clearly labelled including crude (unadjusted) or confounder adjustment (Results paragraph 3)– specifying which confounders were included in manuscript text (statistical analyses paragraph 2) as well as Fig 3 and 4 footnotes. 95% CIs reported throughout results and figures.  Risk charts (Fig 4) show measures of absolute risk at specific time points, and relative risk plotted against continuous time (Fig 3A; Fig 3B) |
| Other analyses | 17 | Report other analyses done—e.g., analyses of subgroups and interactions, and sensitivity analyses |  | Sensitivity analyses reported in S2 Text, S1 Figure, S2 Figure, S3 Figure, S5 Figure, S6 Table, S8 Table. |
|  | | | | |
| Key results | 18 | Summarise key results with reference to study objectives |  | Discussion Paragraph 1 |
| Limitations | 19 | Discuss limitations of the study, taking into account sources of potential bias or imprecision. Discuss both direction and magnitude of any potential bias | RECORD 19.1: Discuss the implications of using data that were not created or collected to answer the specific research question(s). Include discussion of misclassification bias, unmeasured confounding, missing data, and changing eligibility over time, as they pertain to the study being reported. | Detailed study limitations in discussion paragraph 6 and 7 |
| Interpretation | 20 | Give a cautious overall interpretation of results considering objectives, limitations, multiplicity of analyses, results from similar studies, and other relevant evidence |  | Overall conclusion - Discussion paragraph 12. |
| Generalisability | 21 | Discuss the generalisability (external validity) of the study results |  | Discussion paragraph 6 and 7 |
| **Other Information** | | | | |
| Funding | 22 | Give the source of funding and the role of the funders for the present study and, if applicable, for the original study on which the present article is based |  | Funding source and role of funders acknowledged in “ Role of the Funding Source” section. |
| Accessibility of protocol, raw data, and programming code |  | .. | RECORD 22.1: Authors should provide information on how to access any supplemental information such as the study protocol, raw data, or programming code. | Data Sharing Statement has been provided – and full list of disease coding provided S2 Table. |
