# Supplementary material for: Health outcomes after myocardial infarction: A population study of 56 million people in England
Source: PLoS Med. 2024 Feb 15;21(2):e1004343. doi: 10.1371/journal.pmed.1004343 (PMC10868847; doi:10.1371/journal.pmed.1004343)
Supplement: S1 Text — (DOCX) [file pmed.1004343.s003.docx]

We searched the Ovid Medline database (1946 to October 2023) and Embase (1996 to October 2023) using medical Subject Heading (MESH) terms “myocardial infarction” or “acute coronary syndrome” combined with outcome terms for “hospitalisation”, “events”, “multimorbidity”, “comorbidity” or “chronic conditions”, with adjacency searching for “post”, “after” or “following” and “incidence”, “prevalence” or “rate”. No language restrictions were applied. We additionally searched the grey literature through citations in peer-reviewed literature and google scholar. Our review focused specifically on studies reporting incidence of new onset disease following myocardial infarction (MI). Studies which focused on fatal outcomes only, studies which investigated the determinants of new onset disease following MI without providing data of the incidence of outcomes directly, and studies which were only published in abstract form, were excluded.

Our search identified 62 studies for which we extracted information of the study design, the outcomes investigated, the reported incidence of post MI health outcomes and the methodology used for the calculation of incidence (S1 Table).

The largest study to date focussed exclusively on all-cause mortality, all-cause re-admission, and recurrent MI up to one year following MI (4.3 million patients, US)[1]. The majority of studies focussed solely on short term incidence of recurrent MI, stroke or bleeding (n=24)[2-16]. Longer term outcomes reported in other studies include post MI incidence of atrial fibrillation[17-19], depression[20-22], heart failure[23-26], cancer[27], and dementia[28]. However, there was considerable variation in the robustness of reported incidence with some providing only unadjusted crude estimates confounded by sociodemographic factors, pre-existing disease and differential exposure times, most unable to quantify the impact of MI on subsequent health outcomes over and above what is expected during the normal life course due to lack of control populations, none reporting absolute incidence by detailed age, sex and deprivation specific demographic groups. There were no nationally representative studies of the risk of new onset peripheral vascular disease, chronic renal failure or diabetes for survivors of MI and data for post MI incidence of depression were limited to studies of fewer than 300 individuals.

None of the identified studies report adjusted cumulative incidence functions accounting for confounding, as well as censoring and competing risks of death (some did one or the other), in order to collectively and robustly quantify the absolute risk of major health outcomes over continuous time. In particular, there have been no studies that systematically describe post MI health outcomes by detailed age, sex and deprivation groups.

**References**

1. Krumholz HM, Normand S-LT, Wang Y. Twenty-year trends in outcomes for older adults with acute myocardial infarction in the United States. JAMA Netw Open. 2019;2(3):e191938-e.

2. Rapsomaniki E, Thuresson M, Yang E, Blin P, Hunt P, Chung S-C, et al. Using big data from health records from four countries to evaluate chronic disease outcomes: a study in 114 364 survivors of myocardial infarction. Eur Heart J Qual Care Clin Outcomes. 2016;Advanced Online:qcw004.

3. Varenhorst C, Hasvold P, Johansson S, Janzon M, Albertsson P, Leosdottir M, et al. Culprit and nonculprit recurrent ischemic events in patients with myocardial infarction: Data from SWEDEHEART (Swedish Web System for Enhancement and Development of Evidence-Based Care in Heart Disease Evaluated According to Recommended Therapies). J Am Heart Assoc. 2018;7 (1)(e007174). PubMed PMID: 620227930.

4. Jernberg T, Hasvold P, Henriksson M, Hjelm H, Thuresson M, Janzon M. Cardiovascular risk in post-myocardial infarction patients: nationwide real world data demonstrate the importance of a long-term perspective. Eur Heart J. 2015;36(19):1163-70.

5. Yang E, Stokes M, Johansson S, Mellstrom C, Magnuson E, Cohen DJ, et al. Clinical and economic outcomes among elderly myocardial infarction survivors in the United States. Cardiovasc Ther. 2016;34(6):450-9. PubMed PMID: 27564212.

6. Gouda P, Savu A, Bainey KR, Kaul P, Welsh RC. Long-term risk of death and recurrent cardiovascular events following acute coronary syndromes. PLoS One. 2021;16(7):e0254008.

7. Brinkert M, Southern DA, James MT, Knudtson ML, Anderson TJ, Charbonneau F. Incidence and Prognostic Implications of Late Bleeding After Myocardial Infarction or Unstable Angina According to Treatment Strategy. Can J Cardiol. 2017;33(8):998-1005. PubMed PMID: 28669702.

8. Li S, Peng Y, Wang X, Qian Y, Xiang P, Wade SW, et al. Cardiovascular events and death after myocardial infarction or ischemic stroke in an older Medicare population. Clin Cardiol. 2019;42(3):391-9. PubMed PMID: 30697776.

9. Roe MT, Li S, Thomas L, Wang TY, Alexander KP, Ohman EM, et al. Long-term outcomes after invasive management for older patients with non-ST-segment elevation myocardial infarction. Circ Cardiovasc Qual Outcomes. 2013;6(3):323-32. PubMed PMID: 23652734.

10. Nedkoff L, Atkins E, Knuiman M, Sanfilippo FM, Rankin J, Hung J. Age-specific gender differences in long-term recurrence and mortality following incident myocardial infarction: a population-based study. Heart Lung Circ. 2015;24(5):442-9. PubMed PMID: 25618449.

11. Guimaraes PO, Krishnamoorthy A, Kaltenbach LA, Anstrom KJ, Effron MB, Mark DB, et al. Accuracy of Medical Claims for Identifying Cardiovascular and Bleeding Events After Myocardial Infarction : A Secondary Analysis of the TRANSLATE-ACS Study. JAMA Cardiol. 2017;2(7):750-7. PubMed PMID: 28538984.

12. Brieger D, Pocock SJ, Blankenberg S, Chen JY, Cohen MG, Granger CB, et al. Two-year outcomes among stable high-risk patients following acute MI. Insights from a global registry in 25 countries. Int J Cardiol. 2020. PubMed PMID: 2004905249.

13. Pocock SJ, Brieger D, Gregson J, Chen JY, Cohen MG, Goodman SG, et al. Predicting risk of cardiovascular events 1 to 3 years post-myocardial infarction using a global registry. Clin Cardiol. 2020;43(1):24-32. PubMed PMID: 2003635562.

14. Canivell S, Muller O, Gencer B, Heg D, Klingenberg R, Räber L, et al. Prognosis of cardiovascular and non-cardiovascular multimorbidity after acute coronary syndrome. PloS One. 2018;13(4).

15. Patel A, Goodman SG, Yan AT, Alexander KP, Wong CL, Cheema AN, et al. Frailty and Outcomes After Myocardial Infarction: Insights From the CONCORDANCE Registry. J Am Heart Assoc. 2018;7(18):e009859. PubMed PMID: 30371219.

16. Barr PR, Harrison W, Smyth D, Flynn C, Lee M, Kerr AJ. Myocardial Infarction Without Obstructive Coronary Artery Disease is Not a Benign Condition (ANZACS-QI 10). Heart Lung Circ. 2018;27(2):165-74. PubMed PMID: 28408093.

17. Kulik A, Singh JP, Levin R, Avorn J, Choudhry NK. Association between statin use and the incidence of atrial fibrillation following hospitalization for coronary artery disease. Am J Cardiol. 2010;105(12):1655-60. PubMed PMID: 20538110.

18. Singh JP, Kulik A, Levin R, Ellinor PT, Ruskin J, Avorn J, et al. Renin-angiotensin-system modulators and the incidence of atrial fibrillation following hospitalization for coronary artery disease. Europace. 2012;14(9):1287-93. PubMed PMID: 22539600.

19. Jabre P, Jouven X, Adnet F, Thabut G, Bielinski SJ, Weston SA, et al. Atrial fibrillation and death after myocardial infarction: a community study. Circulation. 2011;123(19):2094-100. PubMed PMID: 21536994.

20. Kala P, Hudakova N, Jurajda M, Kasparek T, Ustohal L, Parenica J, et al. Depression and anxiety after acute myocardial infarction treated by primary PCI. PLoS One. 2016;11(4):e0152367.

21. Liang JJ, Tweet MS, Hayes SE, Gulati R, Hayes SN. Prevalence and predictors of depression and anxiety among survivors of myocardial infarction due to spontaneous coronary artery dissection. J Cardiopulm Rehabil Prev. 2014;34(2):138-42. PubMed PMID: 24280906.

22. Lane D, Carroll D, Ring C, Beevers DG, Lip GYH. The prevalence and persistence of depression and anxiety following myocardial infarction. Br J Health Psychol. 2002;7(1):11-21. PubMed PMID: 34174896.

23. Marchioli R, Levantesi G, Macchia A, Marfisi RM, Nicolosi GL, Tavazzi L, et al. Vitamin E increases the risk of developing heart failure after myocardial infarction: Results from the GISSI-Prevenzione trial. J Cardiovasc Med. 2006;7(5):347-50. PubMed PMID: 16645413.

24. Ezekowitz JA, Kaul P, Bakal JA, Armstrong PW, Welsh RC, McAlister FA. Declining in-hospital mortality and increasing heart failure incidence in elderly patients with first myocardial infarction. J Am Coll Cardiol. 2009;53(1):13-20. PubMed PMID: 19118718.

25. Gerber Y, Weston SA, Enriquez-Sarano M, Berardi C, Chamberlain AM, Manemann SM, et al. Mortality Associated With Heart Failure After Myocardial Infarction: A Contemporary Community Perspective. Circ Heart Fail. 2016;9(1):e002460. PubMed PMID: 26699392.

26. Jhaveri RR, Reynolds HR, Katz SD, Jeger R, Zinka E, Forman SA, et al. Heart failure in post-MI patients with persistent IRA occlusion: prevalence, risk factors, and the long-term effect of PCI in the Occluded Artery Trial (OAT). J Card Fail. 2012;18(11):813-21. PubMed PMID: 23141853.

27. Malmborg M, Christiansen CB, Schmiegelow MD, Torp-Pedersen C, Gislason G, Schou M. Incidence of new onset cancer in patients with a myocardial infarction–a nationwide cohort study. BMC Cardiovasc Disord. 2018;18(1):1-9.

28. Sundbøll J, Horváth-Puhó E, Adelborg K, Schmidt M, Pedersen L, Bøtker HE, et al. Higher risk of vascular dementia in myocardial infarction survivors. Circulation. 2018;137(6):567-77.
