# Supplementary material for: Health outcomes after myocardial infarction: A population study of 56 million people in England
Source: PLoS Med. 2024 Feb 15;21(2):e1004343. doi: 10.1371/journal.pmed.1004343 (PMC10868847; doi:10.1371/journal.pmed.1004343)
Supplement: S2 Text — (DOCX) [file pmed.1004343.s004.docx]

We conducted sensitivity analyses in which we restrict our follow up period to two or more months following myocardial infarction (MI). This decision was made on the basis that we observed a high number of events at the same time as, or shortly after, study entry. Whilst this in part reflects the nature of our follow-up period beginning at the point of first hospitalisation; the high number of early events may also likely reflect 1) conditions which are clinically expected to occur shortly after MI or first hospitalisation due to overall declining health, 2) underlying and pre-existing conditions which are diagnosed only upon hospital admission due to investigations undertaken whilst in hospital or 3) existing conditions diagnosed outside of secondary care with a first recording in hospital episode statistics (HES) at admission. The sensitivity analyses are included to mitigate potential bias against events occurring according to scenario 3, whilst acknowledging this may also undercount the occurrence of some events representing true index disease represented by scenarios 1 and 2.
