# Supplementary material for: Health outcomes after myocardial infarction: A population study of 56 million people in England
Source: PLoS Med. 2024 Feb 15;21(2):e1004343. doi: 10.1371/journal.pmed.1004343 (PMC10868847; doi:10.1371/journal.pmed.1004343)
Supplement: S3 Text — (DOCX) [file pmed.1004343.s005.docx]

A thorough data quality assessment was conducted prior to analyses and details published[1]. Specifically for this study data were cleaned by removing duplicate records and removing records which were deemed unusable due to very poor data quality (Manuscript Figure 1). Duplicate HES episodes which contain the same data across HES fields including admission start and end dates, episode start and end dates, primary and secondary diagnoses codes and procedure codes are administrative duplications where incorrect or new entries have been created. Whilst there were a large number of duplicate episodes, we do not anticipate that this presents any bias in the results given the only impact of the exclusion of duplicate episodes on the analyses is to identify the appropriate denominator of total number of episodes per cohort as described in Table 1. Entries of hospitalisation codes are only counted once at first occurrence – therefore our analyses is robust to the inclusion or exclusion of such duplicates, and no individual patients were removed as a result of excluding duplicate hospitalisation records. Records with missing or conflicting information across core data fields including episode start and end dates, spell begin and end indicators and episode order were deemed as having too poor a quality to be included in analyses as missing or conflicting information across these core fields leads to questions about the reliability of the entire record. Moreover, such records are likely to have been introduced to the data due to data entry errors or computer glitches and as such were removed prior to analyses. Furthermore, records with episode start dates prior to January 2008 were removed as these were incorrectly provided in the data extract. Finally, records for patients who had died but without any available information regarding their survival time were also excluded from analyses. The nature of these exclusions, their small relative quantities and the scale of the overall remaining analytical cohort size precluded the need for more sophisticated missing data methods.

**Reference**

1. Ruddle R, Hall M. Using Miniature Visualizations of Descriptive Statistics to Investigate the Quality of Electronic Health Records. HEALTHINF. 2019;5:pp. 230-8.
