# Supplementary material for: Health outcomes after myocardial infarction: A population study of 56 million people in England
Source: PLoS Med. 2024 Feb 15;21(2):e1004343. doi: 10.1371/journal.pmed.1004343 (PMC10868847; doi:10.1371/journal.pmed.1004343)
Supplement: S1 Table — ACEi/ARB, angiotensin-converting enzyme inhibitors and angiotensin II receptor blockers; ACS, acute coronary syndromes; AF, atrial fibrillation; CAD, coronary artery disease; CIF, cumulative incidence function; COPD, coronary obstructive pulmonary disease; CPRD, Clinical Practice Research Database; EHR, electronic healthcare record; HES, Hospital Episode Statistics; HF, heart failure; HFrEF, heart failure with reduced ejection fraction; HR, hazard ratio; IRR, incidence rate ratio; IQR, interquartile range; KM, Kaplan–Meier; MI, myocardial infarction; MINAP, Myocardial Ischaemia National Audit Project; NSETMI, non ST-elevation myocardial infarction; PCI, percutaneous coronary intervention; PH, proportional hazards; PPCI, primary percutaneous coronary intervention; PTSD, posttraumatic stress disorder; SCAD, spontaneous coronary artery dissection; SD, standard deviation; STEMI, ST-elevation myocardial infarction; USA, United States of America; vs., versus. (DOCX) [file pmed.1004343.s006.docx]

| **Author  Study design** | **Study cohort (N)** | **Clinical cohort** | **Years of study** | **Follow up period** | **Post MI outcomes studied** | **Cohort details and baseline characteristics** | **Post MI disease incidence results** | **Post MI disease incidence methods** |
| --- | --- | --- | --- | --- | --- | --- | --- | --- |
| Schmitt, 2021[1]  Nationwide German inpatient study | 3,307,703 | MI | 2005-2016 | In-hospital | Multiple hospitalisation outcomes (In-hospital death, recurrent MI, pneumonia, deep venous thrombosis, pulmonary embolism, acute kidney injury, stroke, intracerebral bleeding, gastrointestinal bleeding, haemopericardium, and blood component transfusion). | 37.6% female, 56.8% aged ≥ 70 years. 30.5% of MI cases also had diabetes mellitus. | Post MI outcomes for people with MI and diabetes vs MI without diabetes:  In-hospital mortality 13.2% vs. 12.1% Recurrent MI 0.8% vs. 0.6% Pneumonia 14.9% vs. 10.2%  Acute kidney injury 8.6% vs. 5.2% Stroke 3.4% vs 2.7% Need for blood constituent transfusions 15.7% vs 11.4% | Raw case numbers/percentages for people with MI with and without diabetes. Subsequent logistic regression used to model impact of diabetes mellitus versus no diabetes mellitus on outcomes. |
| Krumholz, 2019[2]  Medicare administrative data; 5680 hospitals in the US | 4.3 million | MI | 1995-2014 | 1 year | Cardiovascular outcomes (30-day all-cause mortality, 30-day all cause readmission, 1 year recurrent MI) | Non-index MI patients > 65 years of age. Mean age 78.2 years (SD 8.0), 50.4% male. Medical history: previous MI 4.8%, 14.1% HF, 4.1% Unstable angina, 57.3% hypertension, 2% stroke, 4.4% cerebrovascular disease, 7.3% renal failure, 21.8% COPD, 13% pneumonia, 8.4% dementia, 6.1% peripheral vascular disease, 5.5% metastatic cancer, 31.2% diabetes, 4.5% depression | Crude rates at end of study (2014) were 30-day mortality 12.4%; 30-day all-cause readmissions 15.3% and 1-year recurrent MI 5.1% | Raw case numbers/percentages for post MI outcomes. Hierarchical logistic regression use to determine temporal trends for 30 day and 1 year outcomes |
| Rapsomaniki, 2016[3]  International comparison of EHR in England, Sweden, USA and France | 114,364 | MI | 2002-2011 | 3 years | Cardiovascular outcomes (All cause death; a composite of myocardial infarction (MI), stroke, and all-cause death; and hospitalized bleeding). | Index acute MI patients >65 years having survived >one-year post index MI. Mean age ranged from 77.5 years in England to 78.6 years in the United States of America (USA). Co-morbid population with up to 26.6% diabetes, 45% heart failure, 25.4% atrial fibrillation, 13% stroke. | 3-year crude cumulative risks of all-cause death ranged from 19.6% [England] to 30.2% [USA]); the composite of MI, stroke, or death ranged from 26.0% (France) to 36.2% (USA)]; and hospitalized bleeding from 3.1% (France) to 5.3% (USA). | [Predicted risks adjusted to the average characteristics of the Swedish patients based on incrementally adjusted Cox models](https://www.ncbi.nlm.nih.gov/pmc/articles/PMC5815620/#sup1) |
| Varenhorst, 2018[4]  National registry (SWEDEHEART) | 108,615 | MI | 2006-2014 | 3.2 years | Cardiovascular outcomes (recurrent MI) | Index MI patients, 42.1% prior hypertension, 14.3% prior diabetes, 2.6% previous PCI, 9.5% previous stroke. | Recurrent hospitalization for MI occurred in 11,117 patients (10.2%). | Non-parametric KM cumulative incidence functions for recurrent MI. Competing risk in sensitivity analyses using Fine and Gray models. |
| Jernberg, 2015[5] Retrospective cohort study, Sweden | 97,254 | MI | 2006-2011 | 1 year | Cardiovascular outcomes (non-fatal MI, non-fatal stroke of cardiovascular death) | Non-index MI patients, 72.4 years of age, 61.6% male. Medical history of cohort: 26.3% heart failure, 10.4% previous MI, 5% unstable angina pectoris, 10.8% stroke, 1.2% renal dysfunction, 23.3% diabetes, 5.8% major bleeding, 12.8% cancer | Composite endpoint risk was 18.3% during the first 365 days post-index MI. Prior MI [HR 1.44, 95% CI 1.40,1.49], stroke [HR 1.49, 95% CI 1.44,1.54], diabetes [HR 1.37, 95% CI 1.34,1.40], heart failure [HR 1.57, [95% CI 1.53,1.62] and no index MI revascularisation [HR 1.88, 95% CI 1.83,1.93] conferred increased risk of the composite endpoint. | Unadjusted cumulative incidence function over time, accounting for competing risk. Confounder adjustment made in analyses of the determinants of outcomes. |
| Yang, 2016[6]  5% random sample of Medicare claims data, US | 53,929 | MI | 2002-2009 | 3 years | Cardiovascular outcomes (mortality, re-hospitalisation for stroke, major bleeding or subsequent MI) | Patients were ≥65 years old and survived ≥1 year without recurrent MI after MI hospitalization. Mean age 78.6 years; 50.8% male. Co-morbid at baseline with 48% diabetes, history of MI (12%), atrial fibrillation (38.4%), heart failure (64%), stroke (18%). | Post MI incidence/100 person-years over the full follow-up period is 2.29 for subsequent MI; 1.41 for stroke, 15.96 for the composite of MI, stroke or all-cause death; 2.32 for hospitalised major bleeding. Post MI healthcare resource utilization includes 4.47 days in hospital, 6.20 outpatient visits, 0.1 emergency visits and up to 17 GP visits. Post MI healthcare costs amount to $13111 per person per year. | Unadjusted incidence per 100 person years of follow up. Cox proportional hazard models to evaluate the impact of baseline medical history on incidence of each post MI outcome |
| Gouda, 2021[7]  Retrospective population study of hospitalisations in Alberta, Canada. | 31,056 | ACS | 2010-2016 |  | Cardiovascular outcomes (all-cause mortality, re-hospitalisation for stroke, re-hospitalisation for MI). | Mean age was 66.2 years (SD 13.8 years]. 30.7% (n = 9529) were female, 49.5% NSTEMI. 11.1 had previous history of heart failure, 63.0% hypertension, 28.3% diabetes, 2.4% cerebrovascular disease, 2.9% peripheral vascular disease and 4.0% renal disease. | Post NSTEMI rate of composite of death, recurrent MI and stroke: 3.58 per 10,000 person-years; post STEMI rate of composite of death, recurrent MI and stroke: 2.41 per 10,000 person-years. | Unadjusted rates per 10,000 person-years provided in text, and unadjusted Kaplan-Meier estimates for the cumulative incidence of post ACS outcomes provided in graphical form only. Adjusted Cox proportional hazards models and Fine and Gray models including competing risk of death were used to assess association between co-morbidities and outcomes. |
| Brinkert, 2017[8]  Consecutive patient cohort, Alberta, Canada | 31,941 | ACS | 2005-2011 | 1 year | Cardiovascular outcomes (re-admission with bleeding) | ACS Patients, mean age 71.8 years (SD 14.7); 56.4% Male. No overall cohort statistics provided on co-morbidities - but across subgroups, this is a highly co-morbid population including congestive heart failure, peripheral vascular disease, COPD, cerebrovascular disease, rheumatic disease, liver disease and cancer. | Overall incidence of readmission for bleeding: 3.8% for medically treated patients, 2.8% for patients who underwent angiography alone, 2.6% for patients who underwent CABG, 1.8% for patient who underwent PCI. 52% gastrointestinal bleeding, 7.8% intracranial bleeding. | Adjusted logistic regression models for 1 year outcomes |
| Li, 2019[9]  20% random sample of Medicare patients in US | 26,548 | MI | 2008 and 2012 | 6 years | Cardiovascular outcomes (recurrent MI, unstable angina, ischemic stroke, transient ischaemic attack, CABG/PCI) | Non-index MI patients; mean age 80.1 years, 42% male; highly co-morbid with 92.4% hypertension, 22.3% cerebrovascular disease, 33.5% prior MI, 6.8% ischemic stroke | In older patients who survived an event of MI or ischemic stroke, 7.2% had a recurrent MI in the first year; 32% died. Accounting for multiple recurrent events, the event rates per 100 patient-years were 11.6. | Unadjusted non-parametric cumulative incidence functions accounting for competing risk of death for post MI outcomes |
| Roe, 2013[10]  CRUSADE registry data | 19,336 | NSTEMI and matched longitudinal administrative data | 2003-2006 | 3.2 years | Cardiovascular outcomes (all-cause mortality, re-admission for MI or stroke, repeat revascularisation) | Overall NSTEMI cohort included people aged >65 years; median age 75 years; 41.6% female and 87.4% White ethnicity. | Unadjusted cumulative incidence of the composite of death, readmission for MI, or readmission for stroke at 5 years was 62.4%, 44.9%, and 33.0% for medical management, PCI, and coronary artery bypass grafting, respectively. | Unadjusted cumulative incidence for all outcomes, accounting for competing risk of death |
| Nedkoff, 2015[11]  Linked administrative data in Western Australia | 12,420 | MI | 2003-2009 | 8 years | Cardiovascular outcomes (recurrent MI, cardiovascular and all-cause mortality) | Index MI patients surviving at least 30 days post MI were included. Mean age 64.5 years, 71.2% male. Pre-existing co-morbidities included up to 14% coronary heart disease, 27.7% diabetes, 12.4% chronic kidney disease, 63% hypertension, 19.2% heart failure, 4.6% stroke, 16.3% atrial fibrillation | 8-year post MI risk of recurrent MI was 11.6% [95% CI 10.6%,12.7%] and 14.9% [95% CI 12.6%,17.2%) for men and women aged 35-84 years. All-cause mortality risk was 21.2% [95% CI 19.8%,22.6%] and 34.1 [95% CI 31.4%,36.8%) for men and women respectively aged 35-84 years. | Unadjusted non-parametric Kaplan-Meier estimates for post MI outcomes |
| Guimaraes, 2017[12]  Re-hospitalizations at 233 US hospitals - TRANSLATE-ACS | 12,365 | MI | 2010-2012 | 1 year | Cardiovascular outcomes (re-hospitalisation for recurrent MI, stroke or bleeding) | 8,890 (71.9%) were men and mean age was 60 years [SD 11.6] years. | The cumulative 1-year incidence of events identified by medical claims was 4.3% for MI, 0.9% for stroke, and 5.0% for bleeding. Incidence rates based on physician adjudication were 4.7% for MI, 0.9% for stroke, and 5.4% for bleeding. The post MI incidence of death/re-MI or stroke was 6 in medical claims data, and 7.5 in the physician adjudicated data. | Unadjusted non-parametric Kaplan-Meier estimates for post MI outcomes |
| Brieger, 2020[13]  Multicentre, international study of 25 countries (TIGRIS registry) | 9,027 | MI | 2013-2014 | 2 years | Cardiovascular outcomes (MI, unstable angina requiring revascularisation, stroke and all-cause mortality) | Patients with stable CAD, aged>50 years with history of MI 1-3 years prior to study included. 30.5% history of diabetes, 7.7% chronic kidney disease. Patients with serious/severe co-morbidities that could limit life expectancy to less than 1 year were excluded. | The primary outcome occurred in 621 (7.0%), all-cause mortality in 295 (3.3%), and bleeding in 109 (1.2%) patients. IRR per 100 person-years: chronic kidney disease 2.06 [95% CI 1.66,2.55],  second prior MI IRR 1.71 [95% CI 1.38,2.10],  diabetes mellitus IRR 1.63 [95% 1.39,1.92]  multi-vessel disease IRR 1.24 [95% CI 1.05,1.48]. Risk of bleeding events was greater in older patients (vs <65 years) 65-74 years IRR 2.68 [95% CI 1.53,4.70], >=75 years IRR 4.62 [95% CI 2.57,8.28], and those with chronic kidney disease IRR 1.99 [95% CI 1.18, 3.35]. | Incidence rates per 100 person-years, cumulative incidence functions over time and multivariate Poisson regression for rate ratios (overall without age, sex, deprivation stratification). |
| Pocock, 2020[14]  Multicentre, international study of 25 countries (TIGRIS registry) | 9,027 | MI | 2013-2015 | 2 years | Cardiovascular outcomes (MI, unstable angina requiring revascularisation, stroke and all-cause mortality) | Patients with stable CAD, aged>50 years with history of MI 1-3 years prior to study included. 30.5% history of diabetes, 7.7% chronic kidney disease. Patients with serious/severe co-morbidities that could limit life expectancy to less than 1 year were excluded. | The primary outcome occurred in 621 (6.9%) from 9027 patients over 2 years, with all-cause death in 295 (3.3%), MI in 195 (2.2%), unstable angina requiring revascularization in 103 (1.1%), and stroke in 58 (0.6%) patients. | Unadjusted incidence rate per 100 patient‐years for post MI outcomes. |
| Grodzinsky, 2015[15]  Multicentre cohort 31 hospitals in the US | 5,539 | MI | 2003-2008 | 1 year | Cardiovascular outcomes (angina); self-reported | Non index MI patients. Overall mean age 60 years, 68% male. Co-morbid with diabetes (28.5%) and hypertension (63.4%). | Angina affected 1 in 4 patients after MI. The risk of post MI angina was similar in patients without vs. with obstructive CAD IRR = 0.89, [95% CI 0.77,1.02]. Among patients without obstructive CAD, depression and self-reported avoidance of care due to cost were independently associated with angina (IRR = 1.28, [95% CI 1.17,1.41]; IRR = 1.34, [95% CI 1.02,1.74]). | Raw case numbers/percentage for post MI incidence of angina. Poisson regression modelling to determine factors associated with post MI angina. |
| Canivell, 2018[16]  Multicentre prospective cohort study, Switzerland | 5,635 | ACS | 2009-2014 | 1 year | Cardiovascular outcomes (cardiovascular mortality, non-fatal MI or stroke) | Non-index ACS patients (unstable angina and MI). 33% of ACS cohort has cardiovascular multimorbidity at baseline, 1% had non-cardiovascular multimorbidity and 1% had both cardiovascular and non-cardiovascular multimorbidity. Exclusion criteria were the presence of severe physical disability, inability to give consent due to dementia, and life expectancy of less than one year for non-cardiac reasons. | Risk of recurrent cardiovascular outcomes in patients with cardiovascular multimorbidity (HR 2.05, 95% CI: 1.54,2.73), with non-cardiovascular multimorbidity (HR 2.57, 95% CI: 1.04,6.35) and with cardiovascular and non-cardiovascular multimorbidity (HR of 5.19, 95% CI: 2.79,9.64) vs. without multimorbidity. | Risk of recurrent events modelled using Cox proportional hazards modelling. No competing risk of death |
| Patel, 2018[17]  CONCORDANCE Registry data; 41 hospitals, Australia | 3,944 | MI Genetics compared with 15,400 exome sequences from unrelated individuals | 2009-2016 | 6 months | Cardiovascular outcomes (cardiovascular related re-hospitalisation, all-cause mortality and cardiac mortality) | All patients > 65 years old with STEMI or NSTEMI. Overall average age 75 years, ~69% male | 6 month post MI all-cause mortality STEMI 3.3% non-frail, 12.8% frail; NSTEMI 3.9% non-frail, 12.6% frail; 6 months post discharge cardiac re- hospitalisation for STEMI 19.5% non-frail, 29.1% frail and for NSTEMI 20.2% non-frail, 29.4% frail. | Raw case numbers/percentages for post MI outcomes. Hierarchical logistic regression use to determine temporal trends for 30 day and 1 year outcomes |
| Koohi 2021[18]  Prospective population-based cohort study; Iran | 3,699 | CVD | 2002-2005  and  2009-2011 | 10 years | Cardiovascular outcomes (non-fatal myocardial infarction, fatal coronary heart disease, and fatal or non-fatal stroke) | Mean age 53.2 years (SD 9.3); 42.9% male. Co-morbid with 13.2% diabetes, 34.1% hypertension; 12.5% current smokers. | Three trajectories 1) Individuals with stable cardiovascular disease risk scores ranges from 0.02 to 0.03. 2) Individuals with cardiovascular disease risk score increases from 0.09 to 0.17; 3) Individuals whose cardiovascular disease risk score increases over time with a mean risk score range from 0.20 to 0.38. | Distinct baseline to 10-year cardiovascular disease risk trajectories from group-based trajectory models using a censored normal model. Non-continuous follow up data - measured instead at 4 discreet time points over a 10 year period. |
| Barr, 2018[19]  Single centre, consecutive patients in New Zealand | 2,070 and 20,700 matched patients | MI and age and sex matched patients without known CVD | 2007-2012 | 2 years | Cardiovascular outcomes (mortality and non-fatal MI composite) | MI population stratified according to obstructive CAD and non-obstructive CAD. 77.4% Male, mean age 60.7 years (SD 11.9); 27% history of diabetes, 26% history of cardiovascular disease and 4.5% history of congestive heart failure. | The cumulative two-year Kaplan-Maier composite outcome of mortality or non-fatal MI was 14.3% for MI with obstructive CAD, 4.6% for MI without obstructive disease, and 2.2% for patients without prior cardiovascular disease (p<0.001). | Cox PH modelling comparing outcomes between CAD and no-CAD MI patients, and MI vs no-MI patients. No age/sex/deprivation stratification |
| Maddox, 2008[20]  Multicentre cohort study, 19 hospitals in the US | 1,957 | MI | 2003-2004 | 1 year | Cardiovascular outcomes (angina); self-reported | MI patients with angina age 58 years; 63.5% male. MI patients without angina age 61.9 years; 68.6% male. | Overall 389 (19.9%) reported angina 1 year after their index MI hospitalisation | Raw case numbers/percentage for post MI incidence of angina. Poisson regression modelling to determine factors associated with post MI angina. |
| Frasure-Smith, 1999[21]  Prospective cohort study from trial data, with survey follow up data | 896 | MI | 1991-1994 | 18 months | Cardiovascular outcomes (Cardiovascular mortality in patients with and without depression) | Non index MI patients. Patients excluded if they had other life-threatening conditions, cognitively impaired or unable to complete an in-hospital based interview. MI patients had history of hypertension (51.9% females, 27.9% among males), diabetes (23.3% among females, 12.9% among males), prior MI (18.7% females, 25.8% males) | 290 patients (32.3%) had mild to moderate symptoms of depression post MI. Increased depression scores were significantly related to cardiac mortality for both genders [odds ratio 3.29 (1.02-10.59) and 3.05 (1.29-7.17) for women and men respectively. | Raw case numbers/percentage for post MI incidence of depression. Logistic regression modelling for association of depression with mortality in MI patients |
| Rincon, 2019[22]  Prospective study with consecutive patients, Spain | 81 | MI | 2013-2014 | 4.1 years | Cardiovascular outcomes (composite of cardiovascular mortality, recurrent ACS, cardiac re-hospitalisation) | Prospective non-diabetic patients <55 years of age with MI | 24 recurrent cardiovascular disease events in 81 patients post MI. Compared with the low-risk genetic risk tertile, the multivariate-adjusted HR for recurrences was 10.2 (95%CI, 1.1,100.3; P=.04) for the intermediate-risk group and was 20.7 (2.4,181.0; P=.006) for the high-risk group when low-density lipoprotein cholesterol was>=2.8mmol/L (>= 110mg/dL). | Genetic risk score development, Cox proportional hazards modelling |
| Biere, 2015[23]  Single centre, Aarhus University Hospital | 193 | STEMI |  | 1 year | Cardiovascular outcomes (pericardial effusion) | Index STEMI events. 85% Male, with medical history of hypertension (32%), diabetes (15%), active smokers (44%), dyslipidaemia (49%) | Overall, 113 patients (58.5%) had pericardial effusion | Raw case numbers/percentages for post MI outcomes. |
| Jokhadar, 2004[24]  Community medical records Olmsted County, Minnesota | 2,277 | MI | 1979-1998 | 21 years | Cardiovascular outcomes (sudden cardiac death, re-current ischemic events) | Mean age 67 years, 57% men. Co-morbid with diabetes (20%), hypertension (56%) and familiar history of coronary heart disease (21%). | After 3 years, the event-free survival rate was 94% (95% confidence interval: 93, 95) for sudden cardiac death and 56% (95% confidence interval: 54, 58) for recurrent ischemic events. Recurrent MI, unstable angina and sudden cardiac death occurred in 589 (25.9%), 1,029 (45.2%) and 253 (11%) patients respectively. | Raw case numbers/percentages post MI outcomes alongside 3-year event free survival rates. |
| Jons, 2011[25]  Multicentre cohort, 10 Scandinavian centres | 271 | MI with LVEF<40% | 2001-2007 | 2 years | Cardiovascular outcomes and new onset atrial fibrillation | 271 post MI patients with left ventricular ejection fraction <40% and no history of previous AF | Risk of new-onset AF is highest during the first 2 months post MI (16% event rate) and decreases until month 12 post MI, after which the risk for new-onset AF is stable. Risk of major cardiovascular events increased in patients with AF >30 seconds (hazard ratio [95% CI] = 2.73 [1.35,5.50], P = .005), but not in patients with AF <30 seconds (hazard ratio [95% CI] = 1.17 [0.35,3.92], P = .80). More than 90% of all recorded AF events were asymptomatic. | Unadjusted non-parametric KM cumulative incidence rates |
| Levantesi, 2005[26]  Multi-centre open-label clinical study (GISSI-Prevenzione Trial) | 11,323 | MI | 1993-1995 | 3.5 years | Cardiovascular outcomes plus diabetes and heart failure | Diabetes and heart failure outcomes were assessed in patients with no prior evidence of diabetes and heart failure at baseline. | Post MI mortality occurred in 384 (7.4%) of patients, hospitalisation for heart failure occurred in 71 (1.6%) patients and diabetes occurred in 454 (9.6%) of patients | Raw numbers/percentages for post MI outcomes. Cox proportional models to determine impact of variables on outcomes |
| Tran, 2018[27]  Emergency departments or hospitals in Alberta, Canada | 41,210 | MI | 2004-2014 | 2 years | Cumulative composite (resource use) | Patients aged >18 years who were admitted with incident AMI at emergency departments or hospitals in Alberta, Canada. Mean age 66.3 years, 67% male. Co-morbid at baseline with 60% hypertension, 17.5% heart failure, 4.3% dementia, 16.9% COPD. | A patient with AMI visited ambulatory care services 1.2 times and spent 8.6 days in hospital during the first year. During each subsequent year, there were approximately 4 ambulatory care visits for every 100 patients with AMI, and each patient spent 0.2 days in hospital. | Trends of costs over time and costs between MI groups were compared using univariate GLM regression. Count data (i.e., number of hospital days) were compared across MI groups using negative binomial regression |
| Chen, 2015[28]  Multicentre study, 3 Massachusetts hospitals | 4,810 | MI | 2001-2011 | 30 days | Cumulative composite (re-hospitalisation) | MI patients, aged 68.9 years, 58.2% male. No overall study population rates presented of medical history, but of 1370 patients in the latest period, medical history included 13% AF, 19.6% HF, 75.4% Hypertension, 19.9% peripheral vascular disease, 9.8% stroke, 37.4% Diabetes, 17.5% Depression, 22.3% chronic kidney disease. | 30-day re-hospitalization rate post MI was 18.5%.   Proportion of patients who were re-hospitalized was highest (6.6%) during the first week after discharge and continued to decrease with follow up time. Re-hospitalizations during the first week post discharge accounted for 35.6% of all 30-day re-hospitalizations. | Proportion of cases with outcomes out of total, and logistic regression models to model temporal changes in outcomes. |
| Myers, 2014[29]  Multicentre, 8 hospitals, central Israel | 1,151 | MI | 1992-1993 | 13 years | Cumulative composite (frailty) | A cohort of first MI patients aged ≤65 years, excluding patients who were already frail at baseline | During 13 years of follow up - 399 patients (35%) developed frailty. | Post MI outcome summarised according to proportion of patients out of the total cohort. Multivariable logistic regression models were constructed to assess the odds ratios (OR) and 95% confidence intervals (CI) for frailty associated with different sets of covariates. |
| Chamberlain, 2013[30]  Community based cohort; Olmsted County, Minnesota | 1,502 | MI | 2002-2010 | 3.9 years | Cumulative composite (re-hospitalisation in AF vs no AF post MI patients) | Index MI patients. 57.7% were men and the mean age was 67.6 ± 15 years. | 163 (10.9%) developed new-onset AF, 113 (7.5%) developed late-onset AF. | Crude proportion of cases out of total cohort for post MI incidence of AF. Anderson-Gill models, which allows for modelling of multiple outcome events, was used to calculate hazard ratios (HRs) of hospitalizations and emergency department visits for new onset AF |
| Robertson, 2003[31]  Province of Prince Edward Island, Canada | 192 | MI hospitalisations | 1997 | 1 year | Cumulative composite (re-hospitalisation) | Details not provided | The re-admission rate post MI was 57.8% within 1 year. The average length of stay in hospital for MI was 10.3 days, compared to a national average of 6.6 days in hospital. | Unadjusted proportion of cases out of total cohort |
| Kochar, 2022[32] | 1,531,638 | MI | 2001-2012 | 1 year for heart failure incidence | Heart failure, all-cause mortality and composite of all-cause mortality, hospitalisation for MI or hospitalisation for stroke. | Index MI for patients aged 65 years or older. Median age 78 years, 49.7% female, 10.3% non-White race. 64.6% NSTEMI. 56.7% had hypertension, 30.0% diabetes mellitus, 23.6% COPD, 10.8% peripheral arterial disease, 12.8% renal insufficiency, 21.2% Anaemia. | The total rate of new-onset post-MI heart failure within 1 year of MI was 36.0%. | Individuals with heart failure in the year prior to MI were excluded. Individuals who died at index MI admission were excluded.  Cumulative incidence accounting for censoring and competing risk of death – no confounder adjustment. Broad age stratification (<75 years versus ≥ 75 years) conducted. |
| Chen 2013[33]  National sample of Medicare fee-for-service beneficiaries | 2,789,943 | MI | 1998-2011 | 1 year | Heart failure | Index admissions for MI, excluding patients aged <65 years and patients with <1 year of hospitalisation data. Mean age 78.5 years (SD 8.0), 50.2% male. 42% had prior CHF, 27.6% had prior diabetes, 19.5% had prior COPD, 15.7% had prior rheumatic heart disease, 16.1% prior renal failure, 7.4% dementia, 3.6% cancer, 2.1% stroke. | A total of 366,841 patients were hospitalized for HF within a year of discharge from MI. The number of HF hospitalizations after MI declined from 16.1 per 100 patient-years in 1998 to 14.2 per 100 patient-years by 2010. | Incidence rates per person-years of follow up. Poisson models to model incidence over time. |
| Desta 2015[34]  National registry data (SWEDEHEART) | 199,851 | MI | 1996-2008 | In-hospital | Heart failure | First MI in study period (10.6% had previous MI). Of 15,625 patients in final year of study (2008), mean age 70.9 years (SD 12.5), 62.9% male, history of diabetes (22.3%), hypertension (47%), 9.7% congestive heart failure, 9.8% stroke, 9.6% COPD, 5,2% peripheral arterial disease, 2.8% renal failure. | The incidence of HF declined from 46% to 28% between 1996 and 2008. | Raw case numbers/percentages for post MI heart failure. Predictors of post MI HF modelled using logistic regression |
| Gjesing, 2014[35]  Administrative nationwide registers, Denmark | 89,389 | MI without prior heart failure | 1997-2010 | 90 days | Heart failure | Index MI patients without prior HF. HF defined as HF diagnoses or use of loop diuretic. Co-morbidities at baseline - 10.3% chronic ischemic heart disease, 8% chronic vascular disease, 2.8% AF, 7.3% cancer, 8% diabetes. | The incidence of HF (defined as HF diagnosis or incident use of loop diuretics) decreased from 23.6% in 1997–98 to 19.6% in 2009–10. | Cox proportional hazards regression models were performed to obtain hazard ratios (HR) for the risk of developing HF within 90 days after discharge from index MI and for the risk of mortality, and subsequently competing risk analyses. |
| Gho, 2018[36] Linked EHR data across CPRD, MINAP and HES (CALIBER) | 24,479 | MI | 1998-2010 | 3.7 years | Heart failure | Index MI patients, excluding those with history of HF, and those with fatal index MI. 15969 (65.2% were male). 12258 (50.1%) had hypertension, and 3014 (12.3%) had a history of diabetes at baseline. 11.2% previous PCI, 11.3% history of non-metastatic cancer, 2% previous stroke, 4.7% previous transient ischaemic attack. | Post MI incidence of HF 63.8/1000 person-years over 3.7 years of follow up. In England, one in four survivors of a first MI develop HF within 4 years. This contemporary study demonstrates that patients with MI are at considerable risk of HF. | Non-parametric cumulative incidence functions, stratified by <50, 50-65 and >=65 years of age and type of MI. Cox PH modelling of association of baseline variables with onset of HF. |
| Hung 2013[37]  Western Australia | 20,812 | MI | 1996-2007 | 90 days | Heart failure | Index MI for those aged 40 to 84 years without prior HF | Concurrent HF comprised 75% of incident HF cases. The prevalence of HF after MI declined from 28.1% to 16.5% between 1998 and 2007. | Post MI risk of disease was summarised using raw numbers/percentages. Subsequently logistic regression and Cox models were used to determine the association of a range of risk-factors on the development of new onset heart failure. |
| Marchioli, 2006[38]  Post-hoc analyses of trial data | 8,415 | MI without prior heart failure | 1999 | 3.5 years | Heart failure | Patients with no diagnosis of heart failure and an echocardiographic measurement of ejection fraction at baseline | During 3.5 years of follow up, 220 patients (2.6%) developed congestive heart failure. | Proportion of cases with heart failure out of total summarised for post MI incidence. Further Cox PH modelling focussed on impact of risk factors on outcomes |
| Ezekowitz, 2009[39]  Linked data across 5 sources; Alberta Canada | 7,733 | MI | 1994-2000 | 5 years | Heart failure | First MI, patients aged >=65 years of age. Patients with admission for MI or HF in the year prior to study entry excluded. No overall cohort descriptives listed - but of those who developed HF post MI, 20.4% had prior AF, 23.2% prior diabetes, 6.5% peripheral arterial disease, 7.4% cerebrovascular disease, 5.9% chronic renal disease, 16.9% COPD and 3.4% Cancer. | During the index MI hospitalisation, 2,831 (37%) MI patients were diagnosed with new HF and 1,024 (13%) died. Among hospital survivors who did not have HF during their index hospitalization (n= 4,291), an additional 3,040 patients (71%) developed HF by 5 years, 64% of which occurred in the first year. In total, 5,871 (76%) elderly patients who survived their first MI developed HF over 5 years. | Relative risks, odds ratios and hazard ratios. Multistate modelling of MI to HF and death without HF as a competing risk. |
| Gerber, 2016[40]  Inpatient and outpatient healthcare records, Olmsted County, Minnesota. | 2,596 | MI | 1990-2010 | 7.6 years | Heart failure | Index MI cases without prior HF. 61% hypertension, 21% diabetes, 11% AF and Charlson co-morbidity index median 1 (IQR 0-2). | 902 patients developed HF (425 [47%] within 3 days; 563 [62%] HFrEF), 535 experienced a recurrent MI (which occurred on the same day or preceded HF in 127 patients [14% of HF cases]), and 1,116 died. | Raw case numbers/percentages for post MI incidence of heart failure. Further analyses consisted of death rates with person-time denominators for HF and HF-free categories - but not for the incidence of heart failure itself. Cox PH modelling (no competing risk analyses). |
| Jhaveri 2012[41]  Clinical Trial | 2,201 | MI | Not stated | 7 years | Heart failure | Non index MI patients, mean age 61.1 years; 67% male. 2.4% of patients included in the study had a prior history of HF. | There were 150 (6.8%) adjudicated HF hospitalisations during a mean follow-up of 6 years | Non parametric unadjusted KM cumulative incidence |
| Hasin, 2016[42] Prospective cohort study; Olmsted County, Minnesota. | 1,081 | MI | 2002-2010 | 4.9 years | Heart failure and cancer | Index MI | A total of 228 patients developed HF and 98 patients developed cancer. Incidence density rates for cancer diagnosis (per 1,000 person-years) were 33.7 for patients with HF and 15.6 for patients without HF (p = 0.002) | Crude unadjusted incidence rates per 1000 person-years at risk. Subsequent Cox PH models to modelled impact of risk factors on incidence of HF and cancer. |
| Malmborg, 2018[43]  All Danish residents without cancer and MI prior to study start | 122,275 | MI and reference population of 2,871,168 cancer-free and MI free patients | 1996-2012 | 17 years | Cancer | All Danish residents aged 30–99 years in 1996 without prior cancer or MI were included. MI population 61.2% male, baseline COPD 9%, Dyslipidaemia 17.6%, Diabetes 12.4%, hypertension 14.1%. Average age MI cohort 59.2 years versus 49.5 years in reference cohort. | Crude incidence rate of cancer 19.1/1000 person-years (MI) vs. Crude incidence rate of cancer 9.3/1000 person-years (reference).   Increased risk of overall cancer IRR 1.08, [95% CI 1.03,1.13] for MI versus no MI; No significant difference in rate of cancer occurring at least 6 months post MI (IRR 1.00, 95% CI 0.96,1.05. Overall IRRs for lung cancer IRR 1.16 [95% CI 1.03,1.30]; colorectal cancer IRR 0.97 [95% CI 0.87,1.07]; lower urinary tract cancer IRR 1.22 [95% CI 1.06,1.37]; prostate cancer IRR 0.90 [95% CI 0.76-1.02]; IRR 0.86 [95% CI 0.73,1.00]. | Crude incidence, cumulative incidence and time-dependent multivariable Poisson regression models to examine the incidence rate ratios (IRR). Age stratified analyses with large > 15 year age bands. |
| Lotan, 2017[44]  Multicentre, 8 hospitals in central Israel | 1,486 | MI without prior cancer | 1992-1993 | 21.4 years | Cancer | Admission to a medical centre <65 years of age without cancer prior to MI. Mean age 56 years, 18.7% female | The overall cancer rate per 1000 person-years was 11.8 (95% confidence interval [CI], 10.4,13.3). | Incidence density rate of cancer with person-time denominator was calculated for the entire sample. Fine and Gray sub-distribution hazard regressions model accounting for competing risk of death |
| Albeiruti, 2019[45]  National inpatient sample, claims-based database, 1,000 hospitals across 46 US states | 1,450,696 | STEMI | 2003-2016 | Not defined | Gastrointestinal bleeding | STEMI patients > 18 years. The national inpatient sample did not contain information with regards the timing of gastrointestinal bleeding relative to STEMI, severity of gastrointestinal bleeding or criteria used. | 32,624 (2.2%) were complicated with gastrointestinal bleeding. The incidence of gastrointestinal bleeding after STEMI decreased over time from 2.7% in 2003 to 2.0% in 2016 (P<0.001) | No specific details provided of denominator or methods used in incidence rate calculation |
| Ding, 2021[46]  Prospective observational study in the US and Spain | 3,501 | MI | 2008-2012 | 1 month | Diabetes | MI patients aged 18-55 years of age | Incidence of newly diagnosed diabetes occurring within 1 month of MI was 14.5%. | Raw case numbers/percentage for post MI outcome of newly diagnosed diabetes |
| Rodrigues, 2013[47]   Single center observational prospective cohort study | 1,050 | MI | 2004-2009 | 7 days | Acute kidney injury | Mean age 65 years, 64% male. Co-morbid at baseline with 70% hypertension, 25% diabetes and 14.6% previous MI. | Acute kidney injury occurred in 14.8% and 36.6% of patients | Raw case numbers/percentages for post MI outcomes |
| Bruetto, 2012[48]  Prospective study | 828 | MI | Not stated | 7 days | Acute kidney injury | Non-index MI patients - median age 65 years, 65.5% male. 69% of the patients had a history of hypertension, 36.7% smoked, 25.7% were diabetic, 22.6% were dyslipidaemic, 41.7% had previously used ACEIs/ARBs, 8.8% had prior PCI, 15.5% prior CAD. | 14.6% of the patients in this study developed acute kidney injury. | Proportion of cases with outcome out of total cases and Cox PH modelling |
| Kulik, 2010[49]  Sample of Medicare beneficiaries, US | 29,088 | CAD | 1995-2004 | 10.9 years | Atrial fibrillation | Non-index CAD patients >=65 years of age, excluding patients who died or were re-admitted 30 days after discharge and those with prior AF. No combined estimates of baseline characteristics presented for whole cohort, however, of non-statin users (N=20638), 23.5% had prior MI, 53.4% prior heart failure, 8.1% prior stroke, 5.1% peripheral vascular disease, 82% hypertension, 43.4% diabetes 27% chronic kidney disease. | New-onset AF in patients who received statins occurred in 10.6%, 32.6%, and 51.2%, at 1 year, 5 years, and 10 years, respectively. Corresponding rates in patients who did not fill a statin prescription were 12.9%, 38.3%, and 58.0%. | Non-parametric KM estimates of cumulative incidence function for post CAD rate of atrial fibrillation. Cox PH modelling without competing risk analyses |
| Singh, 2012[50]  Population based study of Medicare beneficiaries, US | 28,620 | CAD | 1995-2004 | 3.8yrs +/- 3 years | Atrial fibrillation | All patients > 65 years of age. Patients with a history of AF before and during hospitalization were excluded. The mean age of the cohort was 78.1+7.0 years; 72.9% of patients were female | New-onset AF within 5 and 10 years was 39.1 and 61.1%, respectively, in patients who received ACEi/ARB, compared 34.9 and 53.6% in patients who did not receive them | Unadjusted cumulative incidence function based on non-parametric KM estimates. Followed by Cox PH models to determine the impact of a range of covariates on post CAD incidence of AF |
| Jabre, 2011[51] Community-based cohort. Olmsted County, MN. | 3,220 | MI | 1983-2007 | 6.6 years | Atrial fibrillation | Index MI hospitalisations. The mean age at the time of MI was 68 years [SD 15] years and 58% of the patients were men. | 729 patients (22.6%) developed AF after MI (218 [30%] within 2 days, 119 [16%] between 3 and 30 days, and 392 [54%] >30 days post MI). The cumulative incidence of AF after MI at 5 years was 19%. | Crude cumulative incidence based on non-parametric KM estimates |
| Sundbøll, 2018[52]  Nationwide population based cohort study, Denmark | 321,842 | MI | 1980-2012 | 35 years | Dementia | Index MI. 63% male, median age 70.3 years. | Cumulative Incidence Function (CIF) of all-cause dementia up to 35 years after MI was 8.7% (2.8% for Alzheimer disease, 1.6% for vascular dementia, and 4.5% for other dementias). No association with all-cause dementia (adjusted HR, 1.01; 95% CI 0.98,1.03) or other dementias (adjusted HR, 0.98; 95% CI, 0.95,1.01) compared with the general population cohort. For Alzheimer disease, the risk was marginally lower (adjusted HR, 0.92; 95% CI 0.88,0.95), whereas risk of vascular dementia was significantly higher (adjusted HR, 1.35; 95% CI 1.28,1.43). | Risk-set matched control cohort 5:1. Unadjusted cumulative incidence functions up to 35 years post MI – but accounting for competing risk of death. |
| Kala, 2016[53]  Consecutive patient cohort at University Hospital Brno, Czech Republic | 79 | STEMI who received primary percutaneous coronary intervention (PPCI) | Not stated | 1 year | Depression/anxiety | Index STEMI patients treated with PPCI, median age 61 years, 78.5% male. Excluded patients with severe chronic disease with poor prognosis (malignancy, more severe cerebral stroke, organ complications of diabetes). Excluded patients >80 years of age. Did not exclude/account for those with existing signs of depression. | Within 24 hours after PPCI, depression symptoms were identified in 17 patients (21.5%); within 12 months of PPCI - 13.8% had depression. | Raw case numbers/percentages for post MI outcome |
| Lane, 2000[54]  Prospective study from coronary care units in two hospitals in West Midlands, England | 288 | MI | 1997-1998 | 1 year | Depression/anxiety | Non index MI patients. Excluded patients who had co-morbidity which was likely to cause death within 12 months (not specified which conditions excluded). 3.1% took anti-depressants prior to MI and were *not* excluded from analyses. Mean age 62.7 years; 25% female. Major depression not included - only those showing symptoms of mild to severe depression. | At baseline, 89 (30.9%) of the patients had mild to severe symptoms of depression. No clear data on prevalence of depression by end of follow up. | Raw case numbers/percentages for post MI outcome |
| Liang, 2014[55]  Cross-sectional single centre/service study | 158 | MI due to spontaneous coronary artery dissection | Not stated | 8.2 years | Depression/anxiety | 158 SCAD MI survivors (97% women; mean age, 45.5 ± 9.3 years) | 51 (33%) patients had received treatment with medications or counselling for depression and 57 (37%) for anxiety following initial SCAD MI. | Raw case numbers/percentages for post MI outcome |
| de Jonge, 2007[56]  Subsample of MIND-IT trial. 10 hospitals, Netherlands, survey follow-up | 1205 | MI | 1999-2002 | 1-year | Depression/anxiety and Type D personality | Index MI patients, excluding those with prior treatment for depression. Mean age 61.2 years, 22.6% female. 12.7% comorbid with diabetes, 12.6% heart failure and 33.9% hypertension. | Of the 1205 patients, 206 patients had a post MI depressive disorder (17.1%) and 224 were identified as having type-D personality (18.6%). | Raw case numbers/percentages for post MI outcome. Logistic regression modelling. |
| Larsen, 2013[57]  Prospective population-based cohort study | 897 | MI | 2009-2009 | 3 months | Depression/anxiety | Index MI patients, mean age 67.0 years, 69% male. 92 (10.1%) of patients had depression at baseline, 6% co-morbid with stroke, 15% diabetes and 3% heart failure. | 167 (18.6%) had depressive symptoms at 3-months follow up. No data on pre-existing symptoms of depression prior MI. | Proportion of cases with each outcome - followed by Cox PH modelling for risk of outcomes associated with symptoms of depression. |
| Roberge, 2010[58]  Three hospitals, questionnaires to assess PTSD | 447 | MI | 2002-2005 | 1 month | Post-traumatic stress disorder (PTSD) | Mean age 59.2 years [SD 11.27]; 75.1% male. 20.6% history of MI, 367% history of cardiovascular disease. | Four per cent of the patients had PTSD and 12% had partial PTSD | Raw case numbers/proportion for post MI incidence of PTSD. Multiple stepwise regression analyses was further used to develop a prediction model of the intensity of PTSD symptoms. |
| Kucharska-Newton, 2017[59]  Multicentre study, 3 hospitals, US. | 676 | MI | 1993-2007 | 3 years | Functional status | 676 Index MI patients, mean age 61.8 years (SD 6.9), 61% male, medical history for MI patients - 8% HF, 44% hypertension, 19% diabetes. | A decline in functional status was observed on average 2 years prior to a myocardial infarction hospitalization and on average 3 years prior to a stroke or heart failure hospitalization. Functional status post MI declined relative to pre-event levels but improved to close to pre–myocardial infarction levels within 3 years. Decline in functional status following incident heart failure and stroke remained over time | Linear regression to estimate patterns of functional status before and after MI. No clear post MI incidence data presented. |
| Nielsen, 2013[60]  Population based cohort study | 880 | MI | 2009 | 3 years | Impact of mental health status on composite cardiovascular outcomes (MI, heart failure, stroke or transient ischaemic attack) | 880 MI patients, mean age and % male by mental health score quartile was 68.9; 68.4; 65.6; 64.5 years and 54.6%; 62.7%; 80.5% and 78.6% respectively. | During the 3 years after MI, the cumulative incidence of the composite endpoint was 47.5% (95% CI 40.9%,54.5%) for persons in the first quartile, 37.0% (95% CI 30.9%,43.9%) in the second quartile, 29.1% (95% CI 23.5%,35.6%) in the third quartile and 15.0% (95% CI 10.8% ,20.5%) in the fourth quartile of mental health status (1st quartile=poor mental health, 4th quartile = good mental health) | No data on post MI incidence of mental health. The cumulative incidence of the composite cardiovascular outcome 3 years after MI was estimated using the cumulative hazards function stratified by quartile of mental health scores. |
| Gerber, 2011[61]  Consecutive Olmsted county Minnosota with matched community controls | 3,321 | MI and 1:1 matched controls | 1979-2006 | 3 years | Osteoporotic Fractures | Mean age at baseline: 67.4 years [SD 14.2]; 43% women. Incident MI cases. | Overall fracture incidence in MI versus controls was 24.1 versus 20.9 per 1000 person years. Fracture rates were stable in the control group (22.2 in 1979-1989; 19.1 in 1990-1999; and 21.7 in 2000-2006, a steady increase was noted among MI patients (16.3; 22.5; and 34.0, respectively). | Fracture incidence rates with person-time denominators were calculated for MI patients and controls |
| Lee, 2011[62]  Prospective longitudinal cohort study | 105 | STEMI | 2007-2008 | 1.5 years | Severe obstructive sleep apnoea | Mean age was 53 ± 10 years, 98% male. | 44 (42%) had severe obstructive sleep apnoea and 61 (58%) non-severe obstructive sleep apnoea | Raw case numbers/proportions of post MI incidence of obstructive sleep apnoea. Event-free survival curves for severe and non-severe obstructive sleep apnoea groups were constructed using the Kaplan-Meier method. |
| Abbreviations: ACEi/ARB – Angiotensin-converting enzyme inhibitors and angiotensin II receptor blockers; ACS – Acute Coronary Syndromes; AF – atrial fibrillation; CAD – coronary artery disease; CIF – cumulative incidence function; COPD – Coronary Obstructive Pulmonary Disease; CPRD – Clinical Practice Research Database; EHR – electronic healthcare record; HES – Hospital Episode Statistics; HF – heart failure; HFrEF – Heart failure with reduced ejection fraction; HR – hazard ratio; IRR – Incidence Rate Ratio; IQR – Interquartile Range; KM – Kaplan-Meier; MI – Myocardial Infarction; MINAP – Myocardial Ischaemia National Audit Project; NSETMI – Non ST-elevation myocardial infarction; PCI – percutaneous coronary intervention; PH – proportional hazards; PPCI – primary percutaneous coronary intervention; PTSD – post-traumatic stress disorder; SCAD - spontaneous coronary artery dissection; SD – standard deviation; STEMI – ST-Elevation Myocardial Infarction; USA – United States of America; vs. – versus. | | | | | | | | |

**REFERENCES**

1. Schmitt VH, Hobohm L, Münzel T, Wenzel P, Gori T, Keller K. Impact of diabetes mellitus on mortality rates and outcomes in myocardial infarction. Diabetes Met. 2021;47(4):101211.

2. Krumholz HM, Normand S-LT, Wang Y. Twenty-year trends in outcomes for older adults with acute myocardial infarction in the United States. JAMA Netw Open. 2019;2(3):e191938-e.

3. Rapsomaniki E, Thuresson M, Yang E, Blin P, Hunt P, Chung S-C, et al. Using big data from health records from four countries to evaluate chronic disease outcomes: a study in 114 364 survivors of myocardial infarction. Eur Heart J Qual Care Clin Outcomes. 2016;Advanced Online:qcw004.

4. Varenhorst C, Hasvold P, Johansson S, Janzon M, Albertsson P, Leosdottir M, et al. Culprit and nonculprit recurrent ischemic events in patients with myocardial infarction: Data from SWEDEHEART (Swedish Web System for Enhancement and Development of Evidence-Based Care in Heart Disease Evaluated According to Recommended Therapies). J Am Heart Assoc. 2018;7 (1)(e007174). PubMed PMID: 620227930.

5. Jernberg T, Hasvold P, Henriksson M, Hjelm H, Thuresson M, Janzon M. Cardiovascular risk in post-myocardial infarction patients: nationwide real world data demonstrate the importance of a long-term perspective. Eur Heart J. 2015;36(19):1163-70.

6. Yang E, Stokes M, Johansson S, Mellstrom C, Magnuson E, Cohen DJ, et al. Clinical and economic outcomes among elderly myocardial infarction survivors in the United States. Cardiovasc Ther. 2016;34(6):450-9. PubMed PMID: 27564212.

7. Gouda P, Savu A, Bainey KR, Kaul P, Welsh RC. Long-term risk of death and recurrent cardiovascular events following acute coronary syndromes. PLoS One. 2021;16(7):e0254008.

8. Brinkert M, Southern DA, James MT, Knudtson ML, Anderson TJ, Charbonneau F. Incidence and Prognostic Implications of Late Bleeding After Myocardial Infarction or Unstable Angina According to Treatment Strategy. Can J Cardiol. 2017;33(8):998-1005. PubMed PMID: 28669702.

9. Li S, Peng Y, Wang X, Qian Y, Xiang P, Wade SW, et al. Cardiovascular events and death after myocardial infarction or ischemic stroke in an older Medicare population. Clin Cardiol. 2019;42(3):391-9. PubMed PMID: 30697776.

10. Roe MT, Li S, Thomas L, Wang TY, Alexander KP, Ohman EM, et al. Long-term outcomes after invasive management for older patients with non-ST-segment elevation myocardial infarction. Circ Cardiovasc Qual Outcomes. 2013;6(3):323-32. PubMed PMID: 23652734.

11. Nedkoff L, Atkins E, Knuiman M, Sanfilippo FM, Rankin J, Hung J. Age-specific gender differences in long-term recurrence and mortality following incident myocardial infarction: a population-based study. Heart Lung Circ. 2015;24(5):442-9. PubMed PMID: 25618449.

12. Guimaraes PO, Krishnamoorthy A, Kaltenbach LA, Anstrom KJ, Effron MB, Mark DB, et al. Accuracy of Medical Claims for Identifying Cardiovascular and Bleeding Events After Myocardial Infarction : A Secondary Analysis of the TRANSLATE-ACS Study. JAMA Cardiol. 2017;2(7):750-7. PubMed PMID: 28538984.

13. Brieger D, Pocock SJ, Blankenberg S, Chen JY, Cohen MG, Granger CB, et al. Two-year outcomes among stable high-risk patients following acute MI. Insights from a global registry in 25 countries. Int J Cardiol. 2020. PubMed PMID: 2004905249.

14. Pocock SJ, Brieger D, Gregson J, Chen JY, Cohen MG, Goodman SG, et al. Predicting risk of cardiovascular events 1 to 3 years post-myocardial infarction using a global registry. Clin Cardiol. 2020;43(1):24-32. PubMed PMID: 2003635562.

15. Grodzinsky A, Arnold SV, Gosch K, Spertus JA, Foody JM, Beltrame J, et al. Angina frequency after acute myocardial infarction in patients without obstructive coronary artery disease. Eur Heart J Qual Care Clin Outcomes. 2015;1(2):92-9.

16. Canivell S, Muller O, Gencer B, Heg D, Klingenberg R, Räber L, et al. Prognosis of cardiovascular and non-cardiovascular multimorbidity after acute coronary syndrome. PloS One. 2018;13(4).

17. Patel A, Goodman SG, Yan AT, Alexander KP, Wong CL, Cheema AN, et al. Frailty and Outcomes After Myocardial Infarction: Insights From the CONCORDANCE Registry. J Am Heart Assoc. 2018;7(18):e009859. PubMed PMID: 30371219.

18. Koohi F, Ahmadi N, Hadaegh F, Safiee S, Azizi F, Khalili D. Trajectories of cardiovascular disease risk and their association with the incidence of cardiovascular events over 18 years of follow-up: The Tehran Lipid and Glucose study. J Transl Med. 2021;19(1):1-9.

19. Barr PR, Harrison W, Smyth D, Flynn C, Lee M, Kerr AJ. Myocardial Infarction Without Obstructive Coronary Artery Disease is Not a Benign Condition (ANZACS-QI 10). Heart Lung Circ. 2018;27(2):165-74. PubMed PMID: 28408093.

20. Maddox TM, Reid KJ, Spertus JA, Mittleman M, Krumholz HM, Parashar S, et al. Angina at 1 year after myocardial infarction: prevalence and associated findings. JAMA Intern Med. 2008;168(12):1310-6. PubMed PMID: 18574088.

21. Frasure-Smith N, Lesperance F, Juneau M, Talajic M, Bourassa MG. Gender, depression, and one-year prognosis after myocardial infarction. Psychosom Med. 1999;61(1):26-37. PubMed PMID: 10024065.

22. Rincon LM, Sanmartin M, Alonso GL, Rodriguez JA, Muriel A, Casas E, et al. A genetic risk score predicts recurrent events after myocardial infarction in young adults. Rev Esp Cardiol. 2019;17. PubMed PMID: 629644606.

23. Biere L, Mateus V, Clerfond G, Grall S, Willoteaux S, Prunier F, et al. Predictive Factors of Pericardial Effusion After a First Acute Myocardial Infarction and Successful Reperfusion. Am J Cardiol. 2015;116(4):497-503. PubMed PMID: 26070221.

24. Jokhadar M, Jacobsen SJ, Reeder GS, Weston SA, Roger VL. Sudden death and recurrent ischemic events after myocardial infarction in the community. Am J Epidemiol. 2004;159(11):1040-6. PubMed PMID: 15155288.

25. Jons C, Jacobsen UG, Joergensen RM, Olsen NT, Dixen U, Johannessen A, et al. The incidence and prognostic significance of new-onset atrial fibrillation in patients with acute myocardial infarction and left ventricular systolic dysfunction: a CARISMA substudy. Heart Rhythm. 2011;8(3):342-8. PubMed PMID: 21093611.

26. Levantesi G, Macchia A, Marfisi R, Franzosi MG, Maggioni AP, Nicolosi GL, et al. Metabolic syndrome and risk of cardiovascular events after myocardial infarction. J Am Coll Cardiol. 2005;46(2):277-83. PubMed PMID: 16022955.

27. Tran DT, Welsh RC, Ohinmaa A, Thanh NX, Kaul P. Resource use and burden of hospitalization, outpatient, physician, and drug costs in short-and long-term care after acute myocardial infarction. Can J Cardiol. 2018;34(10):1298-306.

28. Chen HY, Tisminetzky M, Lapane KL, Yarzebski J, Person SD, Kiefe CI, et al. Decade‐long trends in 30‐day rehospitalization rates after acute myocardial infarction. J Am Heart Assoc. 2015;4(11):e002291.

29. Myers V, Drory Y, Goldbourt U, Gerber Y. Multilevel socioeconomic status and incidence of frailty post myocardial infarction. Int J Cardiol. 2014;170(3):338-43. PubMed PMID: 24275158.

30. Chamberlain AM, Bielinski SJ, Weston SA, Klaskala W, Mills RM, Gersh BJ, et al. Atrial fibrillation in myocardial infarction patients: Impact on health care utilization. Am Heart J. 2013;166(4):753-9. PubMed PMID: 24093857.

31. Robertson KA, Kayhko K, Kekki P. Re-hospitalizations after myocardial infarction on Prince Edward Island: analysis of the reasons. Can J Cardiovasc Nurs. 2003;13(1):16-20. PubMed PMID: 12703101.

32. Kochar A, Doll JA, Liang L, Curran J, Peterson ED. Temporal trends in post myocardial infarction heart failure and outcomes among older adults. J Card Fail. 2022;28(4):531-9.

33. Chen J, Hsieh AF-C, Dharmarajan K, Masoudi FA, Krumholz HM. National trends in heart failure hospitalization after acute myocardial infarction for Medicare beneficiaries: 1998–2010. Circulation. 2013;128(24):2577-84.

34. Desta L, Jernberg T, Löfman I, Hofman-Bang C, Hagerman I, Spaak J, et al. Incidence, temporal trends, and prognostic impact of heart failure complicating acute myocardial infarction: the SWEDEHEART registry (Swedish Web-System for Enhancement and Development of Evidence-Based Care in Heart Disease Evaluated According to Recommended Therapies): a study of 199,851 patients admitted with index acute myocardial infarctions, 1996 to 2008. JACC: Heart Failure. 2015;3(3):234-42.

35. Gjesing A, Gislason GH, Kober L, Gustav Smith J, Christensen SB, Gustafsson F, et al. Nationwide trends in development of heart failure and mortality after first-time myocardial infarction 1997-2010: A Danish cohort study. Eur J Intern Med. 2014;25(8):731-8. PubMed PMID: 25225051.

36. Gho JM, Schmidt AF, Pasea L, Koudstaal S, Pujades-Rodriguez M, Denaxas S, et al. An electronic health records cohort study on heart failure following myocardial infarction in England: incidence and predictors. BMJ Open. 2018;8(3):e018331.

37. Hung J, Teng THK, Finn J, Knuiman M, Briffa T, Stewart S, et al. Trends From 1996 to 2007 in Incidence and Mortality Outcomes of Heart Failure After Acute Myocardial Infarction: A Population‐Based Study of 20 812 Patients With First Acute Myocardial Infarction in W estern A ustralia. J Am Heart Assoc. 2013;2(5):e000172.

38. Marchioli R, Levantesi G, Macchia A, Marfisi RM, Nicolosi GL, Tavazzi L, et al. Vitamin E increases the risk of developing heart failure after myocardial infarction: Results from the GISSI-Prevenzione trial. J Cardiovasc Med. 2006;7(5):347-50. PubMed PMID: 16645413.

39. Ezekowitz JA, Kaul P, Bakal JA, Armstrong PW, Welsh RC, McAlister FA. Declining in-hospital mortality and increasing heart failure incidence in elderly patients with first myocardial infarction. J Am Coll Cardiol. 2009;53(1):13-20. PubMed PMID: 19118718.

40. Gerber Y, Weston SA, Enriquez-Sarano M, Berardi C, Chamberlain AM, Manemann SM, et al. Mortality Associated With Heart Failure After Myocardial Infarction: A Contemporary Community Perspective. Circ Heart Fail. 2016;9(1):e002460. PubMed PMID: 26699392.

41. Jhaveri RR, Reynolds HR, Katz SD, Jeger R, Zinka E, Forman SA, et al. Heart failure in post-MI patients with persistent IRA occlusion: prevalence, risk factors, and the long-term effect of PCI in the Occluded Artery Trial (OAT). J Card Fail. 2012;18(11):813-21. PubMed PMID: 23141853.

42. Hasin T, Gerber Y, Weston SA, Jiang R, Killian JM, Manemann SM, et al. Heart Failure After Myocardial Infarction Is Associated With Increased Risk of Cancer. J Am Coll Cardiol. 2016;68(3):265-71. PubMed PMID: 27417004.

43. Malmborg M, Christiansen CB, Schmiegelow MD, Torp-Pedersen C, Gislason G, Schou M. Incidence of new onset cancer in patients with a myocardial infarction–a nationwide cohort study. BMC Cardiovasc Disord. 2018;18(1):1-9.

44. Lotan K, Goldbourt U, Gerber Y. Smoking Status and Incidence of Cancer After Myocardial Infarction: A Follow-Up Study of over 20 Years. Am J Med. 2017;130(9):1084-91. PubMed PMID: 28396231.

45. Albeiruti R, Chaudhary F, Alqahtani F, Kupec J, Balla S, Alkhouli M. Incidence, predictors, and outcomes of gastrointestinal bleeding in patients admitted with ST-elevation myocardial infarction. Am J Cardiol. 2019;124(3):343-8.

46. Ding Q, Spatz ES, Lipska KJ, Lin H, Spertus JA, Dreyer RP, et al. Newly diagnosed diabetes and outcomes after acute myocardial infarction in young adults. Heart. 2021;107(8):657-66.

47. Rodrigues FB, Bruetto RG, Torres US, Otaviano AP, Zanetta DM, Burdmann EA. Incidence and mortality of acute kidney injury after myocardial infarction: a comparison between KDIGO and RIFLE criteria. PloS One. 2013;8(7):e69998.

48. Bruetto RG, Rodrigues FB, Torres US, Otaviano AP, Zanetta DM, Burdmann EA. Renal function at hospital admission and mortality due to acute kidney injury after myocardial infarction. PLoS One. 2012;7(4):e35496. PubMed PMID: 22539974.

49. Kulik A, Singh JP, Levin R, Avorn J, Choudhry NK. Association between statin use and the incidence of atrial fibrillation following hospitalization for coronary artery disease. Am J Cardiol. 2010;105(12):1655-60. PubMed PMID: 20538110.

50. Singh JP, Kulik A, Levin R, Ellinor PT, Ruskin J, Avorn J, et al. Renin-angiotensin-system modulators and the incidence of atrial fibrillation following hospitalization for coronary artery disease. Europace. 2012;14(9):1287-93. PubMed PMID: 22539600.

51. Jabre P, Jouven X, Adnet F, Thabut G, Bielinski SJ, Weston SA, et al. Atrial fibrillation and death after myocardial infarction: a community study. Circulation. 2011;123(19):2094-100. PubMed PMID: 21536994.

52. Sundbøll J, Horváth-Puhó E, Adelborg K, Schmidt M, Pedersen L, Bøtker HE, et al. Higher risk of vascular dementia in myocardial infarction survivors. Circulation. 2018;137(6):567-77.

53. Kala P, Hudakova N, Jurajda M, Kasparek T, Ustohal L, Parenica J, et al. Depression and anxiety after acute myocardial infarction treated by primary PCI. PLoS One. 2016;11(4):e0152367.

54. Lane D, Carroll D, Ring C, Beevers DG, Lip GYH. The prevalence and persistence of depression and anxiety following myocardial infarction. Br J Health Psychol. 2002;7(1):11-21. PubMed PMID: 34174896.

55. Liang JJ, Tweet MS, Hayes SE, Gulati R, Hayes SN. Prevalence and predictors of depression and anxiety among survivors of myocardial infarction due to spontaneous coronary artery dissection. J Cardiopulm Rehabil Prev. 2014;34(2):138-42. PubMed PMID: 24280906.

56. de Jonge P, Denollet J, van Melle JP, Kuyper A, Honig A, Schene AH, et al. Associations of type-D personality and depression with somatic health in myocardial infarction patients. J Psychosom Res. 2007;63(5):477-82. PubMed PMID: 17980219.

57. Larsen KK, Christensen B, Søndergaard J, Vestergaard M. Depressive symptoms and risk of new cardiovascular events or death in patients with myocardial infarction: a population-based longitudinal study examining health behaviors and health care interventions. PloS One. 2013;8(9):e74393.

58. Roberge MA, Dupuis G, Marchand A. Post-traumatic stress disorder following myocardial infarction: prevalence and risk factors. Can J Cardiol. 2010;26(5):e170-5. PubMed PMID: 20485697.

59. Kucharska-Newton A, Griswold M, Yao ZH, Foraker R, Rose K, Rosamond W, et al. Cardiovascular Disease and Patterns of Change in Functional Status Over 15 Years: Findings From the Atherosclerosis Risk in Communities (ARIC) Study. J Am Heart Assoc. 2017;6(3):01. PubMed PMID: 28249844.

60. Nielsen TJ, Vestergaard M, Christensen B, Christensen KS, Larsen KK. Mental health status and risk of new cardiovascular events or death in patients with myocardial infarction: A population-based cohort study. BMJ Open. 2013;3 (8) (no pagination)(e003045). PubMed PMID: 369880459.

61. Gerber Y, Melton LJ, 3rd, Weston SA, Roger VL. Association between myocardial infarction and fractures: an emerging phenomenon. Circulation. 2011;124(3):297-303. PubMed PMID: 21709062.

62. Lee CH, Khoo SM, Chan MY, Wong HB, Low AF, Phua QH, et al. Severe obstructive sleep apnea and outcomes following myocardial infarction. J Clin Sleep Med. 2011;7(6):616-21. PubMed PMID: 22171200.
