# Supplementary material for: Health outcomes after myocardial infarction: A population study of 56 million people in England
Source: PLoS Med. 2024 Feb 15;21(2):e1004343. doi: 10.1371/journal.pmed.1004343 (PMC10868847; doi:10.1371/journal.pmed.1004343)
Supplement: S2 Table — ICD10 and OPCS Coding lists adapted from published: https://www.caliberresearch.org/portal. NEC, not elsewhere classifiable; NOC, not otherwise specified. (DOCX) [file pmed.1004343.s007.docx]

| **Label** | **Code** | **Coding System** | **Description** | | | |
| --- | --- | --- | --- | --- | --- | --- |
| **Myocardial Infarction** |  |  |  | | | |
|  | I21 | ICD10 | Acute myocardial infarction | | | |
|  | I22 | ICD10 | Subsequent myocardial infarction | | | |
|  | I23 | ICD10 | Certain current complications following acute myocardial infarction | | | |
| **Heart failure** | | | | |  | |
|  | I50 | ICD10 | Heart failure | | | |
|  | I110 | ICD10 | Hypertensive heart disease with (congestive) heart failure | | | |
|  | I130 | ICD10 | Hypertensive heart and renal disease with (congestive) heart failure | | | |
|  | I132 | ICD10 | Hypertensive heart and renal disease with both (congestive) heart failure and renal failure | | | |
| **Cerebrovascular disease** *(with subgroup codes for Stroke indicated in italics)* | | | | | | |
|  | I65 | ICD10 | Occlusion and stenosis of pre-cerebral arteries, not resulting in cerebral infarction | | | |
|  | I66 | ICD10 | Occlusion and stenosis of cerebral arteries, not resulting in cerebral infarction | | | |
|  | I67 | ICD10 | Other cerebrovascular diseases | | | |
|  | I68 | ICD10 | Cerebrovascular disorders in diseases classified elsewhere | | | |
|  | I69 | ICD10 | Sequelae of cerebrovascular disease | | | |
|  | G45 | ICD10 | Transient cerebral ischaemic attacks and related syndromes | | | |
|  | G46 | ICD10 | Vascular syndromes of brain in cerebrovascular diseases | | | |
| *Cerebrovascular disease: Stroke* | I60 | ICD10 | Subarachnoid haemorrhage | | | |
| *Cerebrovascular disease: Stroke* | I61 | ICD10 | Intracerebral haemorrhage | | | |
| *Cerebrovascular disease: Stroke* | I62 | ICD10 | Other non-traumatic intracranial haemorrhage | | | |
| *Cerebrovascular disease: Stroke* | I63 | ICD10 | Cerebral infarction | | | |
| *Cerebrovascular disease: Stroke* | I64 | ICD10 | Stroke, not specified as haemorrhage or infarction | | | |
| **Peripheral arterial disease** *(with subgroup codes for aortic disease indicated in italics)* | | | |  | | |
|  | I70 | ICD10 | Atherosclerosis | | | |
|  | I72 | ICD10 | Other aneurysm | | | |
|  | I743 | ICD10 | Embolism and thrombosis of arteries of lower extremities | | | |
|  | I744 | ICD10 | Embolism and thrombosis of arteries of extremities, unspecified | | | |
|  | I745 | ICD10 | Embolism and thrombosis of iliac artery | | | |
|  | L50 | OPCS | Other emergency bypass of iliac artery | | | |
|  | L51 | OPCS | Other bypass of iliac artery | | | |
|  | L52 | OPCS | Reconstruction of iliac artery | | | |
|  | L53 | OPCS | Other open operations on iliac artery | | | |
|  | L58 | OPCS | Other emergency bypass of femoral artery | | | |
|  | L59 | OPCS | Other bypass of femoral artery | | | |
|  | L60 | OPCS | Reconstruction of femoral artery | | | |
|  | L62 | OPCS | Other open operations on femoral artery | | | |
|  | L65 | OPCS | Revision of reconstruction of artery | | | |
|  | L541 | OPCS | Percutaneous transluminal angioplasty of iliac artery | | | |
|  | L542 | OPCS | Percutaneous transluminal embolectomy of iliac artery | | | |
|  | L544 | OPCS | Percutaneous transluminal insertion of stent into iliac artery | | | |
|  | L548 | OPCS | Other specified transluminal operations on iliac artery | | | |
|  | L549 | OPCS | Unspecified transluminal operations on iliac artery | | | |
| *Peripheral arterial disease: Aortic disease* | I71 | ICD10 | Aortic aneurysm and dissection | | | |
| **Atrial fibrillation** | | | | | |  |
|  | I48 | ICD10 | Atrial fibrillation and flutter | | | |
| **Severe bleeding** (with subgroup codes for gastrointestinal bleeding indicated in italics) | | | | | |  |
|  | H356 | ICD10 | Retinal haemorrhage | | | |
|  | H431 | ICD10 | Vitreous haemorrhage | | | |
|  | H450 | ICD10 | Vitreous haemorrhage in diseases classified elsewhere | | | |
|  | I230 | ICD10 | Haemopericardium as current complication following acute myocardial infarction | | | |
|  | I312 | ICD10 | Haemopericardium, not elsewhere classified | | | |
|  | I60 | ICD10 | Subarachnoid haemorrhage | | | |
|  | I61 | ICD10 | Intracerebral haemorrhage | | | |
|  | I62 | ICD10 | Other nontraumatic intracranial haemorrhage | | | |
|  | I690 | ICD10 | Sequelae of subarachnoid haemorrhage | | | |
|  | I692 | ICD10 | Sequelae of other nontraumatic intracranial haemorrhage | | | |
| *Severe bleeding: Gastrointestinal* | I850 | ICD10 | Oesophageal varices with bleeding | | | |
| *Severe bleeding: Gastrointestinal* | K226 | ICD10 | Gastro-oesophageal laceration-haemorrhage syndrome | | | |
| *Severe bleeding: Gastrointestinal* | K250 | ICD10 | Gastric ulcer ; Acute with haemorrhage | | | |
| *Severe bleeding: Gastrointestinal* | K252 | ICD10 | Gastric ulcer ; Acute with both haemorrhage and perforation | | | |
| *Severe bleeding: Gastrointestinal* | K254 | ICD10 | Gastric ulcer ; Chronic or unspecified with haemorrhage | | | |
| *Severe bleeding: Gastrointestinal* | K256 | ICD10 | Gastric ulcer ; Chronic or unspecified with both haemorrhage and perforation | | | |
| *Severe bleeding: Gastrointestinal* | K260 | ICD10 | Duodenal ulcer ; Acute with haemorrhage | | | |
| *Severe bleeding: Gastrointestinal* | K262 | ICD10 | Duodenal ulcer ; Acute with both haemorrhage and perforation | | | |
| *Severe bleeding: Gastrointestinal* | K264 | ICD10 | Duodenal ulcer ; Chronic or unspecified with haemorrhage | | | |
| *Severe bleeding: Gastrointestinal* | K266 | ICD10 | Duodenal ulcer ; Chronic or unspecified with both haemorrhage and perforation | | | |
| *Severe bleeding: Gastrointestinal* | K270 | ICD10 | Peptic ulcer, site unspecified ; Acute with haemorrhage | | | |
| *Severe bleeding: Gastrointestinal* | K272 | ICD10 | Peptic ulcer, site unspecified ; Acute with both haemorrhage and perforation | | | |
| *Severe bleeding: Gastrointestinal* | K274 | ICD10 | Peptic ulcer, site unspecified ; Chronic or unspecified with haemorrhage | | | |
| *Severe bleeding: Gastrointestinal* | K276 | ICD10 | Peptic ulcer, site unspecified ; Chronic or unspecified with both haemorrhage and perforation | | | |
| *Severe bleeding: Gastrointestinal* | K280 | ICD10 | Gastrojejunal ulcer ; Acute with haemorrhage | | | |
| *Severe bleeding: Gastrointestinal* | K282 | ICD10 | Gastrojejunal ulcer ; Acute with both haemorrhage and perforation | | | |
| *Severe bleeding: Gastrointestinal* | K284 | ICD10 | Gastrojejunal ulcer ; Chronic or unspecified with haemorrhage | | | |
| *Severe bleeding: Gastrointestinal* | K286 | ICD10 | Gastrojejunal ulcer ; Chronic or unspecified with both haemorrhage and perforation | | | |
| *Severe bleeding: Gastrointestinal* | K290 | ICD10 | Acute haemorrhagic gastritis | | | |
| *Severe bleeding: Gastrointestinal* | K625 | ICD10 | Haemorrhage of anus and rectum | | | |
| *Severe bleeding: Gastrointestinal* | K661 | ICD10 | Haemoperitoneum | | | |
| *Severe bleeding: Gastrointestinal* | K920 | ICD10 | Haematemesis | | | |
| *Severe bleeding: Gastrointestinal* | K921 | ICD10 | Melaena | | | |
| *Severe bleeding: Gastrointestinal* | K922 | ICD10 | Gastrointestinal haemorrhage, unspecified | | | |
|  | N02 | ICD10 | Recurrent and persistent haematuria | | | |
|  | N837 | ICD10 | Haematoma of broad ligament | | | |
|  | N938 | ICD10 | Other specified abnormal uterine and vaginal bleeding | | | |
|  | N939 | ICD10 | Abnormal uterine and vaginal bleeding, unspecified | | | |
|  | R041 | ICD10 | Haemorrhage from throat | | | |
|  | R042 | ICD10 | Haemoptysis | | | |
|  | R048 | ICD10 | Haemorrhage from other sites in respiratory passages | | | |
|  | R049 | ICD10 | Haemorrhage from respiratory passages, unspecified | | | |
|  | R31 | ICD10 | Unspecified haematuria | | | |
|  | R58 | ICD10 | Heamorrhage not elsewhere classified | | | |
|  | S064 | ICD10 | Epidural haemorrhage | | | |
|  | S065 | ICD10 | Traumatic subdural haemorrhage | | | |
|  | S066 | ICD10 | Traumatic subarachnoid haemorrhage | | | |
|  | T810 | ICD10 | Haemorrhage and haematoma complicating a procedure, not elsewhere classified | | | |
|  | A052 | OPCS | Evacuation of haematoma from temporal lobe of brain | | | |
|  | A053 | OPCS | Evacuation of haematoma from cerebellum | | | |
|  | A054 | OPCS | Evacuation of intracerebral haematoma NEC | | | |
|  | A103 | OPCS | Aspiration of haematoma of tissue of brain | | | |
|  | A401 | OPCS | Evacuation of extradural haematoma | | | |
|  | A411 | OPCS | Evacuation of subdural haematoma | | | |
|  | D041 | OPCS | Drainage of haematoma of external ear | | | |
|  | E05 | OPCS | Surgical arrest of bleeding from internal nose | | | |
|  | E203 | OPCS | Surgical arrest of postoperative bleeding of adenoid | | | |
|  | F162 | OPCS | Surgical arrest of postoperative bleeding from tooth socket | | | |
|  | F365 | OPCS | Surgical arrest of postoperative bleeding from tonsillar bed | | | |
|  | G523 | OPCS | Oversew of blood vessel of duodenal ulcer | | | |
|  | H212 | OPCS | Fibreoptic endoscopic coagulation of blood vessel of colon | | | |
|  | H531 | OPCS | Evacuation of perianal haematoma | | | |
|  | K681 | OPCS | Decompression of cardiac tamponade | | | |
|  | P093 | OPCS | Evacuation of haematoma from vulva | | | |
|  | P271 | OPCS | Evacuation of haematoma from vagina | | | |
|  | T032 | OPCS | Reopening of chest and re-exploration of intrathoracic operation site and surgical arrest of postoperative bleeding | | | |
|  | T301 | OPCS | Reopening of abdomen and re-exploration of intra-abdominal operation site and surgical arrest of postoperative bleeding | | | |
|  | V032 | OPCS | Reopening of cranium and re-exploration of intracranial operation site and surgical arrest of postoperative bleeding | | | |
|  | Y221 | OPCS | Aspiration of haematoma of organ NOC | | | |
|  | Y321 | OPCS | Re-exploration of organ and surgical arrest of postoperative bleeding NOC | | | |
| **Dementia** *(with specific code for vascular dementia indicated in italics)* | | | | | |  |
|  | F00 | ICD10 | Dementia in Alzheimer's disease | | | |
| *Dementia: vascular dementia* | F01 | ICD10 | Vascular dementia | | | |
|  | F02 | ICD10 | dementia in other disease classified elsewhere | | | |
|  | F03 | ICD10 | Unspecified dementia | | | |
|  | F051 | ICD10 | Delirium superimposed on dementia | | | |
| **Renal failure** (with subgroup codes for acute and chronic renal failure indicated in italics) | | | | | |  |
| *Acute renal failure* | N00 | ICD10 | Acute nephritic syndrome | | | |
| *Acute renal failure* | N01 | ICD10 | Rapidly progressive nephritic syndrome | | | |
| *Acute renal failure* | N10 | ICD10 | Acute tubulo-interstitial nephritis | | | |
| *Acute renal failure* | N17 | ICD10 | Acute renal failure | | | |
| *Chronic renal failure* | N03 | ICD10 | Chronic nephritic syndrome | | | |
| *Chronic renal failure* | N052 | ICD10 | Unspecified nephritic syndrome ; Diffuse membranous glomerulonephritis | | | |
| *Chronic renal failure* | N053 | ICD10 | Unspecified nephritic syndrome ; Diffuse mesangial proliferative glomerulonephritis | | | |
| *Chronic renal failure* | N054 | ICD10 | Unspecified nephritic syndrome ; Diffuse endocapillary proliferative glomerulonephritis | | | |
| *Chronic renal failure* | N055 | ICD10 | Unspecified nephritic syndrome ; Diffuse mesangiocapillary glomerulonephritis | | | |
| *Chronic renal failure* | N056 | ICD10 | Unspecified nephritic syndrome ; Dense deposit disease | | | |
| *Chronic renal failure* | N181 | ICD10 | Chronic kidney disease, stage 1 | | | |
| *Chronic renal failure* | N182 | ICD10 | Chronic kidney disease, stage 2 | | | |
| *Chronic renal failure* | N183 | ICD10 | Chronic kidney disease, stage 3 | | | |
| *Chronic renal failure* | N184 | ICD10 | Chronic kidney disease, stage 4 | | | |
| *Chronic renal failure* | N185 | ICD10 | Chronic kidney disease, stage 5 | | | |
| *Chronic renal failure* | N189 | ICD10 | Chronic kidney disease, unspecified | | | |
| *Chronic renal failure* | N19 | ICD10 | Unspecified kidney failure | | | |
| *Chronic renal failure* | N25 | ICD10 | Disorders resulting from impaired renal tubular function | | | |
| *Chronic renal failure* | T861 | ICD10 | Kidney transplant failure and rejection | | | |
| *Chronic renal failure* | Y841 | ICD10 | Kidney dialysis | | | |
| *Chronic renal failure* | Z49 | ICD10 | Care involving dialysis | | | |
| *Chronic renal failure* | Z940 | ICD10 | Kidney transplant status | | | |
| *Chronic renal failure* | Z992 | ICD10 | Dependence on renal dialysis | | | |
| **Mood affective disorders** | | | | | |  |
|  | F30 | ICD10 | Manic episode | | | |
|  | F31 | ICD10 | Bipolar affective disorder | | | |
|  | F32 | ICD10 | Depressive episode | | | |
|  | F33 | ICD10 | Recurrent depressive disorder | | | |
|  | F34 | ICD10 | Persistent mood disorder | | | |
|  | F38 | ICD10 | Other mood affective disorders | | | |
|  | F39 | ICD10 | Unspecified mood affective disorders | | | |
| **Diabetes Mellitus** | | | | | | |
|  | E10 | ICD10 | Insulin-dependent diabetes mellitus | | | |
|  | E11 | ICD10 | Non-insulin-dependent diabetes mellitus | | | |
|  | E12 | ICD10 | Malnutrition-related diabetes mellitus | | | |
|  | E13 | ICD10 | Other specified diabetes mellitus | | | |
|  | E14 | ICD10 | Unspecified diabetes mellitus | | | |
|  | G590 | ICD10 | Diabetic mononeuropathy | | | |
|  | G632 | ICD10 | Diabetic polyneuropathy | | | |
|  | H280 | ICD10 | Diabetic cataract | | | |
|  | H360 | ICD10 | Diabetic retinopathy | | | |
|  | M142 | ICD10 | Diabetic arthropathy | | | |
|  | N083 | ICD10 | Glomerular disorders in diabetes mellitus | | | |
| **Cancer** (with subgroup codes for colorectal cancer, lung cancer, breast cancer and prostate cancer indicated in italics) | | | | | |  |
|  | C00 | ICD10 | Malignant neoplasm of lip | | | |
|  | C01 | ICD10 | Malignant neoplasm of base of tongue | | | |
|  | C02 | ICD10 | Malignant neoplasm of other and unspecified parts of tongue | | | |
|  | C03 | ICD10 | Malignant neoplasm of gum | | | |
|  | C04 | ICD10 | Malignant neoplasm of floor of mouth | | | |
|  | C05 | ICD10 | Malignant neoplasm of palate | | | |
|  | C06 | ICD10 | Malignant neoplasm of other and unspecified parts of mouth | | | |
|  | C07 | ICD10 | Malignant neoplasm of parotid gland | | | |
|  | C08 | ICD10 | Malignant neoplasm of other and unspecified major salivary glands | | | |
|  | C09 | ICD10 | Malignant neoplasm of tonsil | | | |
|  | C10 | ICD10 | Malignant neoplasm of oropharynx | | | |
|  | C11 | ICD10 | Malignant neoplasm of nasopharynx | | | |
|  | C12 | ICD10 | Malignant neoplasm of pyriform sinus | | | |
|  | C13 | ICD10 | Malignant neoplasm of hypopharynx | | | |
|  | C14 | ICD10 | Malignant neoplasm of other and ill-defined sites in the lip, oral cavity and pharynx | | | |
|  | C30 | ICD10 | Malignant neoplasm of nasal cavity and middle ear | | | |
|  | C31 | ICD10 | Malignant neoplasm of accessory sinuses | | | |
|  | C32 | ICD10 | Malignant neoplasm of larynx | | | |
|  | C15 | ICD10 | Malignant neoplasm of esophagus | | | |
|  | C16 | ICD10 | Malignant neoplasm of stomach | | | |
|  | C17 | ICD10 | Malignant neoplasm of small intestine | | | |
| *Cancer: Colorectal* | C18 | ICD10 | Malignant neoplasm of colon | | | |
| *Cancer: Colorectal* | C19 | ICD10 | Malignant neoplasm of rectosigmoid junction | | | |
| *Cancer: Colorectal* | C20 | ICD10 | Malignant neoplasm of rectum | | | |
|  | C21 | ICD10 | Malignant neoplasm of anus and anal canal | | | |
|  | C22 | ICD10 | Malignant neoplasm of liver and intrahepatic bile ducts | | | |
|  | C23 | ICD10 | Malignant neoplasm of gallbladder | | | |
|  | C24 | ICD10 | Malignant neoplasm of other and unspecified parts of biliary tract | | | |
|  | C25 | ICD10 | Malignant neoplasm of pancreas | | | |
|  | C26 | ICD10 | Malignant neoplasm of other and ill-defined digestive organs | | | |
| *Cancer: Lung* | C33 | ICD10 | Malignant neoplasm of trachea | | | |
| *Cancer: Lung* | C34 | ICD10 | Malignant neoplasm of bronchus and lung | | | |
| *Cancer: Lung* | C37 | ICD10 | Malignant neoplasm of thymus | | | |
|  | C38 | ICD10 | Malignant neoplasm of heart, mediastinum and pleura | | | |
|  | C39 | ICD10 | Malignant neoplasm of other and ill-defined sites in the respiratory system and intrathoracic organs | | | |
|  | C40 | ICD10 | Malignant neoplasm of bone and articular cartilage of limbs | | | |
|  | C41 | ICD10 | Malignant neoplasm of bone and articular cartilage of other and unspecified sites | | | |
|  | C43 | ICD10 | Malignant melanoma of skin | | | |
|  | C44 | ICD10 | Other and unspecified malignant neoplasm of skin | | | |
|  | C45 | ICD10 | Mesothelioma | | | |
|  | C46 | ICD10 | Kaposi's sarcoma | | | |
|  | C47 | ICD10 | Malignant neoplasm of peripheral nerves and autonomic nervous system | | | |
|  | C48 | ICD10 | Malignant neoplasm of retroperitoneum and peritoneum | | | |
|  | C49 | ICD10 | Malignant neoplasm of other connective and soft tissue | | | |
| *Cancer: Breast* | C50 | ICD10 | Malignant neoplasm of breast | | | |
|  | C51 | ICD10 | Malignant neoplasm of vulva | | | |
|  | C52 | ICD10 | Malignant neoplasm of vagina | | | |
|  | C53 | ICD10 | Malignant neoplasm of cervix uteri | | | |
|  | C54 | ICD10 | Malignant neoplasm of corpus uteri | | | |
|  | C55 | ICD10 | Malignant neoplasm of uterus, part unspecified | | | |
|  | C56 | ICD10 | Malignant neoplasm of ovary | | | |
|  | C57 | ICD10 | Malignant neoplasm of other and unspecified female genital organs | | | |
|  | C58 | ICD10 | Malignant neoplasm of placenta | | | |
|  | C60 | ICD10 | Malignant neoplasm of penis | | | |
| *Cancer: Prostate* | C61 | ICD10 | Malignant neoplasm of prostate | | | |
|  | C62 | ICD10 | Malignant neoplasm of testis | | | |
|  | C63 | ICD10 | Malignant neoplasm of other and unspecified male genital organs | | | |
|  | C64 | ICD10 | Malignant neoplasm of kidney, except renal pelvis | | | |
|  | C65 | ICD10 | Malignant neoplasm of renal pelvis | | | |
|  | C66 | ICD10 | Malignant neoplasm of ureter | | | |
|  | C68 | ICD10 | Malignant neoplasm of other and unspecified urinary organs | | | |
|  |  |  |  | | | |
|  | C67 | ICD10 | Malignant neoplasm of bladder | | | |
|  | C69 | ICD10 | Malignant neoplasm of eye and adnexa | | | |
|  | C70 | ICD10 | Malignant neoplasm of meninges | | | |
|  | C71 | ICD10 | Malignant neoplasm of brain | | | |
|  | C72 | ICD10 | Malignant neoplasm of spinal cord, cranial nerves and other parts of central nervous system | | | |
|  | C75 | ICD10 | Malignant neoplasm of other endocrine glands and related structures | | | |
|  | C73 | ICD10 | Malignant neoplasm of thyroid gland | | | |
|  | C74 | ICD10 | Malignant neoplasm of adrenal gland | | | |
|  | C76 | ICD10 | Malignant neoplasm of other and ill-defined sites | | | |
|  | C77 | ICD10 | Secondary and unspecified malignant neoplasm of lymph nodes | | | |
|  | C78 | ICD10 | Secondary malignant neoplasm of respiratory and digestive organs | | | |
|  | C79 | ICD10 | Secondary malignant neoplasm of other and unspecified sites | | | |
|  | C80 | ICD10 | Malignant neoplasm without specification of site | | | |
|  | C81 | ICD10 | Hodgkin lymphoma | | | |
|  | C82 | ICD10 | Follicular lymphoma | | | |
|  | C83 | ICD10 | Non-follicular lymphoma | | | |
|  | C84 | ICD10 | Mature T/NK-cell lymphomas | | | |
|  | C85 | ICD10 | Other specified and unspecified types of non-Hodgkin lymphoma | | | |
|  | C88 | ICD10 | Malignant immunoproliferative diseases and certain other B-cell lymphomas | | | |
|  | C90 | ICD10 | Multiple myeloma and malignant plasma cell neoplasms | | | |
|  | C91 | ICD10 | Lymphoid leukemia | | | |
|  | C92 | ICD10 | Myeloid leukemia | | | |
|  | C93 | ICD10 | Monocytic leukemia | | | |
|  | C94 | ICD10 | Other leukemias of specified cell type | | | |
|  | C95 | ICD10 | Leukemia of unspecified cell type | | | |
|  | C96 | ICD10 | Other and unspecified malignant neoplasms of lymphoid, hematopoietic and related tissue | | | |
|  | C97 | ICD10 | Malignant neoplasms of independent (primary) multiple sites | | | |
| **Cardiovascular risk factors** |  |  |  | | | |
| **Hypertension** |  |  |  | | | |
|  | 119.X | ICD10 | Essential (primary) hypertension | | | |
|  | I11.0 | ICD10 | Hypertensive heart disease with (congestive) heart failure | | | |
|  | 111.9 | ICD10 | Hypertensive heart disease without (congestive) heart failure | | | |
|  | I12.0 | ICD10 | Hypertensive renal disease with renal failure | | | |
|  | I12.9 | ICD10 | Hypertensive renal disease without renal failure | | | |
|  | I13.0 | ICD10 | Hypertensive heart and renal disease with (congestive) heart failure | | | |
|  | I13.1 | ICD10 | Hypertensive heart and renal disease with renal failure | | | |
|  | I13.2 | ICD10 | Hypertensive heart and renal disease with both (congestive) heart failure and renal failure | | | |
|  | I13.9 | ICD10 | Hypertensive heart and renal disease, unspecified | | | |
|  | I15.0 | ICD10 | Renovascular hypertension | | | |
|  | I15.1 | ICD10 | Hypertension secondary to other Penal disorders | | | |
|  | I15.2 | ICD10 | Hypertension secondary to endocrine disorders | | | |
|  | I15.8 | ICD10 | Other secondary hypertension | | | |
|  | I15.9 | ICD10 | Secondary hypertension, unspecified | | | |
|  | I67.4 | ICD10 | Hypertensive encephalopathy | | | |
| **Dyslipidaemia** |  |  |  | | | |
|  | E78.0 | ICD10 | Pure hypercholesterolaemia | | | |
|  | E78.1 | ICD10 | Pure hyperglyceridaemia | | | |
|  | E78.2 | ICD10 | Mixed hyperlipidaemia | | | |
|  | E78.3 | ICD10 | Hyperchylomicronaemia | | | |
|  | E78.4 | ICD10 | Other hyperlipidaemia | | | |
|  | E78.5 | ICD10 | Hyperlipidaemia, unspecified | | | |
|  | E78.6 | ICD10 | Lipoprotein deficiency | | | |
|  | E78.8 | ICD10 | Other disorders of lipoprotein metabolism | | | |
|  | E78.9 | ICD10 | Disorder of lipoprotein metabolism, unspecified | | | |
| **Obesity** |  |  |  | | | |
|  | E66.0 | ICD10 | Obesity due to excess calories | | | |
|  | E66.1 | ICD10 | Drug-induced obesity | | | |
|  | E66.2 | ICD10 | Extreme obesity with alveolar hypoventilation | | | |
|  | E66.8 | ICD10 | Other obesity | | | |
|  | E66.9 | ICD10 | Obesity, unspecified | | | |
| **Tobacco smoking** |  |  |  | | | |
|  | F17.0 | ICD10 | Mental and behavioural disorders due to use of tobacco, acute intoxication | | | |
|  | F17.1 | ICD10 | Mental and behavioural disorders due to use of tobacco, harmful use | | | |
|  | F17.2 | ICD10 | Mental and behavioural disorders due to use of tobacco, dependence syndrome | | | |
|  | F17.3 | ICD10 | Mental and behavioural disorders due to use of tobacco, withdrawal state | | | |
|  | F17.4 | ICD10 | Mental and behavioural disorders due to use of tobacco, withdrawal state with delirium | | | |
|  | F17.5 | ICD10 | Mental and behavioural disorders due to use of tobacco, psychotic disorder | | | |
|  | F17.6 | ICD10 | Mental and behavioural disorders due to use of tobacco, amnesic syndrome | | | |
|  | F17.7 | ICD10 | Mental and behavioural disorders due to use of tobacco, residual and late-onset psychotic disorder | | | |
|  | F17.8 | ICD10 | Mental and behavioural disorders due to use of tobacco, other Mental and behavioural disorders | | | |
|  | F17.9 | ICD10 | Mental and behavioural disorders due to use of tobacco, unspecified Mental and behavioural disorders | | | |
|  | T65.2 | ICD10 | Toxic effect of other and unspecified substances, tobacco and nicotine | | | |
|  | 258.7 | ICD10 | Exposure to tobacco smoke | | | |
|  | F17.0 | ICD10 | Mental and behavioural disorders due to use of tobacco, acute intoxication | | | |
|  | F17.1 | ICD10 | Mental and behavioural disorders due to use of tobacco, harmful use | | | |
| **Alcohol excess** |  |  |  | | | |
|  | F10.0 | ICD10 | Mental and behavioural disorders due to use of alcohol, acute intoxication | | | |
|  | F10.1 | ICD10 | Mental and behavioural disorders due to use of alcohol, harmful use | | | |
|  | F10.2 | ICD10 | Mental and behavioural disorders due to use of alcohol, dependence syndrome | | | |
|  | F19.3 | ICD10 | Mental and behavioural disorders due to use of alcohol, withdrawal state | | | |
|  | F10.4 | ICD10 | Mental and behavioural disorders due to use of alcohol, withdrawal state with delirium | | | |
|  | F19.5 | ICD10 | Mental and behavioural disorders due to use of alcohol, psychotic disorder | | | |
|  | F10.6 | ICD10 | Mental and behavioural disorders due to use of alcohol, amnesic syndrome | | | |
|  | F10.7 | ICD10 | Mental and behavioural disorders due to use of alcohol, residual and late-onset psychotic disorder | | | |
|  | F10.8 | ICD10 | Mental and behavioural disorders due to use of alcohol, other mental and behavioural disorders | | | |
|  | F10.9 | ICD10 | Mental and behavioural disorders due to use of alcohol, unspecified mental and behavioural disorders | | | |
|  | Z72.1 | ICD10 | Alcohol use | | | |
|  | E24.4 | ICD10 | Alcohol-induced pseudo-Cushing's syndrome | | | |
|  | G31.2 | ICD10 | Degeneration of nervous system due to alcohol | | | |
|  | G62.1 | ICD10 | Alcoholic polyneuropathy | | | |
|  | G72.1 | ICD10 | Alcoholic myopathy | | | |
|  | I42.6 | ICD10 | Alcoholic cardiomyopathy | | | |
|  | K29.2 | ICD10 | Alcoholic gastritis | | | |
|  | K70.0 | ICD10 | Alcoholic fatty liver | | | |
|  | K70.1 | ICD10 | Alcoholic hepatitis | | | |
|  | K70.2 | ICD10 | Alcoholic fibrosis and sclerosis of liver | | | |
|  | K70.3 | ICD10 | Alcoholic cirrhosis of liver | | | |
|  | K70.4 | ICD10 | Alcoholic hepatic failure | | | |
|  | K70.9 | ICD10 | Alcoholic liver disease, unspecified | | | |
|  | K85.2 | ICD10 | Alcohol-induced acute pancreatitis | | | |
|  | K86.0 | ICD10 | Alcohol-induced chronic pancreatitis | | | |
|  | Z50.2 | ICD10 | Alcohol rehabilitation | | | |
|  | Z71.4 | ICD10 | Alcohol abuse counselling and surveillance | | | |
| **Invasive coronary strategy** |  |  |  | | | |
| **Invasive coronary angiography** |  |  |  | | | |
|  | K63.1 | OPCS | Angiocardiography of combination of right and left side of heart | | | |
|  | K63.2 | OPCS | Angiocardiography of right side of heart NEC | | | |
|  | K63.3 | OPCS | Angiocardiography of left side of heart NEC | | | |
|  | K63.4 | OPCS | Coronary arteriography using two catheters | | | |
|  | K63.5 | OPCS | Coronary arteriography using single catheter | | | |
|  | K63.6 | OPCS | Coronary arteriography NEC | | | |
|  | K65.1 | OPCS | Catheterisation of combination of right and left side of heart NEC | | | |
|  | K65.2 | OPCS | Catheterisation of right side of heart NEC | | | |
|  | K65.3 | OPCS | Catheterisation of left side of heart NEC | | | |
|  | K65.4 | OPCS | Catheterisation of left side of heart via atrial transeptal puncture | | | |
|  | K65.8 | OPCS | Other specifed catheterisation of heart | | | |
|  | K65.9 | OPCS | Unspecified catheterisation of heart | | | |
| **Percutaneous coronary intervention** |  |  |  | | | |
|  | K49 | OPCS | Transluminal baloon angioplasty of coronary artery | | | |
|  | K49.1 | OPCS | Percutaneous transluminal balloon angioplasty of one coronary artery | | | |
|  | K49.2 | OPCS | Percutaneous transluminal balloon angioplasty of multiple coronary arteries | | | |
|  | K49.3 | OPCS | Percutaneous transluminal balloon angioplasty of bypass graft of coronary artery | | | |
|  | K49.4 | OPCS | Percutaneous transluminal cutting balloon angioplasty of coronary artery | | | |
|  | K49.8 | OPCS | Other specified transluminal balloon angioplasty of coronary artery | | | |
|  | K49.9 | OPCS | Unspecified transluminal balloon angioplasty of coronary artery | | | |
|  | K50 | OPCS | Other therapeutic transluminal operations on coronary artery | | | |
|  | K50.1 | OPCS | Percutaneous transluminal laser coronary angioplasty | | | |
|  | K50.4 | OPCS | Percutaneous transluminal atherectomy of coronary artery | | | |
|  | K50.8 | OPCS | Other specified other therapeutic transluminal operations on coronary artery | | | |
|  | K50.9 | OPCS | Unspecified other therapeutic transluminal operations on coronary artery | | | |
|  | K75 | OPCS | Percutaneous transluminal balloon angioplasty and insertion of stent into coronary artery | | | |
|  | K75.1 | OPCS | Percutaneous transluminal balloon angioplasty and insertion of 1-2 drug-eluting stents into coronary artery | | | |
|  | K7S.2 | OPCS | Percutaneous transluminal balloon angioplasty and insertion of 3 or more drug-eluting stents into coronary artery | | | |
|  | K75.3 | OPCS | Percutaneous transluminal balloon angioplasty and insertion of 1-2 stents into coronary artery | | | |
|  | K7S.4 | OPCS | Percutaneous transluminal balloon angioplasty and insertion of 3 or more stents into coronary artery | | | |
|  | K75.8 | OPCS | Other specified percutaneous transluminal balloon angioplasty and insertion of stent into coronary artery | | | |
|  | K75.9 | OPCS | Unspecified percutaneous transluminal balloon angioplasty and insertion of stent into coronary artery | | | |
| **Coronary artery bypass graft surgery** |  |  |  | | | |
|  | K40.1 | OPCS | Saphenous vein graft replacement of one coronary artery | | | |
|  | K40.2 | OPCS | Saphenous vein graft replacement of two coronary arteries | | | |
|  | K40.3 | OPCS | Saphenous vein graft replacement of three coronary arteries | | | |
|  | K40.4 | OPCS | Saphenous vein graft replacement of four or more coronary arteries | | | |
|  | K40.8 | OPCS | Other specified saphenous vein graft replacement of coronary artery | | | |
|  | K40.9 | OPCS | Unspecified saphenous vein graft replacement of coronary artery | | | |
|  | K41.1 | OPCS | Autograft replacement of one coronary artery | | | |
|  | K41.2 | OPCS | Autograft replacement of two coronary arteries | | | |
|  | K41.3 | OPCS | Autograft replacement of three coronary arteries | | | |
|  | K41.4 | OPCS | Autograft replacement of four or more coronary arteries | | | |
|  | K41.8 | OPCS | Other specified autograft replacement of coronary artery | | | |
|  | K41.9 | OPCS | Unspecified other autograft replacement of coronary artery | | | |
|  | K42.1 | OPCS | Allograft replacement of one coronary artery | | | |
|  | K42.2 | OPCS | Allograft replacement of two coronary arteries | | | |
|  | K42.3 | OPCS | Allograft replacement of three coronary arteries | | | |
|  | K42.4 | OPCS | Allograft replacement of four or more coronary arteries | | | |
|  | K42.8 | OPCS | Other specified allograft replacement of coronary artery | | | |
|  | K42.9 | OPCS | Unspecified other allograft replacement of coronary artery | | | |
|  | K43.1 | OPCS | Prosthetic replacement of one coronary artery | | | |
|  | K43.2 | OPCS | Prosthetic replacement of two coronary arteries | | | |
|  | K43.3 | OPCS | Prosthetic replacement of three coronary arteries | | | |
|  | K43.4 | OPCS | Prosthetic replacement of four or more coronary arteries | | | |
|  | K43.8 | OPCS | Other specified prosthetic replacement of coronary artery | | | |
|  | K43.9 | OPCS | Unspecified other prosthetic replacement of coronary artery | | | |
|  | K44.1 | OPCS | Replacement of coronary arteries using multiple methods | | | |
|  | K44.2 | OPCS | Revision of replacement of coronary artery | | | |
|  | K44.8 | OPCS | Other specified other replacement of coronary artery | | | |
|  | K44.9 | OPCS | Unspecified other replacement of coronary artery | | | |
|  | K45.1 | OPCS | Double anastamosis of mammary arteries to coronary arteries | | | |
|  | K45.2 | OPCS | Double anastamosis of thoracic arteries to coronary arteries NEC | | | |
|  | K45.3 | OPCS | Anastamosis of mammary artery to left anteriod descending coronary artery | | | |
|  | K45.4 | OPCS | Anastamosis of mammary artery to coronary artery NEC | | | |
|  | K45.5 | OPCS | Anastamosis of thoracic artery to coronary artery NEC | | | |
|  | K45.6 | OPCS | Revision of connection of thoracic artery to coronary artery | | | |
|  | K45.8 | OPCS | Other specified connection of thoracic artery to coronary artery | | | |
|  | K45.9 | OPCS | Unspecified connection of thoracic artery to coronary artery | | | |
|  | K46.1 | OPCS | Double implantation of mammary arteries into heart | | | |
|  | K46.2 | OPCS | Double implantation of thoracic arteries into heart NEC | | | |
|  | K46.3 | OPCS | Implantation of mammary artery into heart NEC | | | |
|  | K46.4 | OPCS | Implantation of thoracic artery into heart NEC | | | |
|  | K46.5 | OPCS | Revision of implantation of thoracic artery into heart | | | |
|  | K46.8 | OPCS | Other specified other bypass of coronary artery | | | |
|  | K46.9 | OPCS | Unspecified other bypass of coronary artery | | | |
|  | K47.1 | OPCS | Endarterectomy of coronary artery | | | |
|  | K47.2 | OPCS | Repair of arteriovenous fistula of coronary artery | | | |
|  | K47.3 | OPCS | Repair of aneurysm of coronary artery | | | |
|  | K47.4 | OPCS | Repair of rupture of coronary artery | | | |
|  | K47.5 | OPCS | Repair of arteriovenous malformation of coronary artery | | | |
|  | K47.8 | OPCS | Other specified repair of coronary artery | | | |
|  | K47.9 | OPCS | Unspecified repair of coronary artery | | | |
|  | K48.1 | OPCS | Transection of muscle bridge of coronary artery | | | |
|  | K48.2 | OPCS | Transposition of coronary artery NEC | | | |
|  | K48.3 | OPCS | Open angioplasty of coronary artery | | | |
|  | K48.4 | OPCS | Exploration of coronary artery | | | |
|  | K48.8 | OPCS | Other specified other open operations on coronary artery | | | |
|  | K48.9 | OPCS | Unspecified other open operations on coronary artery | | | |

ICD10 and OPCS Coding lists adapted from published: <https://www.caliberresearch.org/portal>. Abbreviations: ICD – International Classification of Diseases; OPCS – Operating Procedure Code Supplement Classification of Interventions and Procedures; NEC – Not elsewhere classifiable; NOC – not otherwise specified.
