# Supplementary material for: Health outcomes after myocardial infarction: A population study of 56 million people in England
Source: PLoS Med. 2024 Feb 15;21(2):e1004343. doi: 10.1371/journal.pmed.1004343 (PMC10868847; doi:10.1371/journal.pmed.1004343)
Supplement: S3 Table — aIndividuals were matched according to single year of age, sex, month and year of hospital admission, and NHS Trust using a 5:1 risk-set matching approach. bCases within the matched control cohort who went on to develop MI were censored at time of first MI; therefore, estimates of subsequent MI for this cohort were not included. CI, confidence interval; MI, myocardial infarction; NA, not applicable; NHS, National Health Service. (DOCX) [file pmed.1004343.s008.docx]

| Health outcome | Rate per 1000 pyrs [95% CI] | |
| --- | --- | --- |
|  | MI cohort | Matched control cohort^a^ |
| Subsequent MI | 13.3 [13.1,13.5] | NA^b^ |
| Heart failure | 86.4 [85.9,86.9] | 16.5 [16.4,16.6] |
| Atrial fibrillation | 64.3 [63.9,64.7] | 31.7 [31.5,31.8] |
| Cerebrovascular disease | 27.0 [26.7,27.2] | 20.9 [20.8,21.0] |
| Stroke | 13.2 [13.0,13.38] | 10.4 [10.4,10.5] |
| Peripheral arterial disease | 13.7 [13.5,13.8] | 7.0 [7.0,7.1] |
| Aortic disease | 6.67 [6.54,6.80] | 3.6 [3.6,3.7] |
| Severe bleeding | 45.0 [44.7,45.4] | 36.5 [36.4,36.7] |
| Gastrointestinal bleeding | 18.75 [18.5,19.0] | 16.2 [16.1,16.3] |
| Renal failure | 56.5 [56.1,56.9] | 31.3 [31.2,31.5] |
| Acute | 37.7 [37.4,38.0] | 19.8 [19.7,19.9] |
| Chronic | 32.0 [31.8,32.3] | 18.3 [18.2,18.4] |
| Diabetes mellitus | 53.7 [53.3,54.1] | 30.4 [30.3,30.6] |
| Dementia | 14.6 [14.4,14.8] | 14.3 [14.2,14.4] |
| Vascular dementia | 3.9 [3.8,4.0] | 3.5 [3.4,3.5] |
| Depression | 17.1 [16.9,17.3] | 15.8 [15.7,15.9] |
| Cancer | 26.0 [25.7,26.2] | 44.9 [44.7,45.1] |
| Breast | 1.36 [1.30,1.41] | 2.6 [2.5,2.6] |
| Prostate | 4.55 [4.45,4.66] | 6.8 [6.8,6.9] |
| Lung | 3.48 [3.39,3.58] | 4.8 [4.7,4.8] |
| Colorectal | 2.63 [2.55,2.71] | 4.2 [4.1,4.2] |
| All-cause mortality | 74.6 [74.2,75.0] | 62.8 [62.6,63.0] |
| ^a^Individuals were matched according to single year of age, sex, month and year of hospital admission and NHS Trust using a 5:1 risk-set matching approach. ^b^Cases within the matched control cohort who went on to develop MI were censored at time of first MI, therefore estimates of subsequent MI for this cohort were not included. Abbreviations: CI – confidence interval; MI - myocardial infarction; NA – not applicable; NHS – national health service. | | |
