# Supplementary material for: Health outcomes after myocardial infarction: A population study of 56 million people in England
Source: PLoS Med. 2024 Feb 15;21(2):e1004343. doi: 10.1371/journal.pmed.1004343 (PMC10868847; doi:10.1371/journal.pmed.1004343)
Supplement: S6 Table — aCumulative incidences are presented as percentage of cases expected to develop each outcome by each respective time point and adjusted for nonlinear age using restricted cubic spline functions, sex, calendar year, and deprivation score—treating death without outcome as a competing risk. bIndividuals were matched according to single year of age, sex, month and year of hospital admission, and NHS Trust using a 5:1 risk-set matching approach. cCases within the matched control cohort who went on to develop MI were censored at time of first MI; therefore, estimates of subsequent MI for this cohort were not included. CI, confidence interval; MI, myocardial infarction; NA, not applicable; NHS, National Health Service; SD, standard deviation. (DOCX) [file pmed.1004343.s011.docx]

|  | **Cumulative incidence^a^ [95% CI] 1 year post study entry** | | **Cumulative incidence^a^ [95% CI] 5 years post study entry** | | **Cumulative incidence^a^ [95% CI] 9 years post study entry** | |
| --- | --- | --- | --- | --- | --- | --- |
|  | Post MI outcomes | Matched control^b^ outcomes | Post MI outcomes | Matched control^b^ outcomes | Post MI outcomes | Matched control^b^ outcomes |
| Subsequent MI | 1.4 [1.4,1.5] | NA^c^ | 4.5 [4.4,4.5] | NA^c^ | 6.4 [6.3,6.5] | NA^c^ |
| Heart failure | 3.4 [3.3,3.4] | 1.09 [1.08,1.11] | 9.5 [9.4,9.6] | 4.51 [4.48,4.55] | 14.3 [14.1,14.5] | 7.6 [7.6,7.7] |
| Atrial fibrillation | 2.6 [2.6,2.7] | 1.80 [1.78,1.82] | 7.7 [7.6,7.8] | 6.93 [6.89,6.98] | 11.8 [11.6,12.0] | 11.2 [11.1,11.3] |
| Cerebrovascular disease | 1.5 [1.4,1.5] | 1.20 [1.18,1.21] | 5.4 [5.3,5.5] | 5.03 [4.99,5.07] | 8.5 [8.3,8.6] | 8.3 [8.1,8.2] |
| *Stroke* | 0.7 [0.7,0.7] | 0.56 [0.54,0.57] | 2.7 [2.6,2.7] | 2.37 [2.35,2.40] | 4.2 [4.1,4.3] | 3.8 [3.7,3.8] |
| Peripheral vascular disease | 0.6 [0.6,0.7] | 0.46 [0.45,0.47] | 2.3 [2.2,2.3] | 1.70 [1.68,1.73] | 3.6 [3.5,3.6] | 2.8 [2.7,2.8] |
| *Aortic disease* | 0.3 [0.3,0.3] | 0.22 [0.21,0.23] | 1.1 [1.1,1.2] | 0.87 [0.85,0.89] | 2.0 [1.9,2.1] | 1.4 [1.4,1.5] |
| Severe bleeding | 1.5 [1.5,1.5] | 2.21 [2.19,2.23] | 9.4 [9.3,9.5] | 8.41 [8.36,8.46] | 13.4 [13.3,13.6] | 12.6 [12.5,12.7] |
| *Gastrointestinal bleeding* | 3.3 [3.3,3.4] | 0.98 [0.96,0.99] | 4.4 [4.3,4.5] | 4.04 [4.00,4.08] | 6.7 [6.5,6.8] | 6.2 [6.2,6.3] |
| Renal failure | 2.7 [2.7,2.8] | 1.86 [1.84,1.89] | 11.3 [11.2,11.4] | 9.02 [8.96,9.07] | 19.4 [19.2,19.7] | 16.3 [16.2,16.5] |
| *Chronic renal failure* | 1.4 [1.4,1.5] | 1.04 [1.02,1.05] | 7.1 [7.0,7.2] | 5.17 [5.13,5.21] | 11.9 [11.7,12.0] | 9.1 [9.0,9.1] |
| *Acute renal failure* | 1.8 [1.8,1.9] | 1.19 [1.18,1.21] | 7.9 [7.8,8.0] | 6.13 [6.08,6.18] | 15.3 [15.0,15.5] | 12.3 [12.1,12.4] |
| Diabetes Mellitus | 0.8 [0.8,0.8] | 0.97 [0.96,0.99] | 4.4 [4.3,4.5] | 4.27 [4.23,4.31] | 7.9 [7.8,8.1] | 7.1 [7.0,7.1] |
| Dementia | 0.7 [0.7,0.7] | 0.78 [0.76,0.79] | 3.2 [3.1,3.3] | 3.75 [3.72,3.79] | 5.6 [5.5,5.7] | 6.5 [6.4,6.6] |
| *Vascular dementia* | 0.2 [0.2,0.2] | 0.20 [0.20,0.21] | 0.8 [0.8,0.9] | 1.01 [1.00,1.03] | 1.5 [1.5,1.6] | 1.8 [1.7,1.8] |
| Depression | 0.8 [0.8,0.9] | 0.94 [0.93,0.96] | 3.3 [3.3,3.4] | 4.21 [4.17,4.25] | 5.5 [5.4,5.6] | 6.9 [6.8,7.0] |
| Cancer | 1.7 [1.7,1.8] | 2.66 [2.63,2.68] | 7.3 [7.2,7.4] | 9.41 [9.35,9.46] | 11.5 [11.3,11.7] | 14.4 [14.3,14.5] |
| *Breast* | 0.1 [0.1,0.1] | 0.12 [0.12,0.13] | 0.4 [0.4,0.4] | 0.52 [0.51,0.54] | 0.6 [0.6,0.7] | 0.8 [0.8,0.9] |
| *Prostate* | 0.2 [0.2,0.2] | 0.42 [0.41,0.43] | 1.0 [0.9,1.0] | 1.60 [1.58,1.62] | 1.7 [1.6,1.8] | 2.5 [2.5,2.6] |
| *Lung* | 0.2 [0.2,0.2] | 0.31 [0.30,0.32] | 1.0 [1.0,1.0] | 1.07 [1.05,1.09] | 1.8 [1.7,1.8] | 1.7 [1.6,1.7] |
| *Colorectal* | 0.2 [0.2,0.2] | 0.28 [0.28,0.29] | 0.7 [0.7,0.8] | 0.91 [0.89,0.93] | 1.2 [1.1,1.3] | 1.4 [1.4,1.4] |
| All-cause mortality | 6.14 [6.07,6.21] | 4.60 [4.57,4.63] | 20.9 [20.9,21.1] | 17.1 [17.0,17.1] | 32.5 [32.3,32.8] | 27.1 [27.1,27.3] |

^a^Cumulative incidences are presented as percentage of cases expected to develop each outcome by each respective timepoint, and adjusted for non-linear age using restricted cubic spline functions, sex, calendar year and deprivation score - treating death without outcome as a competing risk. ^b^Individuals were matched according to single year of age, sex, month and year of hospital admission and NHS Trust using a 5:1 risk-set matching approach. ^c^Cases within the matched control cohort who went on to develop MI were censored at time of first MI, therefore estimates of subsequent MI for this cohort were not included. Abbreviations: CI – confidence interval; MI – myocardial infarction; NA – not applicable; NHS – national health service; SD – standard deviation.
