# Supplementary material for: Health outcomes after myocardial infarction: A population study of 56 million people in England
Source: PLoS Med. 2024 Feb 15;21(2):e1004343. doi: 10.1371/journal.pmed.1004343 (PMC10868847; doi:10.1371/journal.pmed.1004343)
Supplement: S7 Table — aNumbers at risk at 1, 5, and 9 years follow-up are equal for those in the main analyses and the sensitivity analyses by design. MI, myocardial infarction. (DOCX) [file pmed.1004343.s012.docx]

|  | **Study entry** | | **1 year follow up** | **5 years follow up** | **9 years follow up** |
| --- | --- | --- | --- | --- | --- |
|  | All post MI events | Events ≥ 2 months post MI | All post MI events^a^ | All post MI events^a^ | All post MI events^a^ |
| Subsequent MI | 433,361 | 433,361 | 329,644 | 138,189 | 2,810 |
| Heart failure | 433,361 | 351,558 | 276,962 | 119,077 | 2,455 |
| Atrial fibrillation | 433,361 | 373,023 | 288,819 | 122,488 | 2,512 |
| Cerebrovascular disease | 433,361 | 414,423 | 320,241 | 135,427 | 2,728 |
| *Stroke* | 433,361 | 423,971 | 328,314 | 140,735 | 2,877 |
| Peripheral arterial disease | 433,361 | 422,979 | 325,589 | 139,214 | 2,839 |
| *Aortic disease* | 433,361 | 427,726 | 330,046 | 142,652 | 2,954 |
| Severe bleeding | 433,361 | 404,684 | 305,980 | 125,236 | 2,480 |
| *Gastrointestinal bleeding* | 433,361 | 422,331 | 323,697 | 137,230 | 2,786 |
| Renal failure | 433,361 | 392,928 | 309,650 | 130,432 | 2,547 |
| *Chronic renal failure* | 433,361 | 413,831 | 319,581 | 134,808 | 2,679 |
| *Acute renal failure* | 433,361 | 406,294 | 320,018 | 137,457 | 2,766 |
| Diabetes Mellitus | 433,361 | 376,410 | 289,199 | 120,725 | 2,340 |
| Dementia | 433,361 | 423,846 | 329,038 | 142,183 | 2,941 |
| *Vascular dementia* | 433,361 | 431,615 | 333,368 | 144,492 | 3,001 |
| Depression | 433,361 | 420,255 | 322,247 | 137,447 | 2,789 |
| Cancer | 433,361 | 422,818 | 325,040 | 135,372 | 2,692 |
| *Breast* | 433,361 | 432,759 | 333,861 | 144,775 | 2,998 |
| *Prostate* | 433,361 | 430,314 | 332,012 | 143,348 | 2,972 |
| *Lung* | 433,361 | 432,061 | 333,650 | 144,846 | 3,007 |
| *Colorectal* | 433,361 | 432,281 | 333,223 | 144,396 | 2,994 |
| All-cause mortality | 433,361 | 384,655 | 334,606 | 145,492 | 3,032 |

^a^Numbers at risk at 1, 5 and 9 years follow up are equal for those in the main analyses and the sensitivity analyses by design. Abbreviations: MI – myocardial infarction.
