# Supplementary material for: Health outcomes after myocardial infarction: A population study of 56 million people in England
Source: PLoS Med. 2024 Feb 15;21(2):e1004343. doi: 10.1371/journal.pmed.1004343 (PMC10868847; doi:10.1371/journal.pmed.1004343)
Supplement: S8 Table — aExcess rate is presented as the aHR for each outcome comparing matched controls with individuals with MI and adjusted for nonlinear age using restricted cubic spline functions, sex, calendar year, and deprivation score—treating death without outcome as a competing risk. bIndividuals were matched according to single year of age, sex, month and year of hospital admission, and NHS Trust using a 5:1 risk-set matching approach. cCases within the matched control cohort who went on to develop MI were censored at time of first MI; therefore, estimates comparing the HR of subsequent MI between the MI cohort and matched controls are not applicable. aHR, adjusted hazard ratio; CI, confidence interval; MI, myocardial infarction; NA, not applicable. (DOCX) [file pmed.1004343.s013.docx]

|  | aHR^a^ [95% CI]; p-value |
| --- | --- |
|  |  |
| Subsequent MI | NA^c^ |
| Heart failure | 2.33 [2.30,2.36];p<0.001 |
| Atrial fibrillation | 1.21 [1.19,1.22];p<0.001 |
| Cerebrovascular disease | 1.19 [1.17,1.21];p<0.001 |
| *Stroke* | 1.13 [1.11,1.16];p<0.001 |
| Peripheral arterial disease | 1.67 [1.63,1.70];p<0.001 |
| *Aortic disease* | 1.41 [1.36,1.45];p<0.001 |
| Severe bleeding | 1.21 [1.20,1.23];p<0.001 |
| *Gastrointestinal bleeding* | 1.07 [1.06,1.09];p<0.001 |
| Renal failure | 1.39 [1.37,1.40];p<0.001 |
| *Chronic* | 1.54 [1.52,1.57];p<0.001 |
| *Acute* | 1.40 [1.38,1.42];p<0.001 |
| Diabetes mellitus | 1.07 [1.05,1.09];p<0.001 |
| Dementia | 0.96 [0.94,0.98];p<0.001 |
| *Vascular dementia* | 1.10 [1.07,1.14];p<0.001 |
| Depression | 0.87 [0.85,0.89];p<0.001 |
| Cancer | 0.85 [0.84,0.86];p<0.001 |
| *Breast* | 0.79 [0.75,0.83];p<0.001 |
| *Prostate* | 0.88 [0.84,0.91];p<0.001 |
| *Lung* | 1.00 [0.97,1.03];p=0.994 |
| *Colorectal* | 0.68 [0.66,0.71];p<0.001 |
| All-cause mortality | 0.92 [0.91,0.93];p<0.001 |

^a^Excess rate is presented as the hazard ratio (HR) for each outcome comparing matched controls with individuals with MI and adjusted for non-linear age using restricted cubic spline functions, sex, calendar year and deprivation score - treating death without outcome as a competing risk. ^b^Individuals were matched according to single year of age, sex, month and year of hospital admission and NHS Trust using a 5:1 risk-set matching approach. ^c^Cases within the matched control cohort who went on to develop MI were censored at time of first MI, therefore estimates comparing the HR of subsequent MI between the MI cohort and matched controls are not applicable. **Abbreviations** CI - confidence interval; HR – hazard ratio; MI – myocardial infarction; NA – not applicable.
