# Supplementary figures and images for: Health outcomes after myocardial infarction: A population study of 56 million people in England
Source: PLoS Med. 2024 Feb 15;21(2):e1004343. doi: 10.1371/journal.pmed.1004343 (PMC10868847; doi:10.1371/journal.pmed.1004343)

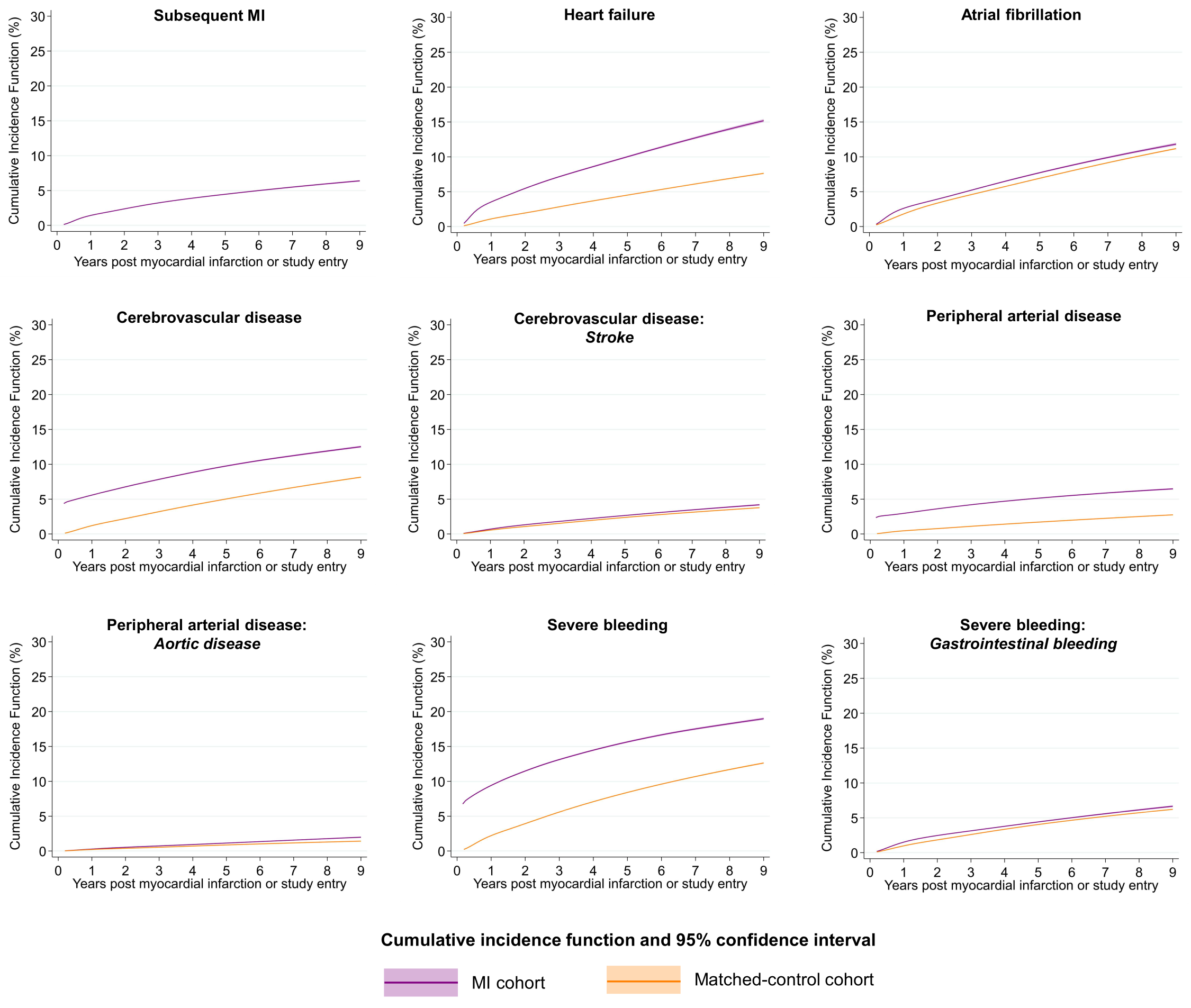

Supplement: S1 Fig — aCalculated according to the standardised CIF, treating death without outcome as a competing risk and adjusted for nonlinear age using restricted cubic spline functions, sex, calendar year, and deprivation score. bIndividuals were matched according to single year of age, sex, month and year of hospital admission, and NHS Trust using a 5:1 risk-set matching approach. CIF, cumulative incidence function; MI, myocardial infarction; NHS, National Health Service. (TIF) [file pmed.1004343.s014.tif]

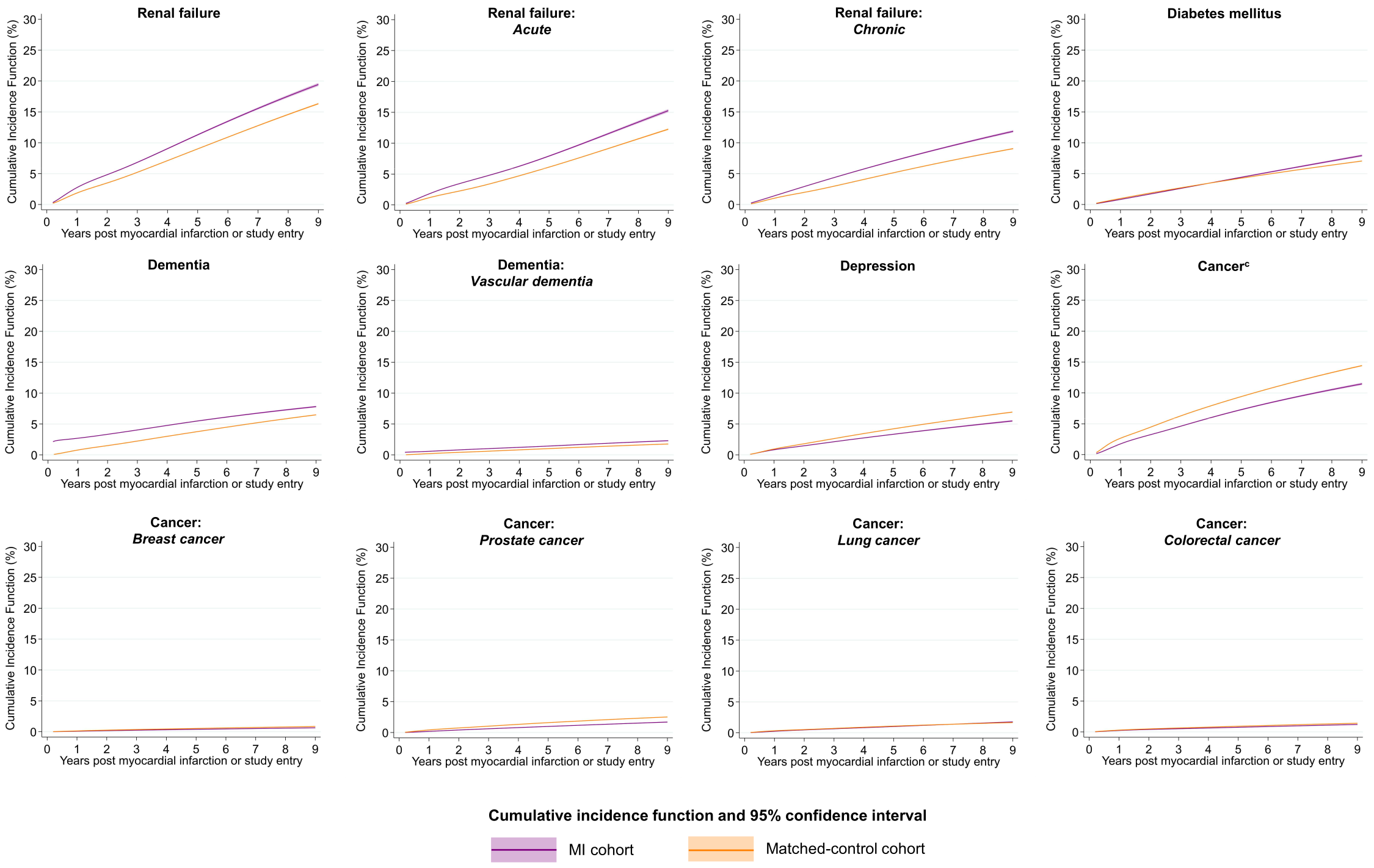

Supplement: S2 Fig — aCalculated according to the standardised CIF, treating death without outcome as a competing risk and adjusted for nonlinear age using restricted cubic spline functions, sex, calendar year, and deprivation score. bIndividuals were matched according to single year of age, sex, month and year of hospital admission, and NHS Trust using a 5:1 risk-set matching approach. cIncludes all cancer types (ICD10 codes C00–C97), i.e., this category is not restricted to the sum of breast, prostate, lung, and colorectal cancer). CIF, cumulative incidence function; MI, myocardial infarction; NHS, National Health Service. (TIF) [file pmed.1004343.s015.tif]

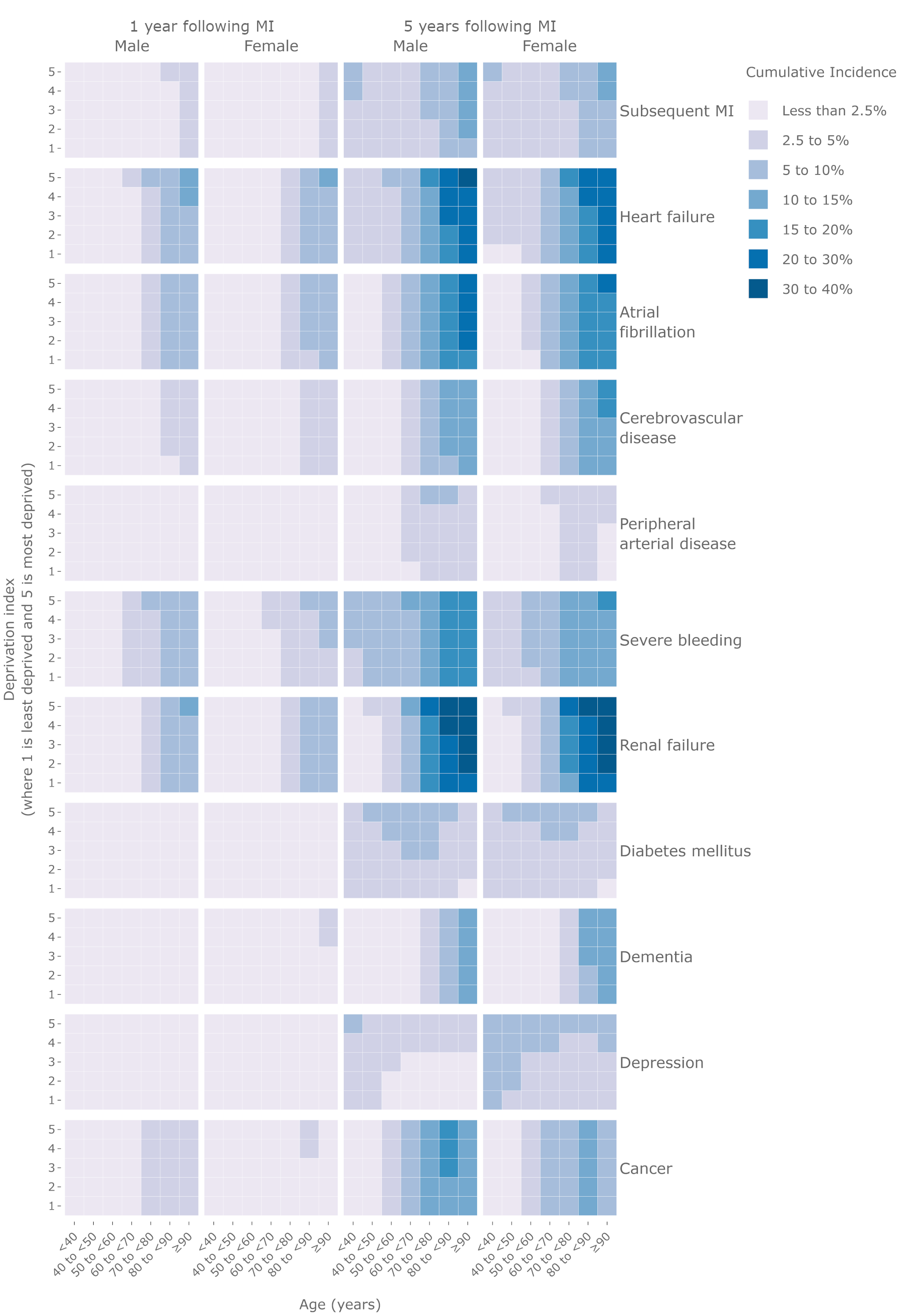

Supplement: S3 Fig — aCalculated according to the standardised CIF, treating death without outcome as a competing risk and adjusted for nonlinear age using restricted cubic spline functions, sex, calendar year, deprivation score, and receipt of invasive coronary angiography, percutaneous coronary intervention, or coronary artery bypass graft. bDeprivation is measured using the IMD where 1 indicates those in the least deprived quintile, and 5 indicates those in the most deprived quintile. Interactive version of these data are provided (https://multimorbidity-research-leeds.github.io/research-resources). CIF, cumulative incidence function; IMD, Index of Multiple Deprivation; MI, myocardial infarction. (TIF) [file pmed.1004343.s016.tif]

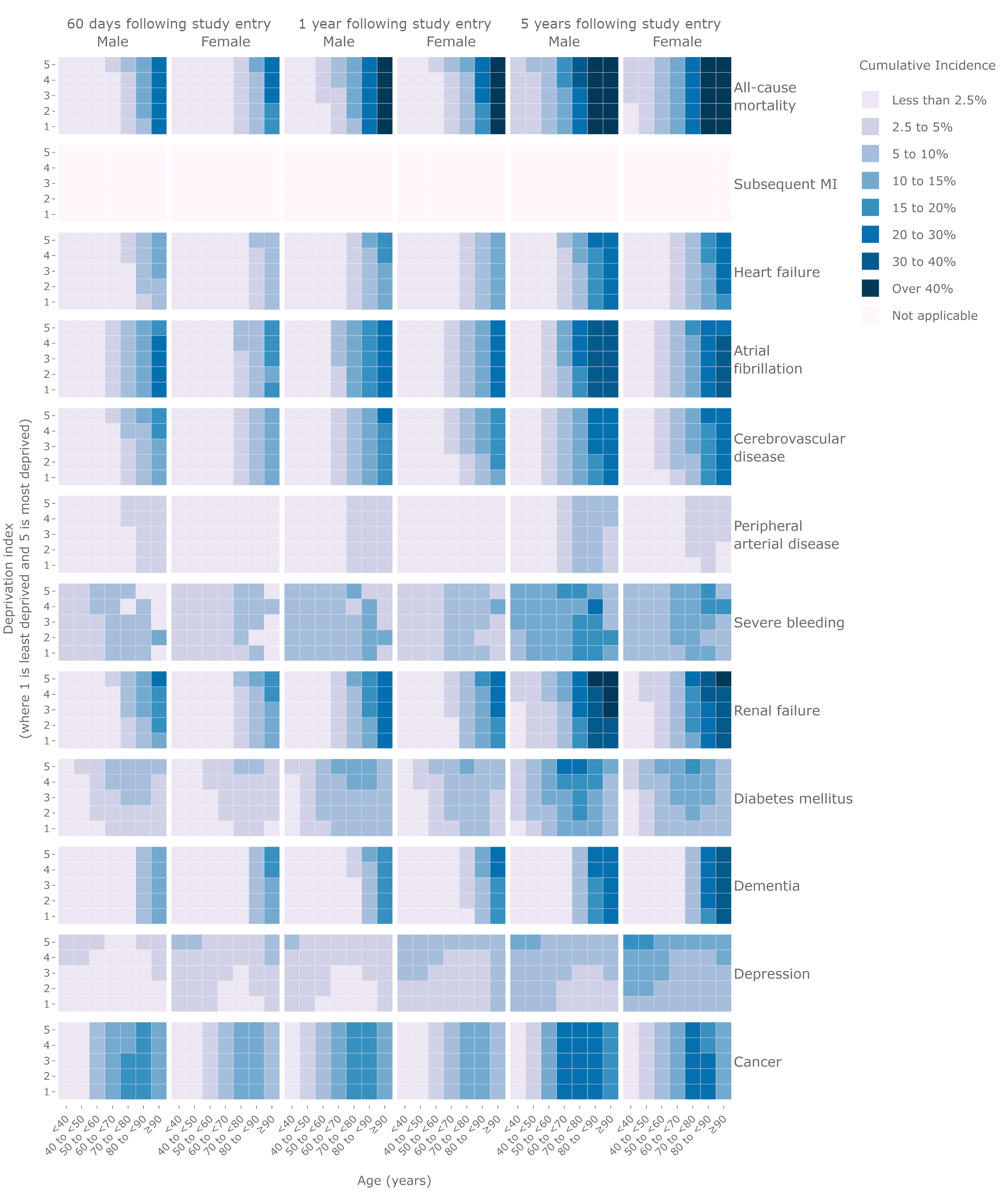

Supplement: S4 Fig — aCalculated according to the standardised CIF, treating death without outcome as a competing risk and adjusted for nonlinear age using restricted cubic spline functions, sex, calendar year, and deprivation score. bDeprivation is measured using the IMD where 1 indicates those in the least deprived fifth, and 5 indicates those in the most deprived fifth. Interactive version of these data are provided (https://multimorbidity-research-leeds.github.io/research-resources). CIF, cumulative incidence function; IMD, Index of Multiple Deprivation; MI, myocardial infarction. (TIF) [file pmed.1004343.s017.tif]

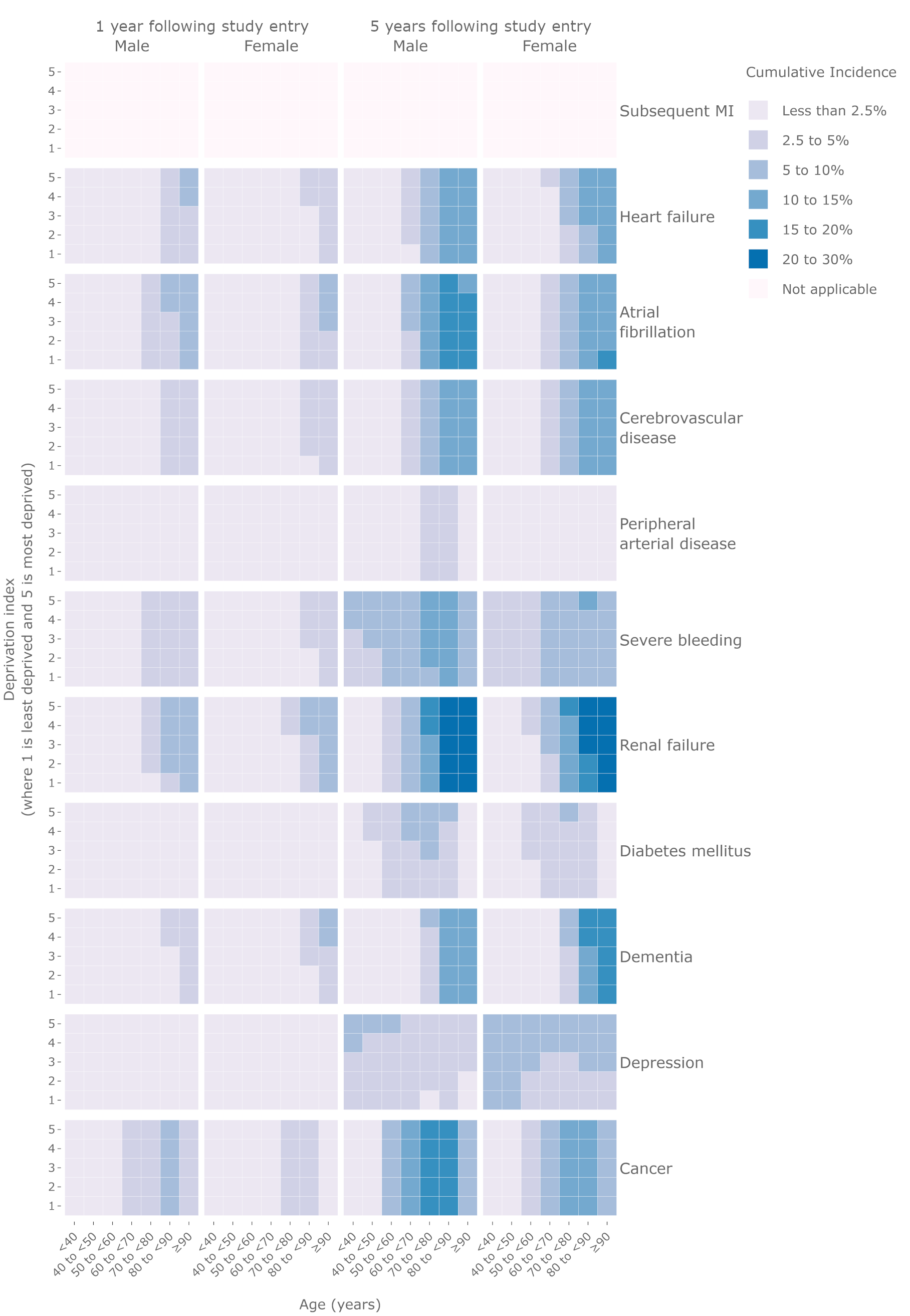

Supplement: S5 Fig — aCalculated according to the standardised CIF, treating death without outcome as a competing risk and adjusted for nonlinear age using restricted cubic spline functions, sex, calendar year, and deprivation score. bDeprivation is measured using the IMD where 1 indicates those in the least deprived fifth, and 5 indicates those in the most deprived fifth. Interactive version of these data are provided (https://multimorbidity-research-leeds.github.io/research-resources). CIF, cumulative incidence function; IMD, Index of Multiple Deprivation; MI, myocardial infarction. (TIF) [file pmed.1004343.s018.tif]
