# Supplementary material for: Health outcomes after myocardial infarction: A population study of 56 million people in England
Source: PLoS Med. 2024 Feb 15;21(2):e1004343. doi: 10.1371/journal.pmed.1004343 (PMC10868847; doi:10.1371/journal.pmed.1004343)
Supplement: Attachment — Submitted filename: PLOS Medicine 08-2023.docx [file pmed.1004343.s019.docx]

General comments:

1. Paper is well written, clear and succinct.
2. I think that it addresses an interesting question using appropriate data and methods.
3. I’m a bit unclear how the authors envision this work facilitating shared decision making in a pragmatic sense; it might be worth enhancing this topic in the discussion section. An alternative approach would be to simply eliminate the mention of SDM as a motivation for the study, though I would favor an approach of strengthening the discussion of SDM rather than removal.

Specific comments:

4. Methods: It isn’t clear how secular trends are accounted for in the analysis and how these could influence results.
   a. For example there have been changes over time diagnosis of AMI, most notably the widespread implementation of hsTrop testing. Likewise, I would imagine that treatment of AMI in the UK has evolved over time which would be expected to change rates of adverse outcomes.
   b. There also are secular trends in coding practices that are likely occurring absent changes in underlying disease incidence rates; this might be particularly relevant to the number of diagnoses (lines 169-170). It would be useful to consider these. See: <https://pubmed.ncbi.nlm.nih.gov/25341547/>
   c. It would be interesting to see if the study results are similar if you were to look specifically with patients hospitalized with AMI during the early years (2008-2009) and later years (2015-2017)? This comment is particularly germane to the results pictured in Figure 3. While your statistical models (lines 179-183) may in certain ways account for the time-varying nature of certain data elements, I am not entirely certain whether, for example, inclusion of calendar year and a time varying covariate for each cohort would fully suffice. Perhaps? Perhaps not?
5. Methods: I would be interested in knowing if the incidence of outcomes differs for patients who were hospitalized for STEMI vs NSTEMI.
6. Methods: did you limit AMI identification to patients with AMI listed in the primary diagnosis code field? I’m thinking yes, but don’t see that explicitly mentioned (lines 144-155).
7. Methods: How did you handle patients without any “look back” data, such as a patient who newly immigrated to the UK and thus you would not have information on a previous MI? In such cases, how would you determine the “1^st^ occurrence” (lines 150-153)?
8. Methods, Lines 158-166. Given the longitudinal nature of the data, could a patient exist in both the MI and non-MI cohorts? My sense is yes (for example if a patient had not had an AMI in 2010, but subsequently was hospitalized with AMI in 2015.
9. Methods, Patient and Public Involvement: This section is quite outstanding. Really terrific and thoughtful.
10. Results, line 249: Can you please clarify if there were 18,343,361 codes for MI….or if there were 18,343,361 diagnosis codes for all conditions amongst patients hospitalized with MI? My sense is the later and that this represents all codes.
11. Discussion, lines 327-330: is it possible that the lower incidence of cancer reflects reduced screening of patients who have had AMI?
12. Discussion: consider adding discussion about how study results might be incorporated into SMDM.
13. Tables/Figures are excellent.

Peter Cram, MD MBA

Professor of Medicine

University of Texas, Galveston
