# Supplementary material for: Health outcomes after myocardial infarction: A population study of 56 million people in England
Source: PLoS Med. 2024 Feb 15;21(2):e1004343. doi: 10.1371/journal.pmed.1004343 (PMC10868847; doi:10.1371/journal.pmed.1004343)
Supplement: Attachment — Submitted filename: Response to Peer Review and Editorial Comments.docx [file pmed.1004343.s020.docx]

Dear editorial team and reviewers,

Many thanks for the time taken to provide detailed and thoughtful reviews of our manuscript. We provide herewith a thorough response to each of the reviewers’ comments which we have numbered for ease of cross referencing. Our response comments are provided in **bold blue**. We have expanded our introduction and discussion substantially, adjusted for the use of an invasive coronary strategy in our analyses of the MI cohort, address concerns over case ascertainment of health outcomes supported by existing literature, compare our findings of the reduced risk of cancer and no difference for dementia following MI to previous work and highlight more clearly the unique contributions of this research. Moreover, we address the general editorial comments and the comments made by the Editor in Chief in detail (see pages 18-19 and 19-28 of this response respectively). Finally – we provide detailed references in support of our rebuttal on pages 29-31.

Yours sincerely,

Dr Marlous Hall (she/her), on behalf of the co-authors.

**Comments from the reviewers**

Reviewer #1: Health outcomes after myocardial infarction: a population study of 56 million people in England In this manuscript A/prof Hall and co-workers report on the post- myocardial infarction (MI) outcomes in a very large nationwide cohort UK. From about 34 millions UK adults contributing to hospital admissions (about 146 millions) from 2008 to 2017 and who were included, about 443K had MI. The incidence of a range of outcomes (fatal and non-fatal) both cardiovascular and non-cardiovascular was high, with many exceeding those observed in a matched non-MI sub-cohort.

1. The study is novel in the sense that it goes beyond what previous studies have achieved, and is based in a large sample, therefore translating into stable estimates of the outcomes considered; and as such has a potential to add to the existing body of knowledge on the outcomes of people experiencing an MI, a leading cause of deaths worldwide. The authors should also be commended on the application of robust analytic methods.

**Many thanks for your kind comments.**

The study also has some limitations or points to be addressed to get the right message across.

2. The authors are suggesting that the only criteria they used to exclude participants with prevalent outcome of interest at baseline was prior hospitalization for the outcome of interest (see line 257-359). Should this be the case, then it is rather a weak criteria and many people diagnosed with the outcome of interest during follow would actually be people who had the outcome already at baseline and that had not yet caused a hospitalization in the past; translating into an inflation of the incidence. Taking the case of diabetes for illustration, at the average age of participants at inclusion (67 years), diabetes prevalence would be in the region of 10-20% in the general population and likely even higher in those with MI.

One query to the authors would be to know if the electronic database didn't include other indicators of existing comorbidities, they use to exclude those with known outcomes at baseline to improve the estimates of their incident outcomes during follow-up.

**The reviewer is correct in that our exclusion criteria are based on previous hospitalisations of each outcome, as we do not have access to data from primary care for this study. However – the application of these exclusions were not limited to within our study period, but extend back through the entire history of the HES data warehouse (to 1^st^ April 1997). Our look-back period therefore covers 10 years and 9 months in total.**

**Our coding lists for identifying existing co-morbidities are extensive to capture pre-existing comorbidities. In particular, for diabetes we used the following extensive set of codes to identify pre-existing diabetes (these data are presented in aggregate form to 3-digit ICD10 codes in S2 Table), but expanded here for the reviewers’ information:**

| **Diabetes mellitus** | |
| --- | --- |
| **E10.0** | **Type 1 diabetes mellitus, with coma** |
| **E10.1** | **Type 1 diabetes mellitus, with ketoacidosis** |
| **E10.2** | **Type 1 diabetes mellitus, with renal complications** |
| **E10.3** | **Type 1 diabetes mellitus, with ophthalmic complications** |
| **E10.4** | **Type 1 diabetes mellitus, with neurological complications** |
| **E10.5** | **Type 1 diabetes mellitus, with peripheral circulatory complications** |
| **E10.6** | **Type 1 diabetes mellitus, with other specified complications** |
| **E10.7** | **Type 1 diabetes mellitus, with multiple complications** |
| **E10.8** | **Type 1 diabetes mellitus, with unspecified complications** |
| **E10.9** | **Type 1 diabetes mellitus, without complications** |
| **E11.0** | **Type 2 diabetes mellitus, with coma** |
| **E11.1** | **Type 2 diabetes mellitus, with ketoacidosis** |
| **E11.2** | **Type 2 diabetes mellitus, with renal complications** |
| **E11.3** | **Type 2 diabetes mellitus, with ophthalmic complications** |
| **E11.4** | **Type 2 diabetes mellitus, with neurological complications** |
| **E11.5** | **Type 2 diabetes mellitus, with peripheral circulatory complications** |
| **E11.6** | **Type 2 diabetes mellitus, with other specified complications** |
| **E11.7** | **Type 2 diabetes mellitus, with multiple complications** |
| **E11.8** | **Type 2 diabetes mellitus, with unspecified complications** |
| **E11.9** | **Type 2 diabetes mellitus, without complications** |
| **E12.0** | **Malnutrition—related diabetes mellitus, with coma** |
| **E12.1** | **Malnutrition—related diabetes mellitus, with ketoacidosis** |
| **E12.2** | **Malnutrition—related diabetes mellitus, with renal complications** |
| **E12.3** | **Malnutrition - related diabetes mellitus, with ophthalmic complications** |
| **E12.4** | **Malnutrition—related diabetes mellitus, with neurological complications** |
| **E12.5** | **Malnutrition - related diabetes mellitus, with peripheral circulatory complications** |
| **E12.6** | **Malnutrition - related diabetes mellitus, with other specified complications** |
| **E12.7** | **Malnutrition - related diabetes mellitus, with multiple complications** |
| **E12.8** | **Malnutrition - related diabetes mellitus, with unspecified complications** |
| **E12.9** | **Malnutrition - related diabetes mellitus, without complications** |
| **E13.0** | **Other specified diabetes mellitus, with coma** |
| **E13.1** | **Other specified diabetes mellitus, with ketoacidosis** |
| **E13.2** | **Other specified diabetes mellitus, with renal complications** |
| **E13.3** | **Other specified diabetes mellitus, with ophthalmic complications** |
| **E13.4** | **Other specified diabetes mellitus, with neurological complications** |
| **E13.5** | **Other specified diabetes mellitus, with peripheral circulatory complications** |
| **E13.6** | **Other specified diabetes mellitus, with other specified complications** |
| **E13.7** | **Other specified diabetes mellitus, with multiple complications** |
| **E13.8** | **Other specified diabetes mellitus, with unspecified complications** |
| **E13.9** | **Other specified diabetes mellitus, without complications** |
| **E14.0** | **Unspecified diabetes mellitus, with coma** |
| **E14.1** | **Unspecified diabetes mellitus, with ketoacidosis** |
| **E14.2** | **Unspecified diabetes mellitus, with renal complications** |
| **E14.3** | **Unspecified diabetes mellitus, with ophthalmic complications** |
| **E14.4** | **Unspecified diabetes mellitus, with neurological complications** |
| **E14.5** | **Unspecified diabetes mellitus, with peripheral circulatory complica ions** |
| **E14.6** | **Unspecified diabetes mellitus, with other specified complications** |
| **E14.7** | **Unspecified diabetes mellitus, with multiple complications** |
| **E14.8** | **Unspecified diabetes mellitus, with unspecified complications** |
| **E14.9** | **Unspecified diabetes mellitus, without complications** |
| **G59.0** | **Diabetic mononeuropathy** |
| **G63.2** | **Diabetic polyneuropathy** |
| **H28.0** | **Diabetic cataract** |
| **H36.0** | **Diabetic retinopathy** |
| **M14.2** | **Diabetic arthropathy** |
| **N08.3** | **Glomerular disorders in diabetes mellitus** |

**We acknowledge in the discussion that some events observed shortly after study entry may include existing diagnoses made outside of secondary care. We therefore provide a thorough, complementary sensitivity analysis in which we delay follow-up time to two months after index MI or study entry for matched controls – whilst also highlighting that some of these early events remain important and significant in that they reflect conditions likely diagnosed shortly after MI due to screening for risk factors, sequelae or complications.**

**We further take the reviewers point that if pre-existing conditions are observed for the first time during the follow up period only, this could indeed cause inflation of the post MI incidence. A recent study which assessed the accuracy of recording of co-morbidities in HES data[1] shows the high sensitivity and specificity in HES admitted patient care data for co-morbidities and specifically for diabetes (97.7% sensitivity and 96.1 specificity). This has been included in the discussion, which reads as follows:**

***“We did not have access to diagnoses made outside of secondary care, which may have resulted in some individuals with pre-existing co-morbidities being missed during application of our exclusion criteria. Whilst the potential implication of this is an inflation of incidence rates following MI – we have mitigated against this as far as possible by making use of a substantial look-back period (in excess of ten years) for pre-existing conditions and capturing conditions via comprehensive code lists******…”***

***“….Furthermore - HES data have been shown to have high sensitivity and specificity of co-morbidity recording, in particular for diabetes, with 97.7% sensitivity and 96.1% specificity[1] and*** ***whilst there is evidence of delayed dementia diagnoses in HES compared with primary care of 1.6 years, ascertainment of dementia overall is also high (85%)[2].*** ***To mitigate against the potential bias of undercounting of pre-existing co-morbidities, we present a full sensitivity analyses in which follow up was delayed to 60 days after index MI or study entry, whilst acknowledging that our primary findings are substantiated by other studies reporting high rates of re-hospitalisation within 30-days following MI [3], which reflect conditions likely diagnosed shortly after MI due to screening for risk factors (e.g., diabetes) and sequelae (e.g., heart failure) or due to complications (e.g., bleeding).”***

3. With vascular outcomes (expectedly) dominating the outcomes during follow-up, it is a severe limitation that the description of the baseline profile of the participants does not include indicators of their vascular risk profiles: smoking, hypertension, dyslipidemia, diabetes etc. Should this data be available in the database, then it should be presented with related treatments.

**Many thanks for your helpful suggestion. We now include data on cardiovascular risk factors (including history of smoking, hypertension and dyslipidaemia), identified using ICD-10 codes (S2 Table). In addition to the suggested risk factors, we also included measures of alcohol excess and obesity. We do not include baseline diabetes data given this was one of our *a priori* outcomes – and therefore individuals with prior diabetes were excluded at source. The additional data have been added to the manuscript to further describe our cohort (Table 1) with added rows as follows:**

| **Cardiovascular risk factors (N, %)** | **Non MI** | **MI** | **Matched Controls** |
| --- | --- | --- | --- |
| **Hypertension** | **6,839,977/33,429,669 (20.5%)** | **211,386/433,361 (48.8%)** | **738,075/2,001,310 (36.9%)** |
| **Dyslipidaemia** | **2,360,150/33,429,669 (7.1%)** | **127,921/433,361 (29.5%)** | **237,305/2,001,310 (11.9%)** |
| **Obesity** | **1,541,944/33,429,669 (4.6%)** | **25,706/433,361 (5.9%)** | **74,851/2,001,310 (3.7%)** |
| **Tobacco smoking** | **3,974,449/33,429,669 (11.9%)** | **124,773/433,361 (28.8%)** | **228,557/2,001,310 (11.4%)** |
| **Alcohol excess** | **1,316,047/33,429,669 (3.9%)** | **17,031/433,361 (3.9%)** | **92,905/2,001,310 (4.6%)** |

**There are, however, limitations to these newly included baseline risk data which we acknowledge in the discussion. Given that data minimisation standards precluded us from holding the historical HES records in-house – the description of baseline risk for patients at study entry only covers that observed in the study period. Further, we expect that ascertainment of smoking status, obesity and alcohol excess in HES may be low. Nonetheless, we agree with the reviewer that these data provide informative descriptions about the cohort.**

**This has been reflected in the manuscript discussion as follows :**

**“*****Our cohort descriptives include a summary of baseline cardiovascular risk – however the look-back period for this specific information was limited to within the study period only. In addition, we acknowledge the likely under-reporting of lifestyle related risk factors including tobacco smoking, alcohol excess and obesity within hospitilisation records – and interpret these data as representing the extremes of the population distribution.”***

3)	Linked to #2, the outcomes of patients with MI are assumed to be influenced by the acute treatment as well as implementation of secondary prevention therapies following the events. This paper cannot afford not to report on acute treatment of the MI (revascularization) and implementation/uptake of secondary prevention therapies at baseline; then testing their effect on the incidence of the outcomes (vascular in particular during follow-up). It is only after achieving this that they can speculate as they have done on lines 311-313 on the importance of intensifying secondary prevention.

**HES admitted patient care data does not capture information with regards to secondary preventative medication, and there is currently no national individual-level hospital prescribing database for England which could be linked to HES data without patient consent for research purposes[4]. We have been more explicit in our manuscript discussion to highlight that we did not have data on secondary preventative medication in several places in the discussion as follows:**

**“Given that we did not assess the impact of specific medications within this study, we are unable to state whether aspirin contributed to the reduced risk of cancer observed.”**

**and**

**“HES admitted patient care data does not capture information with regards to secondary preventative medication, and there is currently no national individual-level hospital prescribing database for England which can be linked without consent for research purposes[4]. As such we were unable to quantify the impact of medication on the post MI incidence of health outcomes observed in this study. We do however adjust our analyses of the MI cohort for receipt of invasive MI management including receipt of angiography, coronary percutaneous intervention or coronary artery bypass graft.”**

**Nonetheless, we have now included data on the invasive coronary strategy for MI in Table 1, and additionally adjust for this in our analyses. We have done so by capturing individuals coded for invasive coronary angiography, percutaneous coronary intervention (PCI) or coronary artery bypass graft (CABG) using OPCS4.5 procedure codes which are provided in full in S2 Table. There were 319,439 (73.7%) individuals who received invasive coronary angiography, PCI or CABG for their index MI (Table 1).**

**Please note that we adjusted for the receipt of an invasive coronary strategy in our analyses of age, sex, deprivation and follow-up time specific risk charts for the MI cohort. We did not adjust for these data in analyses which compared the MI cohort and matched control cohort directly – given the treatment is only applicable to those in the MI cohort, such an adjustment would incorrectly average and dilute the treatment effect between both the MI and matched control cohorts.**

Reviewer #2:

This article is on the incidence of a range of health outcome following an MI, as determined from record linkage of hospitalisations in England. The report uses a large data set and the survival analysis generally seems appropriate, but there are nonetheless some areas that are not entirely clear:

1. The event rates following on from MI may be informative, but the control group against which outcomes for MI is compared involves all other participants entering hospital for any reason. It's therefore not surprising that there are differences compared to the MI group, but given the wide variety and severity of other hospitalisations it's difficult to interpret what this means in practice. Similar results could presumably be seen with any more severe hospitalisation event like an MI against a collection of other events including those which may be much less serious. Although there is also a matched cohort, using "age, sex, month and year of hospital admission, and NHS Trust", this would also not account for the different severity of admissions and what meaningful interpretation can be made against the MI specific group.

**Thank you for raising this important point - in part we agree. However, our control group includes all ‘non-MI’s’ which will include the full range of admissions to hospital and is not limited to those which are less serious. In effect, admission to hospital in itself is a reflection of serious (more severe) illness. We know that MI is a medical emergency – and we know it can also be a catalyst for future health outcomes, but here we wanted to quantify and evidence that using large scale data. We have added further discussion on this to our manuscript. In addition – we believe it is important to show the relative incidence of the MI cohort compared to a matched control cohort in order to specifically highlight the *excess* burden over and above that expected for individuals without MI; which we feel is achieved appropriately by comparing nationally to a matched cohort derived from all hospitalised non-MI individuals. We additionally provide data on the absolute incidence of major health outcomes for the MI cohort specifically – which can be used directly by healthcare providers and patients to assess their likely course of future health outcomes. We believe that providing both the relative and absolute incidence data ensures our study is unique and important to that end.**

**The following text has been added to the discussion:**

***“…..Nonetheless, conditions captured in this study can be framed as clinically, epidemiologically and economically important given that admission to hospital is a key clinical event, often signifying later-stage, more severe or complex disease. Whilst we did not adjust for severity of hospitalisation in our analyses, our comparator cohort captures the full range of admissions in that it includes all individuals without MI and was not limited to less severe disease.”***

2. The explanation for why there is such a substantial decrease in risk of cancers is odd, as if aspirin played such a significant role then presumably it could/would be widely used to prevent a huge number of events throughout the general population. Potentially it could have something to do with the inclusion/exclusion criteria of events for the study, but given the scale of the finding it would be useful to have this commented on further as will also place into context the strength of association for where MI increases risk.

**We have expanded our discussion of the reduced risk of cancer, comparing our results in detail to two large studies showing similar results for breast and prostate cancer, but conflicting results for cancer overall, lung and colorectal cancer. We have also tempered the discussion around aspirin (as some of the nuance of our initially intended discussion was lost when reducing the word limit in a previous iteration). The updated discussion now reads as follows:**

***“Following MI, our study found a lower incidence of cancer overall, as well as for breast, colorectal, lung and prostate cancer compared with matched controls.*** ***Two existing large-scale studies have also described a significantly reduced risk of breast and prostate cancer among 122,275 individuals with MI[5] and among cardiovascular disease more broadly[6]. Our study builds on this, providing the demographic-specific values of absolute risk for key cancer subgroups and cancers overall. We also show a reduced risk of colorectal and lung cancer, and for all cancers combined. These latter findings are inconsistent with previously reported data showing an increased incidence of lung cancer following MI[5], a non-significant difference in colorectal cancer following MI compared with matched controls[5] and an increased risk of lung, colorectal and all cancers combined for individuals with cardiovascular disease[6]. The reason for conflicting evidence is unclear but may be explained by different population demographics (including a smaller and younger population of MI[5] and a broader cardiovascular population[6]). Moreover, competing risk of death was not accounted for[6], which is likely to have had a marked impact on findings given the high early mortality rate observed for individuals with cardiovascular disease. The mechanisms underpinning a reduced risk of all cancer and for specific subtypes following MI remain unclear and warrant further investigation.*** ***Whilst trial data have assessed the role of aspirin in the onset, progression and mortality of specific cancer subtypes,[7-9] broader evidence for its beneficial effect remains unclear.[10] Given that we did not assess the impact of specific medications within this study, we are unable to state whether aspirin contributed to the reduced risk of cancer observed. There is some evidence of reduced rates of screening for cancer amongst individuals with cardiovascular disease which could explain the findings[11]. However, there may also be a surveillance bias acting in the opposite direction. Finally, given that our study focussed specifically on the development of new health outcomes following MI, we included only individuals living long enough cancer-free to develop MI before cancer and this may further explain our findings.”***

3. The analysis as described in line 182 suggests that there is a "time varying covariate for cohort", but it isn't entirely clear what is meant by this and if it is actually a time-updated survival analysis being used. If the cohorts relate to the MI and matched groups then not sure how or why these would change over time following initial classification. If the cohorts refer to the combinations of age, sex and socioeconomic deprivation then it is possible that age or deprivation can change over time, but with the follow up of at most 9 years then would presumably not be much change in these.

**Apologies for the lack of clarity here – we have updated our terminology to more accurately reflect that this is a *time-dependent effect*. The cohort variable does indeed refer to a binary variable for MI versus matched controls. Its’ inclusion as a time-dependent effect however has a different interpretation. It is not the variable itself which varies with time, rather, we are modelling non-proportional hazards by allowing the hazard ratio between MI and matched controls to vary as a function of follow up time. We have included this in our model on the assumption that the risk of outcomes between the MI and matched control population may change over follow up time (i.e. there may be an initial greater risk in the short term following MI, with the potential for this risk to lower to ‘background population’ levels in the long term).**

**In practice, this modelling approach means we were able to obtain standardised estimates of hazard as a function of continuous follow up time, presented in our study in the form of cumulative incidence functions shown in Figures 3A and 3B. Importantly – time-dependent effects allow us to observe scenarios such as that for depression - in which the cumulative incidence is initially higher following MI than matched controls, but as follow up time increases, the cumulative incidence following MI is lower than that of matched controls. We have updated our terminology from time-varying covariate to *time-dependent effect* throughout to more accurately reflect our approach, and we have further clarified the methods as follows:**

***“…and additionally including a time-dependent effect for cohort (MI versus matched controls) to reflect the potential difference in cumulative incidence between cohorts over follow-up time.”***

4. The conclusions in both the abstract and main text that "four in ten [patients] die within ten years" following an MI should probably be placed in context of the age of the population (~67) and that is only slightly higher (39.6%) compared to the age matched hospital population (37.3%), as would seem logical that deaths will occur within an older population irrespective of having an MI. Also while technically true, the abstract refers to "within ten years" whereas table 1 suggests the total follow up period is "9 years".

**Thanks for highlighting this. The reviewer is correct that the difference in the crude, unadjusted Kaplan-Meier estimate of survival over the follow-up time is marginally different (39.6% vs. 37.3% in the MI vs matched controls); the results which we referred to in the abstract and main text are based on the adjusted cumulative incidence function at 9 years which are 37.8%, [95% CI 37.6%, 37.9%] amongst MI vs 35.3% [95% CI 35.3%,35.5%] amongst matched controls by the end of the follow up period (S5 Table). Whilst this was statistically significant - we have now quantified this conclusion with direct comparison in the manuscript text as follows:**

**“We found that up to a third of patients with MI developed heart failure, 7% had further MI and 38% died within 9 years (compared with 35% of deaths in matched individuals without MI).”**

5. Numbers at risk in table S6 drop quickly from the initial totals, with ~25% not included by year 1, 66% by year 5 and ~99% by the full 9 years of potential follow up between 2008-2017. The cumulative incidence within figure 3 also suggests that majority of excess risk is within first year post hospitalisation and then follows reasonably consistent pattern between groups after this. Within the article not much comment seems to be made on this, but this affects the interpretation of long-term MI outcomes given most of the event data actually seems to relate to occurrences within a year of primary MI.

**Thanks for raising this important point. We recognise that we have an accelerated failure rate for all-cause mortality as well as the non-fatal health outcomes; and that numbers at risk decline over follow up time due to the nature of observational study designs such as these. We present these data clearly in S7 Table (previously Table S6) and this is also one of the reasons for which we have presented risk charts at 60 days, 1 year and 5 years of follow up. We have now highlighted that the numbers at risk decline in the discussion, but also note that despite this drop off, our numbers at risk are still large at 5-years (in excess of 110,000 individuals) and 9 years (in excess of 2400) for all outcomes. The following has been added to the manuscript discussion:**

***“******We acknowledge that numbers at risk decline rapidly over the follow up period – implying that the largest proportion of data relate to outcomes within one year of the MI. Whilst we present risk charts separately at 60 days, 1 year and 5 years of follow up in recognition of this – our study was sufficiently large to provide statistically robust estimates of long-term outcomes given that numbers at risk exceeded 110,000 individuals per outcome with MI at 5 years and 2,400 individuals per outcome with MI at 9 years.”***

6. I'm not sure if the analysis with events excluded if they occur within two months is technically a landmark analysis, as it isn't being used to classify "responders" after a certain time. Instead it is just excluding from consideration/totals all events in the first two months after hospitalisation. There is some justification for doing this as described by the authors, but is then a bit confusing that this is referred to throughout but only appears in supplementary analysis as main results include events within 30 days.

**Apologies for the lack of clarity. We have now relabelled these analyses more appropriately as a sensitivity analysis and provided more clarity on our definition / exclusion criteria. Rather than simply exclude events within two months of MI – we limited our sensitivity analyses to those still at risk at 2 months following MI (i.e we restricted our follow up period from a minimum of two months onwards). We have opted to retain these results in the supplement as they are there to aid discussion and further interpretation of our work – without overloading the already dense main results of the paper. We have clarified in the methods as follows:**

***“We conducted a sensitivity analysis for all non-fatal health outcomes in which follow up was restricted to two or more months following MI to assess the impact of potential bias from the high number of events observed shortly after study entry. This was a data driven decision (S2 Text).”***

7. In the matched group, it is stated that censoring is done at the time of any future MI. Given the large number of events associated with MI as seen with the first two months after primary event, this could potentially exclude some other events from the matched group and inflate the differences compared to the MI group. Even if it is considered necessary for some analysis, the numbers within the matched group with subsequent MI is presumably known and could be reported for comparison.

**To accurately compare the outcomes of individuals with MI to those without MI - it is necessary to censor the follow up period at the time of subsequent MI. Matched controls at the time of study entry (when they do not have MI) are at risk of developing study outcomes – and are therefore included. Once they develop MI – they are no longer at risk of developing the study outcome as a matched control (and are therefore censored). However – such individuals are then at risk of developing outcomes following the censored MI event – and therefore contribute that portion of their follow up data to the MI cohort. Whilst this is conceptually a little tricky, and often either ignored or not reported, it forms part of risk-set matching good practice guidelines. Contribution of these individuals to *both* MI and matched control cohorts should limit the concern for bias raised by the reviewer. The reason that individuals with MI in the future must be included in the matched control cohort in the first place is that any decision to exclude them from the risk set in advance would mean we are conditioning on an event in the future (i.e. at the time of study entry and prospective follow up we cannot condition on the future development of MI – as this would cause the fatal statistical flaw of immortal time bias).**

**We have updated our methods section to further clarify the approach:**

***“Due to the longitudinal nature of our data, and as per risk-set matching guidance, individuals who later went on to develop MI were censored at the date of MI for the control cohort, but contribute to both the MI and matched control cohorts in further analyses[12].“***

**Further – we provide the following information with regards to the number of matched controls who went on to develop MI and list these in the results section as follows:**

***“Of matched controls, 18,322 (0.92%) individuals went on to develop MI and therefore contribute data to both the MI and matched control cohorts.”***

7. Figure 1 suggests there are ~9.3 million duplicated records that get excluded. Although numbers will be large simply due to nature of study, and are also still left with large numbers after exclusion, the process behind what generated the duplicates may be relevant to note if it is simply an administrative process, or instead if this could represent any concerns/bias with the data.

**Duplicate HES episodes which contain the same data across HES fields including admission start and end dates, episode start and end dates, primary and secondary diagnoses codes and procedure codes are administrative duplications where incorrect/new entries have been created. This is a known issues with HES data coding and expected to arise in data extracts. As such, whilst there were a large number of duplicate episodes, we do not anticipate that this presents any bias in the results. The only impact of the exclusion of duplicate episodes on the analyses is to identify the appropriate denominator of total number of episodes per cohort as described in Table 1. Given our analyses focusses throughout on time to first event – entries of hospitalisation codes are only counted once at first occurrence – our analyses is therefore robust to the inclusion or exclusion of such duplicates, and no individual patients are removed as a result of duplicate hospitalisation records. We have included a description of the potential process behind these duplicates as a footnote to Figure 1 as follows:**

***“******Duplicate HES episodes which contain the same data across HES fields including admission start and end dates, episode start and end dates, primary and secondary diagnoses codes and procedure codes are administrative duplications where incorrect or new entries have been created”***

**The full discussion of duplicates as provided here in response to the reviewer has been added to S3 Text.**

Reviewer #3:

General comments:

1. Paper is well written, clear and succinct. I think that it addresses an interesting question using appropriate data and methods.

**Many thanks for your detailed review of our work.**

2. I’m a bit unclear how the authors envision this work facilitating shared decision making in a pragmatic sense; it might be worth enhancing this topic in the discussion section. An alternative approach would be to simply eliminate the mention of SDM as a motivation for the study, though I would favor an approach of strengthening the discussion of SDM rather than removal.

**We have updated and expanded the implications section of our discussion, which now also includes the following discussion on shared decision making:**

***“Finally - our sociodemographic stratified risk charts provide a crucial step in translating future health outcomes in a format directly accessible by healthcare professionals and patients to support informed and shared healthcare decision-making. Effective communication of the likely course of disease and risk of adverse long term outcomes between individuals and healthcare professionals have been shown to promote positive lifestyle changes, facilitate treatment compliance, and improve patient understanding and quality of life [13, 14].*** ***Informed by our PPIE activity, our graphics have been designed in an easy-to-use format and are published in a publicly accessible website. This provides healthcare professionals and patients with the necessary information to discuss relevant, demographic-specific, risks to effectively direct appropriate care and interventions.”***

Specific comments:

3. Methods: It isn’t clear how secular trends are accounted for in the analysis and how these could influence results.

For example there have been changes over time diagnosis of AMI, most notably the widespread implementation of hsTrop testing. Likewise, I would imagine that treatment of AMI in the UK has evolved over time which would be expected to change rates of adverse outcomes.

There also are secular trends in coding practices that are likely occurring absent changes in underlying disease incidence rates; this might be particularly relevant to the number of diagnoses (lines 169-170). It would be useful to consider these. See: <https://pubmed.ncbi.nlm.nih.gov/25341547/>

It would be interesting to see if the study results are similar if you were to look specifically with patients hospitalized with AMI during the early years (2008-2009) and later years (2015-2017)? This comment is particularly germane to the results pictured in Figure 3. While your statistical models (lines 179-183) may in certain ways account for the time-varying nature of certain data elements, I am not entirely certain whether, for example, inclusion of calendar year and a time varying covariate for each cohort would fully suffice. Perhaps? Perhaps not?

**We recognise that the universal definition of MI has changed over time. Given the nature of the ICD-10-based coding of MI, we are unable to reclassify newer events according to older definition or older events according to newer definitions. Moreover, we accept that these were the diagnoses made by clinician at that point in time. We do not plan to undertake a temporal tends analysis as part of this manuscript, which would change the remit and direction of the purpose of the research as well as overload an already dense publication. Comprehensive data on changes to coding practices across the breath of conditions included are not readily accessible – however - we would expect that such data would be highly collinear with calendar year, and therefore we do believe inclusion of calendar year in our analyses account for this in some way. We have further acknowledged this concern in the discussion as follows (linked with response to the next comment):**

***“We were unable to distinguish between subtypes of MI in our analyses. Specific criteria for the coding of ST-elevated MI (STEMI) and non-ST elevated MI (NSTEMI) were implemented towards the end of our study period following an update of the National Clinical Coding Standards - as such this work is possible in future[15]. We acknowledge that case ascertainment and changes in coding practices may vary for each health outcome - which could impact on our findings. Whilst we were unable to account for changes to coding practice directly - we did adjust for calendar year to capture some of this confounding.”***

4. Methods: I would be interested in knowing if the incidence of outcomes differs for patients who were hospitalized for STEMI vs NSTEMI.

**Whilst this was also of interest to us, unfortunately the coding guidelines in place during the study period do not enable the reliable ascertainment of the STEMI and NSTEMI subtypes of MI. Specific criteria for the coding of STEMI and NSTEMI were introduced to HES in 2015 following the implementation of the fourth edition of The National Clinical Coding Standards. Given that such updates to coding practices take time to materialise and show in routine EHR data of this kind – we feel there would be too much uncertainty in these data even if conducted for the last year of our study period. Moreover – we would be unable to present long term outcomes for NSTEMI/STEMI if conducting this only in the last year of our study and therefore the added value in this case is limited. We have acknowledged this in the discussion as per our previous comment, and highlight it as an important consideration for future work.**

5. Methods: did you limit AMI identification to patients with AMI listed in the primary diagnosis code field? I’m thinking yes, but don’t see that explicitly mentioned (lines 144-155).

**We have clarified the methods to indicate that we did not limit MI to the primary diagnostic position only. This is because MI ascertainment through the first diagnostic position only could be impacted by the presence of another dominant disease at the time of admission (e.g. cancer). We have shown this masking by more dominant conditions specifically as part of “The Virtual Cardio-Oncology Research Initiative (VICORI)” collaborative (involving co-authors Hall, Lambert and Gale) – which highlights that 77% of index MI cases are ascertained through assessing first diagnoses position only, which is increased to 97.8% if including secondary diagnoses fields in work comparing the UK’s national clinical registry of MI (Myocardial Ischaemia National Audit Project) directly with HES.[16]**

**The methods now include the following additional statement:**

**“Case ascertainment of index MI included first diagnostic position as well as secondary diagnoses, as planned *a priori*, given that ascertainment in the first position only may be impacted by presence of another dominant disease[47].”**

6. Methods: How did you handle patients without any “look back” data, such as a patient who newly immigrated to the UK and thus you would not have information on a previous MI? In such cases, how would you determine the “1st occurrence” (lines 150-153)?

**The look back period for the entire dataset covers more than 10 years of hospitalisation records - but we appreciate that for some individuals (e.g. as suggested due to immigration) these data will have been missing. A limitation which has arisen through the strict data governance and GDPR legislation is that we are only allowed access to the direct data under study. Any exclusion criteria were therefore applied at source by NHS Digital throughout the entire dataset, and they do not provide denominator data outlining the number of exclusions applied, nor a summary of the amount of lookback data available per individual so we cannot investigate the impact of this on the work undertaken. Nonetheless, we mitigate against this unknown as best as possible by fully disclosing a thorough sensitivity analyses excluding events at the same time as or in the immediate two month period post MI in the event that some of these ‘first occurrences’ may indeed be a first recording of a pre-existing condition. We further acknowledge that there may be individuals in the data with limited or no look-back data in the discussion as a study limitation as follows:**

***“For some individuals the look-back period may have been limited (e.g. due to immigration) and we were unable to quantify the impact of this.”***

7. Methods, Lines 158-166. Given the longitudinal nature of the data, could a patient exist in both the MI and non-MI cohorts? My sense is yes (for example if a patient had not had an AMI in 2010, but subsequently was hospitalized with AMI in 2015.

**Yes this is correct, and we have further clarified and highlighted this in the text as follows (see also response to Review #2, Comment 7):**

**“Due to the longitudinal nature of our data, and as per risk-set matching guidance, individuals who later went on to develop MI were censored at the date of MI for the control cohort, but contribute to both the MI and matched control cohorts in further analyses[12].”**

8. Methods, Patient and Public Involvement: This section is quite outstanding. Really terrific and thoughtful.

**Many thanks for highlighting this, as we found it a very valuable experience.**

9. Results, line 249: Can you please clarify if there were 18,343,361 codes for MI….or if there were 18,343,361 diagnosis codes for all conditions amongst patients hospitalized with MI? My sense is the later and that this represents all codes.

**Indeed it is the latter. We have clarified this further in text as follows: “There were 18,343,361 diagnoses codes covering all conditions for individuals with MI throughout the study period, of which 24.7% [n=4,526,829] were unique non-fatal diagnoses codes per individual (Table 1)”**

10. Discussion, lines 327-330: is it possible that the lower incidence of cancer reflects reduced screening of patients who have had AMI?

**We thank the reviewer for this suggestion. We have substantially expanded our discussion of the lower incidence of cancer compared with previous published work (see reviewer #2, comment 2) and additionally incorporated a comment with regards to cancer screening ; as well as stating that there may be a surveillance bias given that contact with the healthcare system increases opportunistic diagnoses of cancer.**

***“There is some evidence of reduced rates of screening for cancer amongst individuals with cardiovascular disease which could explain the findings[11]. However, there may also be a surveillance bias acting in the opposite direction.”***

11. Discussion: consider adding discussion about how study results might be incorporated into SMDM.

**Many thanks for this suggestion - we have expanded on this discussion as per our response to comment 2 above.**

12. Tables/Figures are excellent.

**Many thanks**

Peter Cram, MD MBA

Professor of Medicine

University of Texas, Galveston

Reviewer #4

Review of the paper entitled "Health outcomes after MI: a population study of 56 million people in England" by Hall et al. This paper reports on a nationwide cohort study including all individuals 18 or older admitted to NHS facilities in England between 2008 and 2017. The analysis pertains to 430 361 MI. Reliance on the electronic medical record system in place through the NHS enable the authors to report on death and on 11 non-fatal outcomes after MI including both cardiovascular and non-cardiovascular events (including heart failure, renal failure, atrial fibrillation, bleeding, diabetes, cancer, cerebro-vascular disease depression, dementia, recurrent MI, peripheral arterial disease). MIs and outcomes were ascertained by ICD 10 codes. The authors compared these outcomes with those observed in a matched population and by doing so are able to document an excess risk of mortality and morbidity except for cancer and dementia.

1. The paper includes a number of important strengths:

*	size of the data set

*	ability, through the comprehensive electronic medical record system, to report on holistic outcomes beyond the cardiovascular system.

*	comparison to matched controls

*	Information on deprivation

**Thanks for highlighting the strengths of our work.**

Several shortcomings hinder the enthusiasm for the manuscript. These include in particular:

2. The need to clarify the objectives of the paper in order to bring a sharper focus to the incremental knowledge that it will bring to existing literature. There is abundant data on cardiovascular events after MI included but not limited to the work published by the groups of Krumholz (cited only twice) Roger and Goldberg (not cited) . The relatively recent state-of-the-art review by Bahit et al (2018 J Am Coll Cardiol HF. 2018 Mar, 6 (3) 179-186 provides the useful summary of the burden of heart failure after MI. Other references that are useful to consider include among many the following:

o	Temporal Trends in Post Myocardial Infarction Heart Failure and Outcomes Among Older Adults.Kochar A, Doll JA, Liang L, Curran J, Peterson ED.J Card Fail. 2022 Apr;28(4):531-539. doi: 10.1016/j.cardfail.2021.09.001. Epub 2021 Oct 5.PMID: 34624511

o	Long-Term Mortality of Older Patients With Acute Myocardial Infarction Treated in US Clinical Practice. Kochar A, Chen AY, Sharma PP, Pagidipati NJ, Fonarow GC, Cowper PA, Roe MT, Peterson ED, Wang TY.J Am Heart Assoc. 2018 Jun 30;7(13):e007230. doi: 10.1161/JAHA.117.007230.PMID: 2996099

*	A more comprehensive review of the existing literature will help the authors identify the unique contributions of their work and will help them leverage the strengths mentioned above. In particular and Contrasting with the statement on Page 3, some of these studies present cumulative incidence data, account for confounding, and for competing risk of death.

**We thank the reviewer for their detailed comment with regards to the literature review – we have expanded our introduction with a much more specific summary of the available literature, added additional references, brought more references from our supplement into the main manuscript and articulate the unique contributions and the strengths of the work more clearly in the introduction.**

**We conducted a comprehensive literature search presented in the supplement (S1 Text, S1 Table), which has been further updated and now includes 62 papers assessing the incidence of post MI new onset, non-fatal, disease. The specific focus of our review has been more clearly articulated (S1 Text) – and we explicitly state that papers which only focus on the *determinants or predictors* of post MI disease – without providing specific estimates of incidence – were excluded. Many papers adjust for confounding and competing risk of death in additional analyses such as assessing the determinants or predictors of post MI disease – but report unadjusted measures of the actual incidence (absolute risk) of post MI disease which we uniquely address in our manuscript. Further – we found no studies which adjusted for confounding as well as censoring as well as competing risk in their measures of incidence specifically (they did however do so in models assessing the determinants of outcomes). We have now further clarified this in the manuscript introduction.**

**In addition, we identified many papers which solely focus on post MI mortality (such as Kocher *et al* 2018) – which were not included in our review as they did not report incidence of non-fatal health outcomes following MI. We instead include the related paper by Roe *et al,* 2013[17] from the same CRUSADE registry data, which reports unadjusted estimates of the composite incidence of death, readmission for MI and readmission for stroke post MI – but does account for competing risk of death.**

**We agree with the reviewer that there is an abundance of post MI heart failure research – however data of the post MI prevalence of heart failure remains highly variable[18] which has been discussed further in the introduction.**

**Upon re-review of the literature – we have included the following additional papers which met our inclusion criteria:**

1. **Kochar *et al* 2022; “Temporal trends in post myocardial infarction heart failure and outcomes among older adults”[19] (identified in previous searches as abstract only – full paper now available)**
2. **Gouda et al 2021; “Long-term risk of death and recurrent cardiovascular events following acute coronary syndromes”[20]**

**Additionally we have included the state of the art review of post MI heart failure[18] to support our introduction.**

**Finally - we re-reviewed the PubMed entries for Professor Harlan Krumholz, Professor Lee Goldberg and Dr Veronique Lee Roger. Whilst we identified additional papers relevant to the post MI setting (e.g. “Cardiac rehabilitation after MI in the community”[21] from the Roger group; and “Trends in 10-year outcomes among Medicare beneficiaries who survived after MI”[22] from the Krumholz group, and previously identified[13, 23-27] from Krumholz, Roger and Goldberg groups – these studies did not meet our inclusion criteria; although some support our introduction and discussion. Our review does already contain several relevant papers from the Roger, Krumholz and Goldberg groups including[3, 28-36] – and more of our supplemental references have been brought into the main manuscript to make this clear.**

**Our updated summary of the literature has been integrated into the introduction and provided in full in S1 Text and S1 Table.**

**Updated introduction section:**

**“*The majority of studies reporting the incidence of new onset disease following MI focussed on the short-term incidence of recurrent MI, bleeding or stroke[12-26] (full literature review available in S1 Text and S1 Table). The incidence of heart failure following MI has been studied both in the short[27, 28] and long term[29-32] – but estimates vary widely (14% to 36%)[33]. Whilst studies adjust for confounding and competing risk of death when assessing the determinants of heart failure – the absolute risk of heart failure for individuals with MI in large scale studies were reported without adjustment[3, 34-36] and therefore lack generalisability as well as being prone to bias. Evidence of the long-term incidence of atrial fibrillation[37-39] and depression[40-42] were also limited by confounding of sociodemographic factors, pre-existing disease and differential exposure times. Further, post MI incidence of depression has only been studied in fewer than 300 patients. Whilst data on the post MI incidence of cancer (9% within 17 years; n=2.1 million)[43] and dementia (9% within 35 years, n=314,911)[2] were more robust – patient demographic specific absolute risks remain unknown. There were no contemporary and nationally representative studies of the risk of new onset peripheral arterial disease, chronic renal failure or diabetes for survivors of MI (with the exception of one study assessing incidence of newly diagnosed diabetes at the time of MI in 3,500 young adults)[44].***

***In effect, published articles to date have not systematically described the incidence of non-fatal post MI disease outcomes, and none of the studies reported post MI incidence accounting for confounding as well as censoring and competing risks of death to quantify the absolute risk of outcomes over continuous follow up time.”***

**S1 Text updated as follows:**

***“We searched the Ovid Medline database (1946 to October 2023) and Embase (1996 to October 2023) using medical Subject Heading (MESH) terms “myocardial infarction” or “acute coronary syndromes” combined with outcome terms for “hospitalisation”, “events”, “multimorbidity”, “comorbidity” or “chronic conditions” with adjacency searching for “post”, “after” or “following” and “incidence”, “prevalence” or “rate”. No date or language restrictions were applied. In addition, we searched the grey literature using citations in peer-reviewed literature as well as through google scholar.*** ***Our review focused specifically on studies reporting incidence of new onset disease following myocardial infarction (MI). Studies which focused on fatal outcomes only, studies which investigated the determinants of new onset disease following MI without providing data of the incidence of outcomes directly, and studies which were only published in abstract form, were excluded.***

***Our search identified 62 studies for which we extracted information of the study design, the outcomes investigated, the reported incidence of post MI health outcomes and the methodology used for the calculation of incidence (S1 Table)….”***

3.	The ascertainment of both MI and the 11 non-fatal outcomes rely on ICD 10 codes. The limitations of reliance on codes particularly over an extended period of time should be acknowledged as coding practices may have evolved over time.

**We acknowledge that coding practices may have evolved over time – and as per our response to Reviewer #3 comment 3 – we have now added this to the study limitations of the discussion.**

**Inpatient HES data coding provides a reliable source of case ascertainment due to the Payment by Results system which has been in place for several years before our study start date (2004 onwards). Payment by Results is a pay-for performance system of secondary care reimbursement in the NHS internal market, and as such incentivises accurate diagnosis coding in HES APC data.[4] We refer to the prior validation studies of HES data, including those relating to cardiovascular disease[37] and more specifically for MI in work from the VICORI initiative in which our co-authors (Hall, Gale and Lambert) are involved[16].**

**Case ascertainment of index MI in primary diagnoses fields of HES is estimated to be high (77%) – this increases further to 97.8% when extending to secondary diagnoses fields as we have done in our analyses. Further, HES data have been shown to be more likely to capture individuals with MI with multiple long-term conditions when compared to the UK’s national clinical register of MI (Myocardial Ischaemia National Audit Project [MINAP]). This is because MINAP is an audit predominantly recorded by cardiology services, patients whose MI care is primarily supported by another specialty (e.g. cancer or palliative care) may not be referred to cardiology services and as a result are sometimes not be captured by MINAP[16].**

**In addition, in our response to reviewer #1 comment 2 we highlight the ascertainment of other conditions in HES data coding – and have added references in support of this to our discussion ([1]).**

4.	Treatment patterns are not discussed. Have they changed during that time? Were they adjusted for in the analysis?

**We acknowledge that in addition to temporal changes in definitions of MI (discussed above) there were changes to clinical practice as a result of the implementation of new medications and health technologies – which we have studied previously. In our previous work[38] we highlight that the temporal changes in invasive coronary strategy had the biggest impact on outcomes over time. In addition to accounting for changes over time by inclusion of calendar year in all our analyses - we now also adjusted for the use of invasive coronary angiography, PCI and CABG in our models for the MI cohort to alleviate these concerns.**

5.	While clearly the burden of cardiovascular and non-cardiovascular events after MI is striking particularly when presented on a national scale, the authors state on page 15 that "new strategies encompassing enhanced surveillance and detection, are required to address the latent burden of new cases of diseases associated with MI". Such vague and sweeping statements are not particularly helpful. How do the authors envision to implement and deploy the strategies that they are advocating for? The same consideration applied to the statement on page 16 lines 366 to 368.

**As per our response to the editor in chief, comment 35, page 24/25, we have updated our discussion substantially and have added a specific section on the study implications in the discussion which is more focussed on the implications associated with particular study results both in terms of specific demographic groups as well as specific health outcomes (contained in the revised manuscript discussion paragraph 8). Further – we removed the statement regarding the need for systematic interrogation of national EHR data (as per original manuscript page 16, Lines 366-368).**

6.	The similar risk of dementia among cases as compared to controls is somewhat surprising given the association between cardiovascular diseases and dementia and the excess risk of dementia among people with cardiovascular disease. This raises a concern about ascertainment issues and deserves a comment.

**We have now commented on this further in the discussion – and highlight that the finding of no difference overall is consistent with the only other population study of post MI incidence of depression – here we add to the body of evidence by providing adjusted estimates of post MI incidence, as well as provide estimates over continuous follow up time which vary between MI and matched controls. Additionally, we would like to highlight that whilst the ascertainment of dementia in HES is high (~85%) when compared with primary care data, there are some known delays in dementia recording in HES data estimated at a delay of approximately 1.6 years.**

**Our discussion has been updated with these discussion points as follows:**

***“The incidence of dementia overall and vascular dementia was 7.8% and 2.3% respectively within 9 years following MI. There was no difference in the risk of dementia overall, but the risk of vascular dementia following MI was significantly higher (13% increased risk), than matched controls consistent one previous study[2]. Our study improves on these previous data by providing adjusted estimates of cumulative incidence over continuous follow up time alongside detailed age, sex, deprivation specific risk. Our inclusion of time-dependent effects showed an increased risk of dementia overall for MI compared with matched controls in the short-term (2.1% vs. 1.8% at 60 days) but reduced risk in the long term (7.8% vs. 8.3% at 9 years); and a consistently increased risk in vascular dementia at all time points.“***

**and in the limitations section:**

***“ …. whilst there is evidence of delayed dementia diagnoses in HES compared with primary care of 1.6 years, ascertainment of dementia overall is also high (85%)[2].”***

7.	Reliance on hospitalized events for outcome ascertainment introduce a significant degree of bias which may apply differentially depending on the outcomes under consideration. Reliance of hospitalization is particularly problematic for cancers and dementia events that were not significantly increased after MI in the data set.

**As per earlier response about case ascertainment in HES – we agree with the reviewer that this could vary according to outcome as highlighted. Whilst this may lead to under-counting of events in HES overall – it would not bias our comparisons of the post MI and post non-MI hospitalised cohorts as these are both derived from HES and therefore would both be prone to under-counting of the same degree. We acknowledge the overall potential underestimation of outcomes in our discussion, but add that it could vary by outcome:**

***“Our primary outcomes were hospitalised events and not diagnoses made in primary care settings. Therefore, the totality of post MI disease may be underestimated - which could vary according to outcome.*** ***Nonetheless, conditions captured in this study can be framed as clinically, epidemiologically and economically important given that admission to hospital is a key clinical event, often signifying later-stage, more severe or complex disease. We did not adjust for severity of hospitalisation in our analyses – but our comparator cohort captures the full range of admissions in that it includes all individuals without MI and was not limited to less severe disease.”***

**Having said that – diagnostic and procedure coding in HES is incentivised by the NHS’s payment by results system, and as such case ascertainment in general is high. In addition - we also want to highlight that in some cases a cancer diagnoses code may mask the prevalence of other conditions in the primary diagnoses field (see discussion in response to reviewer #3, comment 5) and to mitigate against *over* counting of cancer diagnoses we limit our analyses to the first occurrence identified in HES. We have compared cancer incidence in the post MI hospitalised population with the non-MI hospitalised population – undercounting of cancer cases would apply to both the MI and matched control cohorts equally and as such this would not explain our findings. We are therefore confident that our results, relating to the lower incidence of post MI cancer in this study, which is consistent with several of the findings from the nationwide Danish study of post MI cancer[5] as well as a recent study published in JACC[6] are unlikely a result of lower case ascertainment of cancers recorded in HES APC. We have now provided a much more detailed discussion of the potential reasons for lower incidence of cancer observed in our study (see response to reviewer #2, comment 2).**

**Whilst there are no full population studies assessing the case ascertainment of dementia in HES (owing to a lack of population based clinical registries in dementia) – there is evidence of high case ascertainment for dementia derived from a female-only study population (85% for dementia overall and up to 88% for vascular dementia specifically).[2] In addition, the study noted a delay of an average of 1.6 years of dementia coding in HES compared with primary care – and we have added the following to the discussion:**

***“….and whilst there is evidence of delayed dementia diagnoses in HES compared with primary care of 1.6 years, ascertainment of dementia overall is high (85%)[2].”***

8	The discussion is difficult to follow and would benefit from adding sub-headings and focusing for example on cardiovascular outcomes first followed by non-cardiovascular events etc. Some of the statements about secondary prevention and the risk of bleeding are not supported by the data. Are there any data on the use of dual antiplatelet therapy in that cohort? Did the risk of bleeding decrease overtime as dual antiplatelet regimens were altered?.

**As per our response to the editor in chief (comment 35, page 24-25) we have restructured and expanded our discussion – although the editorial team have requested we do not include sub-headers. We now follow the following requested structure:**

- **A short summary of the articles key findings (discussion paragraph 1),**
- **A detailed summary of the findings in the context of existing research, including differences in results with previous studies and what our study adds to existing research (paragraph 2 covering heart failure, bleeding, diabetes, peripheral vascular disease and renal failure; paragraph 3 covering depression, paragraph 4 covering cancer and paragraph 5 covering dementia)**
- **Substantially expanded strengths and limitations of the study (paragraphs 6 and 7)**
- **Implications and next steps for research, clinical practice, and/or public policy – we have added a new section to summarise the clinical implications of our research in one place (paragraph 8, 9, 10).**
- **One paragraph conclusion (paragraph 11).**

**In addition, in response to this comment and that from other reviewers – we have been more explicit in that we do not have data on secondary preventative medication throughout the discussion. Instead we place our findings in the context of existing work combined with the new data presented by our manuscript, to outline potential future implications.**

**GENERAL COMMENTS**

1. Please cite the reference numbers in square brackets (e.g., “We used the techniques developed by our colleagues [19] to analyze the data”). Citations should be preceding punctuation.

**We have updated this throughout the manuscript and supporting information.**

2. Please cite your Supporting Information as outlined here: <https://journals.plos.org/plosmedicine/s/supporting-information>

**The supporting information has been re-labelled and re-cited throughout the manuscript as per the guidelines.**

3. We have noticed that you have used many different footnote markers in your tables and figures. We suggest that you use a consistent format and not mix footnote markers in the same figure/table.

**We now consistently use alphabetised superscripts for all table and figure footnotes.**

4. The Data sharing statement (ll.430-454) should be included in the Data Availability section of the manuscript submission form.

**This has been included as requested.**

5. The Competing Interests section (ll.401-411) and the Role of the funding source (ll.412-421) should be included in the corresponding sections of the manuscript submission form.

**This has been included as requested.**

6. Please remove the ‘Transparency statement’ from the manuscript.

**This has now been removed from the revised manuscript as requested.**

7. For all observational studies, in the manuscript text, please indicate: (1) the specific hypotheses you intended to test, (2) the analytical methods by which you planned to test them, (3) the analyses you actually performed, and (4) when reported analyses differ from those that were planned, transparent explanations for differences that affect the reliability of the study's results. If a reported analysis was performed based on an interesting but unanticipated pattern in the data, please be clear that the analysis was data-driven.

- Did your study have a prospective protocol or analysis plan? Please state this (either way) early in the Methods section.
- If a prospective analysis plan (from your funding proposal, IRB or other ethics committee submission, study protocol, or other planning document written before analyzing the data) was used in designing the study, please include the relevant prospectively written document with your revised manuscript as a Supporting Information file to be published alongside your study, and cite it in the Methods section. A legend for this file should be included at the end of your manuscript.
- If no such document exists, please make sure that the Methods section transparently describes when analyses were planned, and when/why any data-driven changes to analyses took place.
- In either case, changes in the analysis-- including those made in response to peer review comments-- should be identified as such in the Methods section of the paper, with rationale.

**Whilst no formal statistical protocol relating solely to this paper is available for publishing – the methods are clearly labelled as *a priori* where appropriate throughout. We have now added a clear study hypothesis to the introduction and methods. Instances where analyses differed from original plans, were data driven or were amended as a result of peer-review have also been clearly highlighted throughout the methods section.**

**Updated introduction hypothesis:**

***“For each of these health outcomes we hypothesised that the incidence following MI differed than that expected during a life course without MI. Therefore, we determined the excess incidence, estimated the adjusted absolute risk, and presented age, sex, deprivation and time specific risk charts for subsequent MI, heart failure, peripheral arterial disease, cerebrovascular disease, chronic renal failure, severe bleeding, diabetes, atrial fibrillation, cancer, depression, dementia and all-cause mortality following MI compared with matched controls.”***

**The following additional paragraph has been added to the methods:**

***“Our a priori study hypothesis was that the risk of major health outcomes following MI differed than that expected during a life course without MI. The study methodology was defined a priori throughout – exceptions to this, including any data driven decisions and analyses conducted in response to peer-review, are labelled as such throughout the methods section.”***

**EDITOR-IN-CHIEF COMMENTS**

1. The manuscript in its current form does not seem to be well placed in the current literature, and it is also crucial to discuss what the study adds to existing research and how the results compare to previous research.

**We conducted a thorough in-depth review of the literature in our preparation of this manuscript which has been updated and includes 62 peer-reviewed publications assessing the burden of new onset disease following MI. We supply relevant details of our search strategy in S1 Text and details of the included papers in S1 Table. We provide a detailed response regarding the specific additional papers highlighted by reviewer 4 (Reviewer #4, comment 2). In addition, our revised introduction includes more detail about what this study adds to the body of existing literature. Additionally, we cite an existing review of the heart failure literature as well as moved many of the references from the supplement into the main body of the manuscript to further justify the need for our work.**

**Updated literature review in the introduction:**

***“The majority of studies reporting the incidence of new onset disease following MI focussed on the short-term incidence of recurrent MI, bleeding or stroke[12-26] (full literature review available in S1 Text and S1 Table). The incidence of heart failure following MI has been studied both in the short[27, 28] and long term[29-32] – but estimates vary widely (14% to 36%)[33]. Whilst studies adjust for confounding and competing risk of death when assessing the determinants of heart failure – the absolute risk of heart failure for individuals with MI in large scale studies were reported without adjustment[3, 34-36] and therefore lack generalisability as well as being prone to bias. Evidence of the long-term incidence of atrial fibrillation[37-39] and depression[40-42] were also limited by confounding of sociodemographic factors, pre-existing disease and differential exposure times. Further, post MI incidence of depression has only been studied in fewer than 300 patients. Whilst data on the post MI incidence of cancer (9% within 17 years; n=2.1 million)[43] and dementia (9% within 35 years, n=314,911)[2] were more robust – patient demographic specific absolute risks remain unknown. There were no contemporary and nationally representative studies of the risk of new onset peripheral arterial disease, chronic renal failure or diabetes for survivors of MI (with the exception of one study assessing incidence of newly diagnosed diabetes at the time of MI in 3,500 young adults)[44].***

***In effect, published articles to date have not systematically described the incidence of non-fatal post MI disease outcomes, and none of the studies reported post MI incidence accounting for confounding as well as censoring and competing risks of death to quantify the absolute risk of outcomes over continuous follow up time.”***

**We have updated the discussion of the manuscript substantially in response to both editorial and reviewer comments – including highlighting what the study adds over and above existing research, and by providing more detailed comparisons to existing research (see detailed response in “Editor in Chief Comments, Comment 35” below))**

2. In addition, propensity score matching should be considered for better matching of individuals.

**Propensity scoring is commonly used in studies comparing treatment outcomes, with the aim of balancing covariate patterns between treated and untreated individuals (indeed we have experience of using IPW propensity scoring in our previous work for this purpose[39]). Here, our primary focus was to determine the incidence of a number of non-fatal health outcomes over time in a cohort of individuals with MI and those without MI, and we account for imbalances between cohorts at several stages of our analyses including 1) via risk-set matching on key variables including single year of age, sex, year of hospitilisation and hospital trust, 2) further adjustment in modelling for age, sex, deprivation, year of study entry and, following peer review, invasive management of MI and 3) standardisation by age, sex, deprivation and follow up time in our demographic specific risk charts.**

**The risk-set matching procedure we adopted facilitated robust comparison of incidence between individuals with MI and those without, or prior to developing, MI. This approach minimises the risk of bias in cohort demographics whilst concurrently minimising the risk of immortal time bias by allowing us to carefully control the study follow up time and censoring times for the matched control cohort. Moreover – given the large database we were able to conduct an exact matching procedure. Our study therefore mimics a fully blocked experimental design, without the need for pruning based on arbitrary callipers which can result in a loss of efficiency in analyses (as is a key disadvantage of PSM). More recently, concerns have been raised over the ability of pruning necessary for PSM to *introduce* further bias which we wished to avoid[40]. We have included the following additional justification in our introduction:**

**“Our *a priori* analyses plans included a comparison of outcomes between those with MI compared with all individuals without MI. However, to minimise bias arising from different demographic profiles between groups as well as to avoid immortal time bias – we instead employed a risk-set matching procedure. This approach was chosen by the authors prior to peer review, in favour of propensity score matching, to avoid the need for pruning of data resulting in a loss of efficiency as well as the potential risk of re-introducing bias[47].”**

ABSTRACT

3. Abstract Methods and Findings:

* Please provide brief demographic details of the study population (e.g. sex, age).

* Please include the main outcome measures.

* In the last sentence of the Abstract Methods and Findings section, please describe the main limitation(s) of the study's methodology.

**The above have been updated , the main limitation of the study has been listed as follows in the abstract: “The study includes data from secondary care only – as such diagnoses made outside of secondary care may have been missed leading to potential under estimation of the total burden of disease following MI.”**

4. Abstract Conclusions:

* Please address the study implications without overreaching what can be concluded from the data; the phrase "In this study, we observed ..." may be useful.

* Please interpret the study based on the results presented in the abstract, emphasizing what is new without overstating your conclusions.

* Please avoid vague statements such as "these results have major implications for policy/clinical care" and please avoid assertions of primacy ("We report for the first time...."). Mention only specific implications substantiated by the results.

**The updated abstract concludes as follows:**

***“In this study up to a third of patients with MI developed heart failure or renal failure, 7% had another MI and 38% died within 9 years. The incidence of all health outcomes, except dementia or cancer, was higher than expected during the normal life course without MI following adjustment for age, sex, year and socioeconomic deprivation.*** ***Efforts targeted to prevent or limit the accrual of chronic, multi-system disease states following MI are needed and should be guided by the demographic specific risk charts derived in this study.”***

5. Please ensure that all numbers presented in the abstract are present and identical to numbers presented in the main manuscript text.

**This has been checked for consistency.**

6. PLOS Medicine requests that main results are quantified with 95% CIs as well as p values. When reporting p values please report as p<0.001 and where higher as the exact p value p=0.002, for example. For the purposes of transparent data reporting, if not including the aforementioned please clearly state the reasons why not.

**We now include p-values for the hazard ratio estimates obtained from our flexible parametric survival models throughout the abstract, manuscript text and figures in the specified format. When we report values of the cumulative incidence of new-onset disease, we are not testing a pre-specified null hypothesis. Whilst no p-values are provided alongside these, we do report all our cumulative incidence estimates with 95% confidence intervals as a measure of uncertainty.** **We have opted to report Table 1 without p-values, given that the large size of our study renders all p-values uninformative (at p<0.001), and instead we wish the readers’ attention to be focussed on the actual differences observed. This also aligns with STROBE Explanation and Elaboration Publication which discourages the use of significant testing/p-values for descriptive table (**[**https://doi.org/10.1371/journal.pmed.0040297**](https://doi.org/10.1371/journal.pmed.0040297)**).**

7. Please include any important dependent variables that are adjusted for in the analyses.

**We now state “…adjustment for age, sex, year and socioeconomic deprivation” in the abstract.**

8. Throughout, suggest reporting statistical information as follows to improve clarity for the reader “22% (95% CI [13%,28%]; p</=)”. Please amend throughout the abstract and main manuscript. Please note the use of commas to separate upper and lower bounds, as opposed to hyphens as these can be confused with reporting of negative values.

**We have updated this formatting throughout the abstract, manuscript and supplement.**

9. When a p value is given, please specify the statistical test used to determine it.

**We provide p-values alongside HRs (as per response to point 6 above), for which we indicate a flexible parametric survival model has been used in abstract as well as main text.**

10. l.48: Please define ‘SD’ at first use and ensure to define all statistical abbreviations at first use in the abstract.

**This has been updated for SD, CI and HR.**

11. AUTHOR SUMMARY

Thank you for providing a ‘Research in Context’ section. At this stage, we ask you to exchange this section with a short, non-technical Author Summary of your research to make findings accessible to a wide audience that includes both scientists and non-scientists. The Author Summary should immediately follow the Abstract in your revised manuscript. This text is subject to editorial change and should be distinct from the scientific abstract. Please see our author guidelines for more information: <https://journals.plos.org/plosmedicine/s/revising-your-manuscript#loc-author-summary>.

The summary should include 2-3 single sentence, individual bullet points under each of the questions. The last bullet point should describe the main limitation(s) of the study's methodology.

It may be helpful to review currently published articles for examples which can be found on our website here <https://journals.plos.org/plosmedicine/>.

**We have removed the research in context section, and instead include an author summary in the manuscript – referring to the guidelines and examples.**

INTRODUCTION

12. Please sufficiently address past research and explain the need for and potential importance of your study. Indicate whether your study is novel and how you determined that. If there has been a systematic review of the evidence related to your study (or you have conducted one), please refer to and reference that review and indicate whether it supports the need for your study.

**As per the opening comment from the Editor-in-Chief, as well as in response to Reviewer #4, comment 2 we have updated our review of the literature in the introduction providing additional detail. Full details of our search strategy and papers extracted are provided in S1 Text and S1 Table. In addition – we refer to a published systematic review of the post MI burden of heart failure in the manuscript[18], and further highlight clear gaps in evidence with reference to previous work throughout the newly written introduction (see response to Reviewer #4, comment 2).**

13. Please conclude the Introduction with a clear description of the study question or hypothesis.

**The introduction now concludes with a hypothesis as per earlier response (General Comments, #7).**

14. l.100: Please define ‘US’ at first use. The reference is better placed at the end of the statement.

**This has been updated.**

15. ll.120-123: Please use the past tense to describe what you did in your study.

**The introduction has been updated to past tense throughout.**

METHODS AND RESULTS

16. Please ensure that the study is reported according to the RECORD guideline and include the completed RECORD checklist as Supporting Information. You may also include the CODE-EHR checklist. When completing the checklist, please use section and paragraph numbers, rather than page numbers. Please add the following statement, or similar (adjust ll.204-205), to the Methods: "This study is reported as per the REporting of studies Conducted using Observational Routinely-collected Data (RECORD) guideline (S1 Checklist)."

**Our study was reported against the RECORD guidelines and CODE-EHR checklist (not least because our co-authors developed the latter), and the checklists provided in S1 Checklist and S2 Checklist. We have now updated these with the relevant section and paragraph numbers relating to the revised manuscript. Further, the sentence in the methods referring to the checklists have been updated as advised.**

17. PLOS Medicine requests that main results are quantified with 95% CIs as well as p values. When reporting p values please report as p<0.001 and where higher as the exact p value p=0.002, for example. For the purposes of transparent data reporting, if not including the aforementioned please clearly state the reasons why not.

**As per our response to point 6 above, we now include p-values for the hazard ratios estimated from our flexible parametric survival modelling throughout the manuscript text and figures in the specified format.**

18. Please include any important dependent variables that are adjusted for in the analyses.

**An additional sentence has been added to the cumulative incidence methods section to clarify that the adjustments are based on the same set of models as the ones specified in the previous section (namely, adjusting for non-linear age using restricted cubic splines, sex, calendar year, deprivation score and a time-dependent effect for cohort). Additionally, we have added the following to the methods:**

**“Following peer-review, age, sex and deprivation specific risk for the MI cohort were additionally adjusted for receipt of invasive coronary angiography, PCI or CABG at time of MI.”**

19. Suggest reporting statistical information as detailed above – see under ABSTRACT

**We have updated the format of confidence intervals and have provided *p*-values as mentioned above.**

20. Please present numerators and denominators for percentages, at least in the Tables [not necessarily each time they're mentioned].

**Table 1 has been updated to include denominators in each cell.**

21. l.126: Is it ‘all individuals aged >18 years’ or ‘all individuals aged ≥18 years’?

**This has been corrected throughout the manuscript to ≥18 years, including in Figure 1.**

22. l.145: Please define ‘HDR’ and ‘UK’.

**Both are now defined.**

23. l.177: Please use a consistent format when writing ‘post MI’/’post-MI’. Please revise throughout the entire manuscript.

**This has been updated throughout the manuscript and supplement to ‘post MI’**

24. ll.181-182, please change to: “Each model was adjusted for age, sex, calendar year, deprivation score and a time-varying covariate for cohort.”

**This has been updated – with terminology also updated to “time-dependent effect” as per response to Reviewer #2, comment 3.**

25. l.191: Please revise the definition of age groups, e.g. 40 years old should not be included in two age groups (i.e. ≤40 and 40 to ≤50).

**Age group description has been updated to the following mutually exclusive groups: <40, 40 to <50, 50 to <60, 60 to <70, 70 to <80, 80 to <90 and ≥90 years of age in the manuscript text as well as in Figure 4 and online supplement.**

26. l.220: Please define ‘PPIE’ at first use.
27. l.231: Please define ‘GP’ at first use.

**These have now been defined at first use.**

28. l.246: We suggest adding the numbers for ‘minimal differences in deprivation between cohorts’.

**The following has been added to results paragraph 1: *“There were minimal differences in socioeconomic deprivation between the cohorts (20.7% [n=77,008] vs. 19.2% [n=375,734] were in the most deprived category for MI and matched controls, respectively), …”***

29. l.253: For easy comparison, we suggest adding the numbers for the matched controls. Please report numerical results together with their statistical information whenever possible – please revise throughout the manuscript.

**The results text has been expanded as follows (results, paragraph 2):**

***“The majority of diagnosis codes related to the circulatory system (44.3% [n=2,003,429]), which along with ‘endocrine, nutritional and metabolic diseases’ (9.6% [n=433,545]) appeared more frequently than in matched controls (20.2% [n=3,631,165] and 9.0% [n=1,616,515] for circulatory and ‘endocrine, nutritional and metabolic diseases’, respectively)."***

**We have not added p-values here as per comment 6 above regarding Table 1 descriptives.**

30. l.277: Please add a right parenthesis following ‘(9.0% [8.9─9.1%] vs. 8.8 [8.8─8.9%]’.
31. l.277/278/279: Please add a unit (here, %) when necessary (e.g., for 8.8, 1.79, 8.34) - – please revise throughout the manuscript.

**These typos have now been corrected, and updated formatting with units throughout for each confidence interval component**.

32. l.284: Given the broad and global audience, we suggest adding more information about the Index of Multiple Deprivation in the Methods section, for example, explaining the meaning of the quintiles.

**The methods have been expanded as follows:**

***“******Patient demographics including age (continuous), sex (male/female), year (single year of study entry), socioeconomic deprivation (measured using the Index of Multiple Deprivation [IMD][49] – the official score of relative deprivation for small geographic areas of England (lower super output areas) categorised into 5 quintiles from least deprived [quintile 1] to most deprived [quintile 5]),….”***

**The following reference has also been added:**

***“49. Communities and Local Government (2011). "The English Indices of Deprivation 2010." https://assets.publishing.service.gov.uk/government/uploads/system/uploads/attachment_data/file/6320/1870718.pdf Date Accessed: 19/10/2023.”***

33. ll.289-291: Please revise the statement. Your study is observational and therefore causality cannot be inferred. Please remove language that implies causality, such as ‘effect’. Refer to associations instead.

**Although the matching approach we employed is analogous to a fully blocked randomised study design to allow derivation of cause and effect within observational data - we acknowledge that there may be residual confounding or additional biases in these data that we were unable to account for. Therefore, we have updated the statement to refer to associations instead.**

34. l.302: Please also include the link to the interactive version to the Data availability statement in the manuscript submission form.

**This has been included in the manuscript submission form.**

DISCUSSION

35. The discussion in its present form does not seem to be of sufficient depth. Please present and organize the Discussion as follows: a short, clear summary of the article's findings; what the study adds to existing research and where and why the results may differ from previous research; strengths and limitations of the study; implications and next steps for research, clinical practice, and/or public policy; one-paragraph conclusion. Please remove any subheadings.

**We have now re-organised our discussion, and substantially expanded it based on reviewer comments, as follows:**

- **A short summary of the articles key findings (discussion paragraph 1),**
- **A detailed summary of the findings in the context of existing research, including differences in results with previous studies and what our study adds to existing research (paragraph 2 covering heart failure, bleeding, diabetes, peripheral vascular disease and renal failure; paragraph 3 covering depression, paragraph 4 covering cancer and paragraph 5 covering dementia)**
- **Substantially expanded strengths and limitations of the study (paragraphs 6 and 7)**
- **Implications and next steps for research, clinical practice, and/or public policy – we have added a new section to summarise the clinical implications of our research in one place (paragraph 8, 9, 10).**

***“Although clearly a public health focus must be the prevention of MI, evidence from our study has implications for clinical care in Cardiology and beyond. We evidence the excess incidence of conditions which are targeted through current secondary preventative guidelines (heart failure), conditions not directly included in current post MI secondary preventative guidelines (chronic renal failure, cerebrovascular disease and peripheral vascular disease), conditions which would benefit from early detection for improved outcomes (severe bleeding and atrial fibrillation) and conditions which have a significant impact on quality of life (depression). Whilst we did not have data to assess the impact of secondary preventative medication on outcomes directly in our study, the implications of our research placed in context of previous research indicate that 1) improved secondary preventative medication for younger individuals in the most socioeconomically deprived group may tackle the high incidence of post MI heart failure observed amongst this population demographic - given the underuse of secondary preventative medication amongst socioeconomically deprived individuals with MI[41] and 2) a reduction in the long term high incidence of major bleeding complications post MI may be achieved through shortened dual antiplatelet regimes therapy[42] and longer term surveillance. The high incidence of chronic renal failure, cerebrovascular disease and peripheral arterial disease following MI suggest there may opportunity for intensified secondary prevention of shared modifiable risk factors and enhanced post MI health surveillance to mitigate against increased healthcare usage and premature death. Moreover, interventions such as screening for the most at-risk groups for post MI depression (including individuals who are younger, socio-economically deprived and females) should be considered. Whilst the incidence of vascular dementia contributes only a small proportion of the overall post MI disease burden, the marked increased risk compared with matched controls warrants further study into the causal links between MI and vascular dementia to identify opportunities for secondary prevention of the excess incidence observed.”***

- **One paragraph conclusion (paragraph 11).**

TABLES

36. Please note the use of commas to separate upper and lower bounds, as opposed to hyphens as these can be confused with reporting of negative values.

**This has been updated throughout the manuscript and supplementary tables as well as in the manuscript text.**

37. Please define abbreviations used in the tables (including those in Supporting Information files).

Table 1: Please add the unit ‘years’ for ‘Age at study entry (mean, SD)’.

Table 1: We suggest changing ‘Sex (N, % Male)’ to ‘Male Sex (N, %)’.

Table 1: For ‘Socioeconomic Status’, we suggest adding a footnote explaining that this is according to the Index of Multiple Deprivation.

Table 1: Please define ‘KM’, ‘ICD’.

Table 1: For consistency, please use the term ‘3-digit’ throughout the table and the table description.

**All of the above have been updated.**

FIGURES

For all Figures, please ensure that you have complied with our figures requirements: <https://journals.plos.org/plosmedicine/s/figures>

**All out figures have been checked/updated to comply with the requirements.**

38. Please define abbreviations used in the figure legend of each figure (including those in Supporting Information files).

**Figure legends in the main manuscript and supplement have been updated with all abbreviations listed.**

39. Please consider avoiding the use of red and green in order to make your figure more accessible to those with colour blindness (including those in Supporting Information files).

**Whilst we used red in isolation (not next to green) in our submission – we have nonetheless changed our heatmaps to a blue graded scale complying fully with colour blindness requirements, which is now additionally compatible with grey scale printing.**

40. Figure 1

Figure 1: Please define ‘MI’, ‘HES’, ‘NHS’.

Figure 1: The footnotes for ‘$$$’ and ‘**’ are missing.

Figure 1: Due to the size of the fonts, the information provided in the boxes is difficult to read and the footnotes are not visible.

Figure 1: We suggest adding details about the matched cohort in the figure description (i.e., What are the matching factors?).

**Figure 1 has been updated to reflect the above changes, and the font size has been increased throughout the figure and footnote.**

41. Figure 2

Figure 2: Please add a unit for age. Please change ‘sd’ to ‘SD’. Please define ‘MI’, ‘NA’, ‘SD’, ‘vs’.

Figure 2: Could the crude incidence be presented as a Supporting Information file? The figure contains a lot of information and could be confusing for the reader. If you consider the information on the crude incidence to be central to the understanding of the paper, we suggest splitting this figure into a table showing the crude incidence and a figure showing the excess rate.

**Figure 2 has been updated to reflect the above, including confidence interval reformatting. The crude incidence rates have been removed and moved to S3 Table and p-values have been added alongside the reported hazard ratios.**

42. Figure 3

Figure 3: Please be consistent describing numbers in words or numerals. Figure 3: The graphs are much too small. Please increase their sizes. Figure 3: Please change ‘*includes all cancer […]’ to ‘*includes all cancer types […]’. Figure 3: We suggest changing the graphs so that the y-axis is identical for all figures to facilitate comparison.

Figure 3: In the description it is suggested that the 95% CIs are presented as shaded areas, but none are visible. Is this correct? Figure 3: We suggest adding a definition of the 11 non-fatal health outcomes and 9 key subgroups to the figure description. Figure 3: Please define ‘MI’, ‘ICD’.

**The figure title has now been updated, and the numerals removed to accommodate the further changes highlighted herewith. We have created a two-part figure which allowed us to increase the size of the subplots. We have updated the plots to use consistent y-axis scales throughout – except for the plot for all-cause mortality for which the range exceeded any of the other outcomes. We have highlighted the difference in scale for this one plot by use of a footnote and place it on a separate row to avoid comparison issues. The 95% confidence intervals have indeed been plotted but are very narrow due to the size of the data and therefore they are not obvious on most figures (but can be seen when zooming in on the electronic high-resolution versions). However – we felt it was important to show via the legend that these were plotted to indicate that there was no overlap in the confidence intervals between groups. The figure descriptions have been updated with the specific list of health outcomes rather than stating how many of them there are, and finally, all abbreviations have been defined in the figure footnote.**

43. Figure 4

Figure 4: Please define ‘MI’. Figure 4: Please add a header or similar for the description of time points (60 days Male etc.). Figure 4: Where does the asterisk belong to?

**Abbreviations have been defined, additional headers added, colour scheme updated and superscripts corrected and consistently labelled using alphabetised superscripts.**

SUPPLEMENTARY MATERIAL

44. For references provided in the Supplementary Material – see under REFERENCES.

**Done**

45. Methods S1/S2/S3: Please define all abbreviations at first use including the titles (e.g., ‘MI’ or ‘US’).

**Done**

46. Table S1: Please thoroughly check for any abbreviations used in the table and define these in or underneath the table. Please write ‘SD’ instead of ‘sd’.

**Done**

47. Table S1: For Jernberg 2015, are these 95% CI values presented in ‘Post-MI disease incidence results’? Please ensure to define the statistical details in parenthesis. Please revise throughout the entire table.

**Updated throughout.**

48. Table S1: For Roe 2013, please add a unit for age (column ‘Cohort details’). Please revise throughout the entire table.

**Updated throughout.**

49. Table S1: For Grodzinsky 2015, please add a square bracket open in ‘CAD IRR = 0.89, 95% CI 0.77–1.02]’.

**Done**

50. Table S2: Please define ‘ICD’, ‘OPCS’, ‘NEC’, ‘NOC’.

**Done**

51. Table S3: Please add a unit for age. Please change ‘sd’ to ‘SD’. Please define ‘CI’, ‘MI’, ‘SD’.

**Done**

52. Table S3/S4/S5/S7: We suggest adding details about the matched cohort in the table description (i.e., What are the matching factors?).

**Done**

53. Table S4: Please define ‘CI’, ‘MI’, ‘NA’.

**Done**

54. Table S5: Please define ‘CI’, ‘MI’, ‘NA’.

**Done**

55. Table S5: Please define ‘MI’. Please use a consistent number format (2994 versus 3,032).

**Done**

56. Table S7: Please define ‘NA’.

**Done**

57. You have used different descriptions for ‘death without disease’ including ‘death without outcome’ or ‘death without event’. For clarity, please use a consistent description throughout the Supplementary Tables.

**This has been updated throughout the supplementary tables to ‘death without outcome’**

58. Figure S1: Where is footnote ‘a’? There is no figure description. The lines in the graphs are dashed but the legend depicts solid lines – please revise. Please see comments for Figure 3 and revise accordingly.

**Apologies for these inconsistencies – this has now been updated and all formatting aligns to that of the main manuscript.**

59. Figure S2/S4: Please define ‘MI’. Please add a header or similar for the description of time points as it is not clear what they refer to (60 days Male, 1 year Male etc.). We suggest adding details about the matched cohort in the figure description (i.e., What are the matching factors?).

**For all risk chart figures – we have now amended the labelling to distinguish the headers as “60 days following study entry” and “Male”. We have also amended the Figure legend to highlight the follow up times presented. We have additionally and consistently added a description of the matching variables to each relevant figure description.**

60. Figure S3: Please define ‘MI’. Please add a header or similar for the description of time points as it is not clear what they refer to (60 days Male etc.)

**As above, this has been updated.**

REFERENCES

61. PLOS uses the numbered citation (citation-sequence) method and first six authors, et al. Please ensure that journal name abbreviations match those found in the National Center for Biotechnology Information (NCBI) databases (<https://www.ncbi.nlm.nih.gov/nlmcatalog/journals>), and are appropriately formatted and capitalised. Please also see <https://journals.plos.org/plosmedicine/s/submission-guidelines#loc-references> for further details on reference formatting.

**The referencing style has been updated to follow the PLOS guidance in both the manuscript and supplementary material, and journal names have been abbreviated accordingly.**

62. Where website addresses are cited, please specify the date of access.

**Each website has been revisited to ensure it remains active, and the latest access date added.**

**REFERENCES (to support response to review)**

1.	Nimmo A, Steenkamp R, Ravanan R, Taylor D. Do routine hospital data accurately record comorbidity in advanced kidney disease populations? A record linkage cohort study. BMC Nephrol. 2021;22(1):1-10.

2.	Brown A, Kirichek O, Balkwill A, Reeves G, Beral V, Sudlow C, et al. Comparison of dementia recorded in routinely collected hospital admission data in England with dementia recorded in primary care. Emerg Themes Epidemiol. 2016;13:1-9.

3.	Chen HY, Tisminetzky M, Lapane KL, Yarzebski J, Person SD, Kiefe CI, et al. Decade‐long trends in 30‐day rehospitalization rates after acute myocardial infarction. J Am Heart Assoc. 2015;4(11):e002291.

4.	Herbert A, Wijlaars L, Zylbersztejn A, Cromwell D, Hardelid P. Data Resource Profile: Hospital Episode Statistics Admitted Patient Care (HES APC). Int J Epidemiol. 2017;46(4):1093-i. doi: 10.1093/ije/dyx015.

5.	Malmborg M, Christiansen CB, Schmiegelow MD, Torp-Pedersen C, Gislason G, Schou M. Incidence of new onset cancer in patients with a myocardial infarction–a nationwide cohort study. BMC Cardiovasc Disord. 2018;18(1):1-9.

6.	Bell CF, Lei X, Haas A, Baylis RA, Gao H, Luo L, et al. Risk of cancer after diagnosis of Cardiovascular Disease. JACC: CardioOncology. 2023.

7.	Rothwell PM, Price JF, Fowkes FGR, Zanchetti A, Roncaglioni MC, Tognoni G, et al. Short-term effects of daily aspirin on cancer incidence, mortality, and non-vascular death: analysis of the time course of risks and benefits in 51 randomised controlled trials. Lancet. 2012;379(9826):1602-12.

8.	Rothwell PM, Fowkes FGR, Belch JF, Ogawa H, Warlow CP, Meade TW. Effect of daily aspirin on long-term risk of death due to cancer: analysis of individual patient data from randomised trials. Lancet. 2011;377(9759):31-41.

9.	Mills EJ, Wu P, Alberton M, Kanters S, Lanas A, Lester R. Low-dose aspirin and cancer mortality: a meta-analysis of randomized trials. Am J Med. 2012;125(6):560-7.

10.	Davidson KW, Barry MJ, Mangione CM, Cabana M, Chelmow D, Coker TR, et al. Aspirin use to prevent cardiovascular disease: US Preventive Services Task Force recommendation statement. JAMA. 2022;327(16):1577-84.

11.	Matsumura K, Kakiuchi Y, Tabuchi T, Takase T, Maruyama M, Ueno M, et al. Cancer screening: Possibility of underscreening in older adult population with a history of cardiovascular disease. J Cardiol. 2022;80(2):133-8.

12.	Rosenbaum PR. Risk-Set Matching. Design of Observational Studies. New York, NY: Springer New York; 2010. p. 223-35.

13.	Navar AM, Wang TY, Mi X, Robinson JG, Virani SS, Roger VL, et al. Influence of Cardiovascular Risk Communication Tools and Presentation Formats on Patient Perceptions and Preferences. JAMA Cardiol. 2018;3(12):1192-9. doi: 10.1001/jamacardio.2018.3680.

14.	Ferrer RA, Klein WM. Risk perceptions and health behavior. Curr Opin Psychol. 2015;5:85-9.

15.	Nedkoff L, Lopez D, Goldacre M, Sanfilippo F, Hobbs M, Wright FL. Identification of myocardial infarction type from electronic hospital data in England and Australia: a comparative data linkage study. BMJ Open. 2017;7(11):e019217.

16.	Coles B, Teece L, Weston C, de Belder MA, Oliver-Williams C, Welch CA, et al. Case-ascertainment of acute myocardial infarction hospitalizations in cancer patients: a cohort study using English linked electronic health data. Eur Heart J Qual Care Clin Outcomes. 2022;8(1):86-95.

17.	Roe MT, Li S, Thomas L, Wang TY, Alexander KP, Ohman EM, et al. Long-term outcomes after invasive management for older patients with non-ST-segment elevation myocardial infarction. Circ Cardiovasc Qual Outcomes. 2013;6(3):323-32. PubMed PMID: 23652734.

18.	Bahit MC, Kochar A, Granger CB. Post-myocardial infarction heart failure. JACC: Heart failure. 2018;6(3):179-86.

19.	Kochar A, Doll JA, Liang L, Curran J, Peterson ED. Temporal trends in post myocardial infarction heart failure and outcomes among older adults. J Card Fail. 2022;28(4):531-9.

20.	Gouda P, Savu A, Bainey KR, Kaul P, Welsh RC. Long-term risk of death and recurrent cardiovascular events following acute coronary syndromes. PLoS One. 2021;16(7):e0254008.

21.	Witt BJ, Jacobsen SJ, Weston SA, Killian JM, Meverden RA, Allison TG, et al. Cardiac rehabilitation after myocardial infarction in the community. J Am Coll Cardiol. 2004;44(5):988-96.

22.	Wang Y, Leifheit EC, Krumholz HM. Trends in 10-year outcomes among Medicare beneficiaries who survived an acute myocardial infarction. JAMA Cardiol. 2022;7(6):613-22.

23.	Spertus JA, Dawson J, Masoudi FA, Krumholz HM, Reid KJ, Peterson ED, et al. Prevalence and predictors of angina pectoris one month after myocardial infarction. Am J Cardiol. 2006;98(3):282-8. PubMed PMID: 16860010.

24.	Mallik S, Spertus JA, Reid KJ, Krumholz HM, Rumsfeld JS, Weintraub WS, et al. Depressive symptoms after acute myocardial infarction: evidence for highest rates in younger women. JAMA Intern Med. 2006;166(8):876-83. PubMed PMID: 16636213.

25.	Bursi F, Vassallo R, Weston SA, Killian JM, Roger VL. Chronic obstructive pulmonary disease after myocardial infarction in the community. Am Heart J. 2010;160(1):95-101. PubMed PMID: 20598978.

26.	Lopez-Jimenez F, Jacobsen SJ, Reeder GS, Weston SA, Meverden RA, Roger VL. Prevalence and secular trends of excess body weight and impact on outcomes after myocardial infarction in the community. Chest. 2004;125(4):1205-12. PubMed PMID: 15078726.

27.	Asleh R, Manemann SM, Weston SA, Bielinski SJ, Killian JM, Roger VL. Persisting sex-specific differences in outcomes after myocardial infarction: A population-based cohort study. Circulation. 2017;136(Supplement 1). PubMed PMID: 619986615.

28.	Witt BJ, Ballman KV, Brown RD, Jr., Meverden RA, Jacobsen SJ, Roger VL. The incidence of stroke after myocardial infarction: a meta-analysis. Am J Med. 2006;119(4):354.e1-9. PubMed PMID: 16564779.

29.	Maddox TM, Reid KJ, Spertus JA, Mittleman M, Krumholz HM, Parashar S, et al. Angina at 1 year after myocardial infarction: prevalence and associated findings. JAMA Intern Med. 2008;168(12):1310-6. PubMed PMID: 18574088.

30.	Ding Q, Spatz ES, Lipska KJ, Lin H, Spertus JA, Dreyer RP, et al. Newly diagnosed diabetes and outcomes after acute myocardial infarction in young adults. Heart. 2021;107(8):657-66.

31.	Krumholz HM, Normand S-LT, Wang Y. Twenty-year trends in outcomes for older adults with acute myocardial infarction in the United States. JAMA Netw Open. 2019;2(3):e191938-e.

32.	Chamberlain AM, Bielinski SJ, Weston SA, Klaskala W, Mills RM, Gersh BJ, et al. Atrial fibrillation in myocardial infarction patients: Impact on health care utilization. Am Heart J. 2013;166(4):753-9. PubMed PMID: 24093857.

33.	Gerber Y, Melton LJ, 3rd, Weston SA, Roger VL. Association between myocardial infarction and fractures: an emerging phenomenon. Circulation. 2011;124(3):297-303. PubMed PMID: 21709062.

34.	Gerber Y, Weston SA, Enriquez-Sarano M, Berardi C, Chamberlain AM, Manemann SM, et al. Mortality Associated With Heart Failure After Myocardial Infarction: A Contemporary Community Perspective. Circ Heart Fail. 2016;9(1):e002460. PubMed PMID: 26699392.

35.	Jokhadar M, Jacobsen SJ, Reeder GS, Weston SA, Roger VL. Sudden death and recurrent ischemic events after myocardial infarction in the community. Am J Epidemiol. 2004;159(11):1040-6. PubMed PMID: 15155288.

36.	Jabre P, Jouven X, Adnet F, Thabut G, Bielinski SJ, Weston SA, et al. Atrial fibrillation and death after myocardial infarction: a community study. Circulation. 2011;123(19):2094-100. PubMed PMID: 21536994.

37.	Kivimäki M, Batty GD, Singh-Manoux A, Britton A, Brunner EJ, Shipley MJ. Validity of cardiovascular disease event ascertainment using linkage to UK hospital records. Epidemiology. 2017;28(5):735.

38.	Hall M, Dondo TB, Yan AT, Goodman SG, Bueno H, Chew DP, et al. Association of clinical factors and therapeutic strategies with improvements in survival following non–ST-elevation myocardial infarction, 2003-2013. JAMA. 2016;316(10):1073-82.

39.	Dondo TB, Hall M, West RM, Jernberg T, Lindahl B, Bueno H, et al. β-blockers and mortality after acute myocardial infarction in patients without heart failure or ventricular dysfunction. J Am Coll Cardiol. 2017;69(22):2710-20.

40.	King G NR. Why propensity scores should not be used for matching. Polit Anal. 2019;27:435-54.

41.	Ohm J, Skoglund PH, Häbel H, Sundström J, Hambraeus K, Jernberg T, et al. Association of socioeconomic status with risk factor target achievements and use of secondary prevention after myocardial infarction. JAMA Netw Open. 2021;4(3).

42.	Smits PC, Frigoli E, Vranckx P, Ozaki Y, Morice M-C, Chevalier B, et al. Abbreviated antiplatelet therapy after coronary stenting in patients with myocardial infarction at high bleeding risk. J Am Coll Cardiol. 2022;80(13):1220-37.
