# Supplementary material for: Health outcomes after myocardial infarction: A population study of 56 million people in England
Source: PLoS Med. 2024 Feb 15;21(2):e1004343. doi: 10.1371/journal.pmed.1004343 (PMC10868847; doi:10.1371/journal.pmed.1004343)
Supplement: Attachment — Submitted filename: Rebuttal2.docx [file pmed.1004343.s021.docx]

ACADEMIC EDITOR COMMENTS

I have gone through the answers of the authors and revised manuscript. They have done what was in their capacity to address my comments (and I believe those of other reviewers) and made efforts to acknowledge the remaining limitations. The length of the manuscript is justified at least in part by the density of the comments raised by the four reviewers. Having to address them has required from the authors to add substantial new text.

**Many thanks for your thorough and supportive review of the revised manuscript.**

EDITORIAL POINTS

1) While we acknowledge that new text has been added to the revised version of the manuscript since the last revision, the editors agree with reviewer #4 that the text, particularly the discussion, needs to emphasize the key messages more clearly and thus be condensed to improve the readability of the manuscript. In addition, we believe that the reliance on ICD codes and the consequent under-reporting of some comorbid conditions, particularly dementia, should be discussed more clearly as a limitation.

**We have reduced the author summary section of the paper, provided more concise wording throughout the manuscript where possible, with extra focus on condensing the discussion. We have consolidated information as far as possible with more concise wording without losing content added in response to peer-review.**

**We additionally include the following sentence:**

**“We acknowledge that case ascertainment and changes in coding practices may vary by health outcome, and that reliance on ICD coding alone may have led to under reporting of some conditions such as dementia and depression” (ll. 549-551).**

2) Please be consistent in your writing of “long-term/short-term” or “long term/short term”.

**This has been updated throughout to use hyphenation.**

3) Please remove any units from brackets presenting 95% CI values. As the preceding number is presented with a unit, it sufficient to present the 95% CI values as numbers only.

**Apologies – I had misunderstood the guidance provided in the previous review. This has now been rectified throughout the paper and tables.**

AUTHOR SUMMARY

Thank you for providing the Author summary. The current summary is rather long. The bullet points should reflect the core messages/findings for each question. You should aim for 2-3 bullet points for each question with a single sentence per bullet point. Please use non-technical language.

**We have removed technical language and shortened the author summary as per the guidance, which now reads as follows:**

***Why was this study done?***

1) *Myocardial infarction (MI; heart attack) can have major long-term impact on individuals and result in a wide range of further health conditions.*

2) *Existing studies have focussed on determining the short-term risk of a second heart attack, stroke or major bleeding, but research describing the long-term risk of major health outcomes for specific age, sex and deprivation groups was lacking.*

3) *Nationally representative and robust information of a wide range of long-term health outcomes following a heart attack is critical for the development of treatment recommendations which take account of an individuals’ specific risk.*

***What did the researchers do and find?***

1) *From the population of 56 million adults in England, we analysed hospital records for 34 million adults admitted to hospital (constituting 145 million admission records) to investigate the long-term health outcomes following a heart attack compared with individuals without a heart attack.*

2) *Of 433,361 individuals with a heart attack - up to a third developed heart failure or renal failure, 7% had further heart attacks and 38% died within the 9-year study period.*

3) *Heart failure, atrial fibrillation, stroke, peripheral arterial disease, severe bleeding, renal failure, diabetes and depression occurred more frequently for people who had a heart attack compared with those who did not; but the risk of cancer was lower overall and the risk of dementia did not differ overall.*

***What do these findings mean?***

1. *Efforts should be made to prevent or limit the development of long-term health outcomes which follow a heart attack – the likelihood of which differ depending on the age, sex and deprivation of an individual.*
2. *These findings are based on the full population of adults admitted to hospital in England, address limitations of previous studies and can be used to inform preventative strategies tailored to specific individuals surviving a heart attack.*
3. *The study was limited to hospitalisation data only – therefore some diagnoses made outside of hospital may have been missed.*

INTRODUCTION

1) l.143: Please add “To our knowledge, there were no…”

**This has been updated (l.138 of the revised manuscript).**

2) l.147: Please add “To our knowledge, published articles to date have..”

**This has been updated (l.143 of the revised manuscript).**

3) ll.166-171: There is no need to list all health outcomes in full more than once in the paragraph. We suggest shortening this to: “Therefore, we determined the excess incidence, estimated the adjusted absolute risk, and presented age, sex, deprivation and time specific risk charts for each of these health outcomes following MI compared with matched controls.”

**This has been updated (ll.162-165 of the revised manuscript).**

METHODS AND RESULTS

1) l.341: Please define ‘SD’ before first use.

**Definition has been added (l.320-321 of the revised manuscript).**

2) ll.379-383: Please ensure that C

I values are presented in square brackets and that before each set of square brackets “95% CI” is added.

**The omitted brackets and “95% CI” have been added in (l.380 of the revised manuscript).**

3) ll.382 and ff: “There was an excess rate of heart failure (adjusted HR 4.93; 95% CI 4.89,7.97;p<0.001)…”. In the following brackets, you only refer to “HR”. Are the values that follow for the other outcomes also adjusted hazard ratios? We feel the current presentation might be confusing to readers. We suggest introducing the abbreviation "aHR" on line 267 and using "aHR" from then on. Please revise.

**These do indeed all refer to adjusted hazard ratios. I have introduced the aHR abbreviation in the methods section (l.260), and used this abbreviation where appropriate throughout the manuscript.**

4) Figure 2: Please change the x-axis label and the column header to “adjusted Hazard Ratio”. You have already defined ‘CI’ in the figure description (“…and 95% confidence intervals (CIs)..”); please remove the definition of ‘CI’ below the description.

**The figure description and in figure labels and headers have been updated accordingly.**

DISCUSSION

Please remove the ‘Conclusion’ heading. The conclusion paragraph should be part of a continuous discussion.

**Removed as requested.**

REFERENCES

When specifying the date of access, please write “accessed” instead of “Date accessed”.

**Updated throughout.**

SUPPLEMENTARY MATERIAL

1) S1 and S2 Checklist contain the same checklist. Please check and provide both, the RECORD checklist and the CODE-EHR checklist.

**Apologies for the incorrect upload – the correct CODE-EHR checklist has now been uploaded for S2.**

2) S2 Figure: The definition of footnote c is missing in the figure description.

**The following footnote has now been added to the figure description: “^c^includes all cancer types (ICD10 codes C00-C97), i.e. this category is not restricted to the sum of breast, prostate, lung and colorectal cancer).”**

3) S2 Table: You have already defined ‘ICD’ and ‘OPCS’ in the table title, i.e. you can remove these two definitions from the definitions listed underneath.

**These have been removed as requested.**

3) S3/S4 Table: Similarly, to S2 Table, please note that you only need to define abbreviations once, either in the title or in the definitions below the table (also applies for figures). Please revise throughout the entire main manuscript and the supporting files.

**I have updated the following:**

- **pyrs abbreviation removed from S3/S4 Table**
- **“Hazard Ratio” for S8 Table has been updated to adjusted Hazard Ratio (aHR) in figure description and column headers, and the duplicate definition of this abbreviation removed**
- **Duplicate CIF description removed from Fig 4 and S3, S4 and S5 Figures.**

SOCIAL MEDIA

To help us extend the reach of your research, please provide any X (formerly known as Twitter) handle(s) that would be appropriate to tag, including your own, your coauthors’, your institution, funder, or lab. Please respond to this email with any handles you wish to be included when we tweet this paper.

**These are provided via email as requested.**

Comments from Reviewers:

Reviewer #2:

Reviewer 2

I appreciate the authors detailed responses to the issues previously raised and believe that the majority of these have been resolved.

However, for comment 3 although the text has been changed to "time dependent effect for cohort" and this reflects the terminology used in the referenced paper for the standsurv function, I think this is unusual as "time-updated" and "time-dependent" are more often used for the same meaning of coefficients changing over time as opposed to the model estimates . While the aims of approach taken do seem logical given the potential non proportional hazards over time, with using continuous time for the model as opposed to categorical breaks as in the standsurv publication, it isn't obvious at what (if any) time-points these hazard ratios would apply to if the estimates could end up higher/lower at different times during follow up. If the interpretation is just intended as an average or some other meaning if they are not interpretable the same way as a more conventional survival analysis, could be briefly expanded on within methods.

**Thank you for the supportive review of our revised manuscript.**

**We would like to clarify that our adjusted hazard ratios (Fig 2, S8 Table) are not time-dependent and are fixed to provide an overall average estimate of excess risk. As such these estimates are interpreted in the traditional way and provide a useful comparator with existing literature. In addition to the fixed adjusted hazard ratio, we provide cumulative incidence functions presented graphically over continuous time (Fig 3A, Fig 3B) which are modelled with the time dependent effect. This allows relaxation of the proportional hazards’ assumption, and to model CIFs which vary over continuous follow up time - therefore providing higher resolution insight over and above that achieved through the non-time-dependent hazard ratios (e.g. for dementia where there is an increased risk in the short-term post MI, and a reduced risk in the long-term – averaging out as no effect overall).**

**We feel it is important to present both analyses so the difference and added benefit of time-dependent effects may be realised. Whilst we understand the issue around terminology – we feel it is important to remain consistent with the referenced paper in which the methods are developed to enable others to understand and replicate our approach which will aid further uptake of this relatively modern technique.**

**We have added the following to the methods to clarify our two approaches more directly in the paper:**

***“To provide an overall estimate of excess risk of post MI disease, comparable with existing literature, adjusted HRs were modelled as standard proportional hazards (i.e. fixed constant over time). Subsequently, the proportional hazards assumption was relaxed to provide higher resolution insight into absolute risk of outcomes over continuous follow up time (described below).”***

**and**

***“….time-dependent effect for cohort (MI versus matched controls) to relax the proportional hazard assumption and to reflect variation in the difference in cumulative incidence between cohorts over continuous follow-up time.“***

Reviewer #4: The authors have made a robust effort to address the comments from the reviewers. My main residual comment is the presentation of the data and the writing of the manuscript. In my opinion, the text is too long and the message therefore gets diluted. A deliberate commitment to being more concise would strengthen the message of the manuscript and make it easier for the readership.

**Many thanks in reviewing our revised manuscript. We had a significant challenge in including recommendations from 4 reviewers and 2 editors whilst keeping concise – but have made a thorough effort to revise our manuscript throughout, changing some sentence structures and consolidating information into more concise wording. As per the editorial guidance we focussed predominantly on the author summary (condensed by half) and the discussion (condensed by ~ 500 words). We consolidated existing material as far as possible without removing core content highlighted for inclusion by the peer review process.**
